# Diet Outweighs Vertical Transmission in Shaping Dung Beetle Larval Gut Microbiomes

**DOI:** 10.1111/mec.70336

**Published:** 2026-04-03

**Authors:** Michelle J. Herrera, Avi Khanna, Joshua A. Jones, Ricardo Betancur‐R., Patrick T. Rohner

**Affiliations:** ^1^ Department of Ecology, Behavior, and Evolution University of California, San Diego San Diego California USA; ^2^ Marine Biology Research Division, Scripps Institution of Oceanography University of California, San Diego San Diego California USA; ^3^ Department of Entomology University of Minnesota Minneapolis Minnesota USA

**Keywords:** diet, dung beetles, hindgut, microbiome, organism‐environment interactions

## Abstract

The microbiome is central to host development and adaptation, yet the balance between vertical and environmental acquisition, and how hosts shape surrounding microbial communities, remains poorly understood. Dung beetles rely on microbial symbionts to extract nutrients from vertebrate dung, with part of their microbiome vertically inherited via a maternal faecal pellet. However, the relative importance of vertical versus horizontal transmission is unclear. We examined this in the gazelle dung beetle (*Digitonthophagus gazella*), rearing larvae on brood balls made of dung from grass‐(high‐quality), hay‐(low‐quality) or silage‐fed (a novel fermentable energy‐rich diet) cattle, with or without maternal microbes. We integrated measures of gut morphology with 16S rRNA amplicon sequencing to assess host development and the gut microbiome. Diet significantly influenced overall size, hindgut area, and microbiome composition. Silage‐dung fed larvae had more even and taxonomically rich microbiomes, with higher microbial diversity in individuals reared with maternal microbes. Diet explained ~26% of the variation in microbial composition, while the vertical transmission of microbes only explained 3%. Vertical transmission only slightly increases microbial species richness and relative hindgut area but did not influence overall microbial diversity. The larval brood ball contributed 40%–50% of the hindgut microbiome, while maternal microbes contributed < 0.05%. These findings demonstrate that horizontal acquisition through diet is the dominant force shaping larval gut microbiomes, while vertical inheritance plays a minor but detectable role in enhancing richness and gut development. More broadly, this work reinforces the importance of examining host–microbiome–environment interactions in ecological and evolutionary contexts.

## 1 Introduction

The microbiome within an organism's digestive system, as well as its immediate environment, plays a crucial role in an organism's ability to adapt to novel resources (McFall‐Ngai et al. 2013; Bordenstein and Theis 2015). Microbial symbionts help hosts digest nutritionally challenging foods by fermenting otherwise indigestible complex carbohydrates into short‐chain fatty acids that the host absorbs and metabolizes (Van Soest 1994). While most microbiome studies focus on vertebrates, invertebrates make up the majority of extant species and exhibit diverse life history traits that shape the composition and diversity of microbial symbionts (Douglas 2011; Petersen and Osvatic 2018). In insects, the gut microbiome is shaped by a dynamic interplay of factors, including host diet (Colman et al. 2012; Franzini et al. 2016; Shukla et al. 2016), developmental stage (Chen et al. 2016; Jones et al. 2024), horizontal transmission of microbes from the environment (Ng et al. 2018) and vertical transmission of microbes from parents to offspring (Wang and Rozen 2017; Estes et al. 2013). Yet, the degree to which these communities can shift when hosts encounter novel resources or environments remains poorly understood.

Dung beetles (Scarabaeinae) provide a unique system for examining the flexibility of the microbiome due to their ecological diversity, symbiotic relationships and life history traits. By burying and consuming dung, they provide key ecosystem services such as nutrient cycling and soil improvement in agricultural landscapes (Nichols et al. 2008; Anderson et al. 2024). Onthophagine dung beetles dig tunnels underneath cow pats and construct compact underground dung structures, known as ‘brood balls’ (Hanski and Cambefort 1991) that each provision a single offspring. Larvae hatch inside these brood balls and rely exclusively on them for nutrition, making brood ball size and quality critical determinants of growth and development (Hunt and Simmons 2004; Kishi 2014). For instance, access to high‐quality dung results in larger adult body size and more exaggerated male secondary sexual traits (Moczek 1998). Since dung is rich in recalcitrant fibres, larvae may depend on gut symbionts to help digest this cellulose‐heavy diet (Rougon et al. 1990; Byrne et al. 2013; Shukla et al. 2016).

Alongside the egg in the brood ball, mothers deposit a faecal inoculate, called a ‘pedestal’ (or maternal gift; see Shukla et al. 2016), which is thought to vertically transmit beneficial microbes (Estes et al. 2013). Thus, larvae ingest the pedestal, the brood ball itself and their own excrement, offering a unique framework for unravelling the contributions of inherited microbes from environmentally acquired microbes. Despite its small size, withholding or exchanging the pedestal with that of a different species results in negative fitness consequences, including increased phenotypic variance and decreased growth, development and survival rate (Byrne et al. 2013; Schwab et al. 2016; Parker and Moczek 2020; Parker et al. 2019; Parker et al. 2021; Rohner and Moczek 2024). This suggests that certain vertically transmitted microbes are both beneficial and passed on from one generation to the next in a species‐specific manner. This raises the possibility that microbiomes generate heritable variation among lineages, thereby shaping phenotypic diversity and modulating evolutionary responses to selection via ecological inheritance.

In addition to the microbes that are provided via the brood ball and the maternal pedestal, larval behaviour further structures the microbial community of the brood ball. Larvae defecate within the brood ball, and re‐ingest the resulting faeces‐enriched mixture, potentially facilitating microbial fermentation both in their gut and within the external brood ball environment. The microbiome of brood balls is distinct from that of unprocessed cow dung (Jones et al. 2024) and brood balls that are inhabited by a larva are enriched for microbial taxa able to break down complex carbohydrates (Schwab et al. 2017). Larvae that modify their brood ball grow faster and emerge as larger adults, suggesting that larvae engineer their microbial environment to benefit host development (Schwab et al. 2017). Based on these observations, it has been hypothesized that dung beetle larvae construct an external rumen, where microbial symbionts outside the gut help pre‐digest their recalcitrant diet (Halffter 1997; Holter 2016; McConnell and Rohner 2025). Thus, it is expected that larvae actively shape the microbiome of their brood ball in ways that influence larval growth and development. The dynamics and function of these microbial communities and whether larval behaviour supports the establishment of horizontally acquired microbes remain poorly understood.

For most of their evolutionary history, dung beetles have encountered dung from herbivores that consume grass or hay and dung beetle evolution is tightly associated with the diversification of Artiodactyls (Ahrens et al. 2014). Herbivore dung is typically rich in structural polysaccharides, such as cellulose and lignin (Holter 2016). However, in modern agricultural systems, cattle diets have shifted from grass and hay to a mix with maize silage. In the United States, maize silage has become one of the dominant components of dairy rations and in 2016, 55% of dairy farms report feeding corn silage, which contributed about 30% of the cows' dietary dry matter (Gillespie 2023). Maize silage is a processed feed that is high in starch and fermented carbohydrates, which reduces recalcitrant fibre content (Keady et al. 2008; Khan et al. 2012; Khan et al. 2015). Silage likely harbours a distinct microbial community compared to the more fibrous grass and hay dung (Mulligan et al. 2002; Hindrichsen et al. 2006). These changes likely create a novel nutritional landscape for dung beetles that differs both chemically and microbially from the dung they have historically encountered. How dung beetle larvae and their microbiomes adjust to a nutritionally and microbially altered dung remains unknown. Given that larvae have well‐developed chewing mandibles and host symbionts that facilitate digestion of solid, fibrous parts of dung, they may exhibit physiological plasticity in response to dung of differing quality and composition (Byrne et al. 2013; Shukla et al. 2016). Indeed, larvae reared on particularly fibrous dung develop larger midguts and exhibit longer gut retention times (Rohner and Moczek 2021), indicating that the digestive system can adjust to changes in dietary components.

**FIGURE 1:**
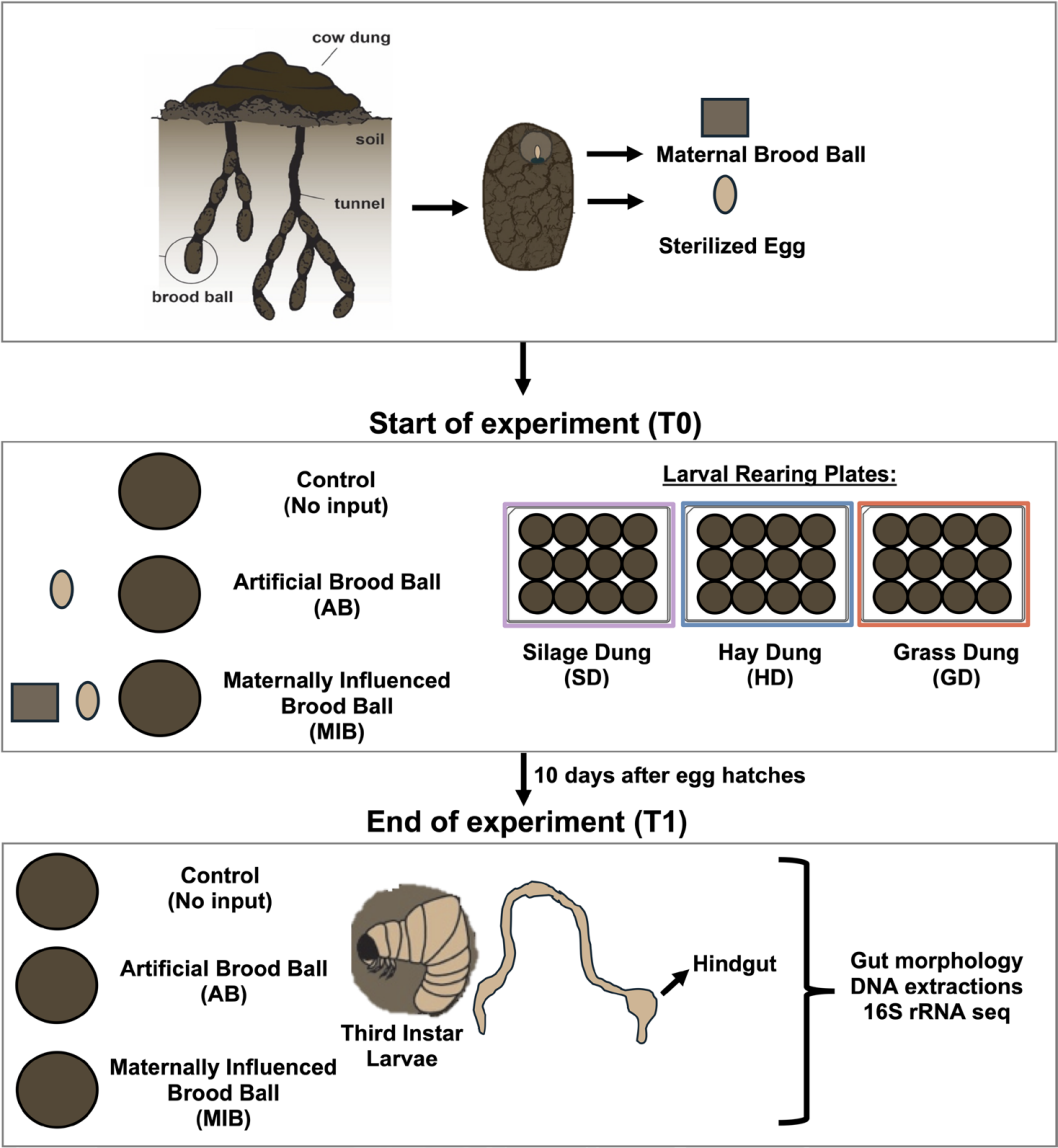
To manipulate diet and maternal microbiome treatment, we placed sterilized eggs in larval rearing 12‐well plates with artificial brood balls made of either silage dung (SD), hay dung (HD), or grass dung (GD). There were two control brood balls in each plate that were sampled at the start and end of the experiment (T0 Control and T1 Control). We sampled sterilized eggs and a portion of the maternal brood ball at the beginning of the experiment. With the remaining artificial brood balls, half were made without the presence of maternal microbes (artificial brood ball or AB), while the other half had a portion of the maternal brood ball (maternally influenced brood ball or MIB). At the end of the experiment, we dissected the third instar larvae, took images of the gut, and sampled larval hindguts, AB and MIB larval brood balls, and the T1 Control.

Here, we aimed to characterize how vertical and horizontal acquisition shape the larval gut microbiota composition of the dung beetle *Digitonthophagus gazella* and how this interacts with larval survival, gut morphology and the microbiome composition of the immediate environment. Specifically, we aimed to answer the following questions: (1) How does dung from cows fed silage, grass or hay affect the gut size and hindgut microbiome of dung beetle larvae? (2) Does the presence or absence of vertical microbial transmission shape the larval microbiome? and (3) How do larvae actively shape the microbiome in their immediate environment? We focused on the larval hindgut microbiome because it is the compartment that harbours the largest microbial population (Douglas et al. 2020) and because larvae develop an enlarged hindgut resembling a fermentation chamber, suggesting that microbial fermentation is integral to larval digestion (Halffter and Matthews 1971). Our experimental setup is depicted in Figure 1.

We hypothesize that larval gut morphology and microbiome respond to the experimental manipulation of diet and the presence of maternal microbes. Specifically, silage dung, being the most compositionally novel and potentially stressful diet, should yield delayed gut development with smaller guts and reduced microbiome diversity; hay dung, being fibre‐rich but more familiar to dung beetles, should produce moderate gut sizes with fibre‐degrading microbes; and grass dung, representing the highest quality and evolutionary typical diet, should promote faster growth, larger guts and a diverse, balanced microbiome dominated by typical fermenting microbial taxa such as Bacillota. However, we also consider the alternative hypothesis that the fermented nature of silage could conversely promote microbial diversity by providing readily accessible substrates for microbial colonization. As the lack of typically vertically inherited maternal microbes would be challenging for the larvae, we predict that larvae reared without maternal microbes will develop smaller guts and lower gut microbiome diversity. Understanding how dung beetle larvae and their gut microbiomes respond to shifting nutritional landscapes will provide insight into how microbiomes facilitate host resilience when encountering novel resources.

## 2 Methods

### 2.1 Sample Collection and Preparation

80 individuals of *Digitonthophagus gazella* (Fabricius, 1787) were collected in 2024 from Bastrop County, Texas and sent to University of California, San Diego and kept under standard laboratory conditions (Moczek and Nijhout 2002). The laboratory colony was maintained at 28°C and fed twice a week with fresh, previously frozen cow dung. To generate offspring for experimental manipulations, we placed 5 adult females into a breeding chamber that was filled with a compact mixture of sand and soil and topped off with ca. 500 g of fresh, previously frozen dung of cows feeding on a mixed diet containing hay and silage. Breeding chambers were incubated for 6 days at 28°C. Thereafter, they were deconstructed, and the maternally provisioned brood balls were sifted from the soil mixture. Eggs were removed from the maternal brood ball using flame‐sterilized forceps and then surface‐sterilized with 200 μL of a 1% bleach and 0.1% Triton‐X 100 solution, followed by two rinses with deionized water, as established previously (Estes et al. 2013). A subset of sterilized eggs was placed individually in sterile 1.7 mL microcentrifuge tubes and immediately frozen in −80°C freezer for downstream DNA extractions and sequencing. The rest of the sterilized eggs were transferred to experimental brood balls and assigned to their respective diet and maternal treatment groups, as described below (Figure 1). Samples of the maternal brood ball (‘T0 Maternal Brood Ball’) were taken by scraping the wall of the brood chamber with flame‐sterilized forceps and then placed into a sterile 1.7 mL microcentrifuge tube that was immediately frozen in −80°C freezer for downstream DNA extractions and sequencing. The rest of the samples of the T0 Maternal Brood Ball were used in experimental diet and maternal treatment groups.

To manipulate the larval diet and the presence of maternal microbes, we raised offspring in artificial brood balls by squeezing by hand previously frozen cow dung through a sterile cheesecloth to remove excess liquid. Then, dung was rolled into balls of 3.2 g to fill the wells of sterile 12‐well tissue culture plates (Rohner et al. 2021). Each artificial brood ball in a well then represents an experimental rearing environment for a single egg. To manipulate larval diet, the artificial brood balls were made with cow dung collected from cattle that were either fed with hay, grass, or a mixed diet that mostly consisted of silage (i.e., a mixture of fermented corn and grass). Throughout the manuscript, we refer to these dung diets as silage dung (SD), hay dung (HD) and grass dung (GD). The larval rearing plates were closed with a lid and kept at a constant temperature in a 26°C incubator.

Each 12‐well plate contained distinct treatments that manipulated the presence of maternal microbes, which we refer to throughout the manuscript as ‘treatment’. To manipulate vertical transmission, sterilized eggs were placed in an artificial brood ball that had one of two treatments: artificial brood ball (AB), which lacked any maternal input or maternally influenced brood ball (MIB), in which a ~3 mm^2^ portion of a T0 Maternal Brood Ball sample was placed in the artificial brood ball (Note that, although this is a small amount of material relative to the size of the brood ball, this manipulation has previously been shown to affect the development time and adult size of *D. gazella* in a sex‐specific manner; Rohner and Moczek 2024), implying downstream effects on larval development driven by small amounts of vertically‐inherited microbes (also see Schwab et al. 2016). Ten of the wells in the plate were used to rear larvae, with five wells randomly assigned to the AB treatment, and five to the MIB treatment and evenly distributed within larval rearing plates across dung types. Two artificial brood balls were left empty (i.e., without an egg) and used to test how microbial communities change over time in the absence of larval activity. One of these empty artificial brood balls was sampled at the start of the experiment (‘T0 Control’), and the other after 10 days of incubation under conditions experienced by the larvae in the same plate (‘T1 Control’). Because these artificial brood balls were never inhabited by a larva, any differences between T0 and T1 controls are driven by microbial dynamics that are expected to occur across time in the artificial brood balls alone. The AB and MIB brood balls that inhabited larvae were also sampled at the end of the experiment after 10 days post egg hatching.

The larval rearing plates were checked daily until the egg hatched. Then, 10 days post‐hatching at the third instar larval stage, larva were euthanized with 100% ethanol and dissected under a microscope in a sterile petri dish with PBS and 0.1% Triton‐X 100. The digestive system was removed by gently uncoiling the gut and pictures were taken with an Olympus Touch TG‐6 camera in a photo light box to measure gut size in ImageJ (see ‘Gut and body size measurements’ section). The hindgut of the larvae was excised, placed in sterile 1.7 mL microcentrifuge tubes and frozen immediately in a −80°C freezer until DNA extractions (see ‘Gut microbiome analysis for all samples’).

### 2.2 Gut and Body Size Measurements

To estimate overall larval size, we took a size‐calibrated image of each sacrificed larva before it was dissected and calculated the total area of the larva in lateral view in ImageJ (version 1.54 g) (see Figure S1; note that this measure correlated well with other measurements of larval size, such as the summed distance between all spiracles). The larva was outlined by hand via a Wacom Intuos drawing tablet in order to increase both efficiency and precision of the measurements taken.

After the digestive tract was dissected, we took another photograph and measured the respective areas of the fore‐, mid‐ and hindgut segments (Rohner and Moczek 2021), which serve different functions in the digestion process of insects and dung beetles (Douglas et al. 2020; Ebert et al. 2021). To reduce measurement biases, gut measurements were conducted blind to the diet and maternal treatment conditions of each specimen.

All statistical analysis was conducted using R (Version 4.4.0). Analysis of covariance (ANCOVA) was used to statistically assess whether diet or maternal treatment manipulations affected the size of each gut segment relative to body size. Specifically, we fitted log‐transformed gut segment area as a function of log‐transformed body area (as covariate), the main effects of diet and maternal treatment, as well as their interaction. Non‐significant interactions were removed, following standard backwards elimination procedures. Partial eta squared was calculated as effect size as implemented in the R package *effectsize* (Ben Shachar et al. 2020).

### 2.3 Gut Microbiome Analysis for All Samples

The sample DNA was isolated from the larval hindgut, larval brood ball (‘AB’ or ‘MIB’), sterilized egg, T0 Control, T1 Control and T0 Maternal Brood Ball using the Zymobiomics DNA mini kit from Zymo Research. 16S rDNA amplicon PCR was performed targeting the V3–V4 region (using 341F and 806R primers) (Klindworth et al. 2013). Using miseq v3 chemistry (PE300 sequencing length), the libraries were sequenced at the UC Irvine Genomics Research and Technology Hub (GRTH). We sequenced 101 samples, including a mock community sample in the library. This resulted in 14,968,764 (~15 million) reads, with 9,919,939 passing filter (15% phiX spike in) with an overall > Q30. The raw sequences were imported into QIIME2 (‘Quantitative Insights Into Microbial Ecology’ version 2024.10) using UCI's High Performance Community Computing Cluster (HPC3). After initial sample quality check (99% identity threshold), the paired‐end sequences were quality filtered using the DADA2 pipeline in QIIME2, resulting in 3,588,450 merged paired‐end reads.

Taxonomic classification for Amplicon Sequence Variants (ASVs) was assigned using the Silva 138 99% operational taxonomic unit (release 138) that we trained for the 341F/806R region of our sequences (Quast et al. 2013). Analyses were conducted in both QIIME2 and R (Version 2024.04.1+748). Prior to alpha‐ and beta‐diversity analyses, samples were rarefied to an equal sequencing depth within each dataset analysed. Rarefaction depths were selected based on inspection of rarefaction curves to balance sequencing depth and sample retention, corresponding to points at which diversity curves began to level off (Figure S2). Hindgut samples used in hindgut‐specific analysis were rarefied to 7700 reads per sample. Brood ball samples used in brood ball‐specific analysis were rarefied to 4299 reads per sample. For analyses combining all samples, we rarefied to a single common depth of 4200 reads per sample to enable direct comparison across sample types. These rarefaction depths retained all samples in each dataset, while capturing stable diversity estimates.

We used ANOVA followed by Tukeys honestly significant difference (HSD) to determine whether there were significant differences in alpha diversity (Shannon's alpha diversity and Chao1 richness index) of the microbial communities. Factors affecting gut size, larval body size and alpha diversity were evaluated with ANOVA, including dung diet (SD, HD, or GD), maternal treatment (AB or MIB) and their interaction as fixed effects. Post hoc pairwise differences among sample types were assessed using Tukey's HSD. When an interaction was not significant, it was removed from the ANOVA model. Bray–Curtis dissimilarity matrices were calculated using rarefied ASV‐level abundance data and used to generate non‐metric multidimensional scaling plots for microbial communities. PERMANOVA with Benjamini‐Hochberg *p*‐adjusted values were used to test for differences in microbial community beta diversity.

SourceTracker2 package in QIIME2 using a Gibbs sampler (Markov Chain Monte Carlo algorithm) was performed to estimate the relative proportional contribution of pre‐determined ‘sources’ to each ‘sink’, separated by diet and maternal treatment (Knights et al. 2011). One analysis was conducted with the larval hindgut as a sink and the sources as the larval brood ball (AB or MIB), sterilized egg, T0 Control, T1 Control or T0 Maternal Brood Ball. A second analysis was conducted with the larval brood ball as a sink and the sources as the larval hindgut, sterilized egg, T0 Control, T1 Control, or T0 Maternal Brood Ball.

To determine shared and unique ASVs among different sample types, we created Venn Diagrams in R. To visualize changes in microbial community abundance, we averaged the relative abundance of replicates for samples in each diet and maternal treatment and created stacked bar plots in R. ANCOM as a plugin in QIIME2 was used to determine the significant ASVs that are enriched in different sample types based on log fold change.

To identify microbial taxa driving group‐level differences, we ran indicator species analysis, which determines microbial taxa that are uniquely associated with particular diets or maternal treatments (De Cáceres et al. 2012). To interpret patterns in the non‐metric multidimensional scaling (NMDS) plots, we overlaid vectors representing microbial features significantly correlated with group separation (*p* = 0.005; https://riffomonas.org/code_club/2022‐04‐11‐biplot). Finally, to determine core microbial taxa shared among all hindgut samples, we utilized the core‐features command in QIIME2 to identify ASVs observed in 100% (fraction of 1.0) of all samples. These ASVs were identical among all samples.

To predict functional pathways based on the 16S rRNA sequencing data, we used the Phylogenetic Investigation of Communities by Reconstruction of Unobserved States (PICRUST2) pipeline (Douglas et al. 2020), and the ‘ggpicrust2’ R package (Yang et al. 2023) for statistical analysis and visualization of results. We performed a principal component analysis to investigate the overall differences in the distance of Kyoto Encyclopedia of Genes and Genomes (KEGG) functional pathways. We conducted differential abundance analyses to determine the significant KO IDs and KEGG pathways between sample groups based on log fold change (LFC).

All data generated were deposited into NIH Archive with Bioproject accession number PRJNA1338251 and microbiome and R scripts are located in a GitHub repository: https://github.com/mjherre1/D.gazella_microbiome.git.

## 3 Results

### 3.1 Plastic Responses in Digestive Morphology and Larval Body Size

**FIGURE 2:**
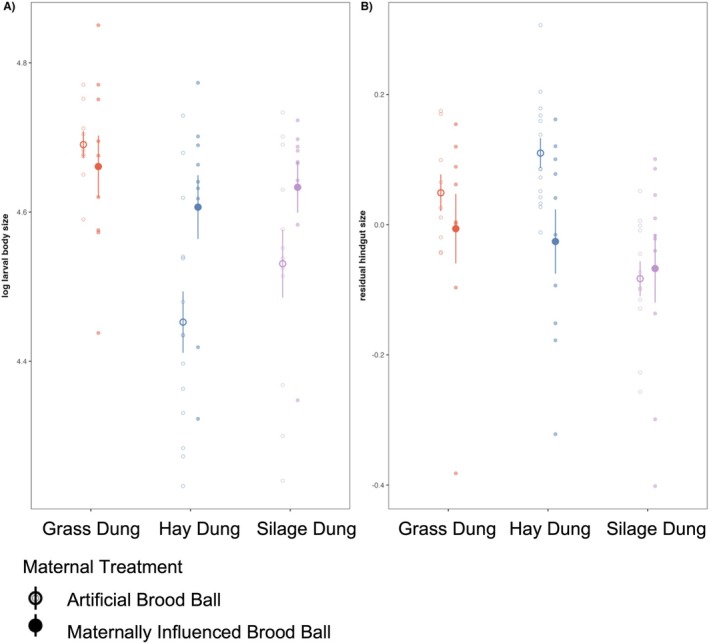
(A) Larvae fed grass dung (GD) were larger (log larval body size) than larvae fed silage dung (SD) or hay dung (HD). Larvae reared with a portion of the maternal brood ball (MIB), had higher body size particularly in HD and SD larvae. (B) Residual gut sizes (i.e., gut size relative to body size) of larvae differ by diet and maternal microbial treatment. Larvae fed GD and HD had larger hindguts compared to those fed SD. Residuals are derived from a linear regression of log trait size against log larval body size. Error bars indicate 95% confidence intervals.

Out of 56 total larvae for each diet group, mortality rates were 12.5% and 14.3% for silage‐fed AB and MIB larvae, 7.1% and 0% for hay‐fed AB and MIB larvae and 14.3% and 25% for grass‐fed AB and MIB larvae, respectively. Larval diet had a significant effect on larval body size, and GD‐fed larvae were larger than their SD or HD counterparts (*F*_2,60_ = 7.23, *p* = 0.002, η_p_^2^ = 0.19 [0.04, 0.36] 95% confidence limits) (Figure 2a). The presence of a maternal microbial inoculate, or MIB larvae, also increased larval body size, although to a lesser degree compared to the effect of diet (*F*_1,60_ = 6.16, *p* = 0.016, η_p_^2^ = 0.09 [0.00, 0.25]). This effect seemed to be less pronounced in GD‐fed larvae, but there was no statistical support for an interaction (*p* = 0.085).

In addition to body size, diet also influenced the relative size of the gut. This was evident for the relative size of the entire gut (*F*_2,59_ = 10.70, *p* < 0.001, η_p_^2^ = 0.27 [0.08, 0.43]), as well as for each of the three sections analysed separately (Foregut: *F*_2,59_ = 7.46, *p* = 0.001, η_p_^2^ = 0.20 [0.04, 0.37]; Midgut: *F*_2,59_ = 4.87, *p* = 0.011, η_p_^2^ = 0.14 [0.01, 0.30]; Hindgut: *F*_2,58_ = 6.23, *p* = 0.004, η_p_^2^ = 0.17 [0.02, 0.34]) (Figure 2b, Figure S3). The presence of maternal microbes reduced the relative size of the hindgut (*F*_1,59_ = 4.25, *p* = 0.044, η_p_^2^ = 0.07 [0.00, 0.22]) but did not affect the relative size of any other gut compartment (all *p* > 0.05).

### 3.2 Effect of Diet and Vertical Transmission on the Larval Hindgut Microbiome

**FIGURE 3:**
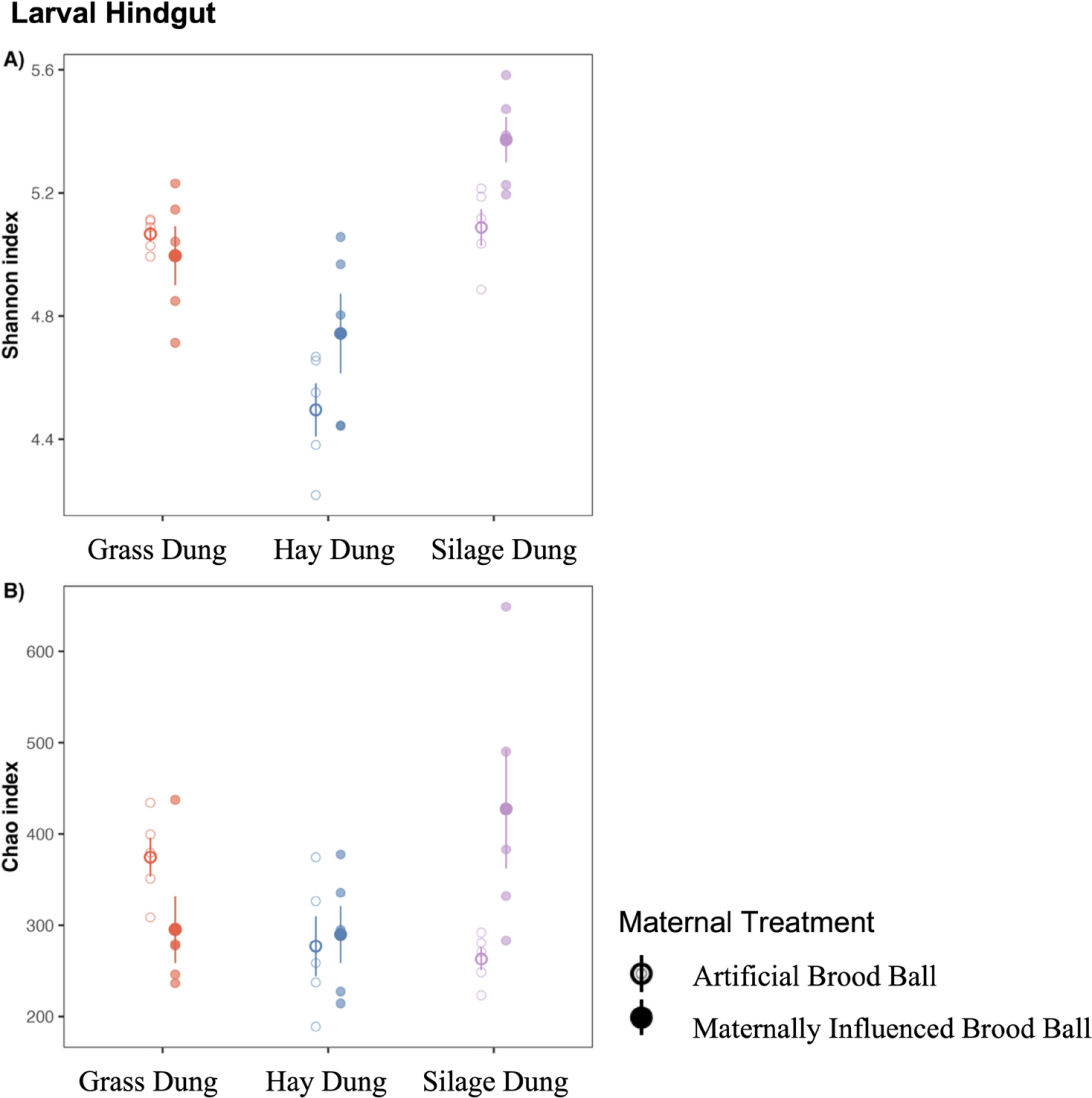
(A) Shannon's alpha diversity index of the hindgut of larvae fed different diets and with or without maternal influence treatment (ANOVA: Diet *F*_*2,24*_ = 27.5; *p* < 0.05; Treatment *F*_*1,24*_ = 4.99; *p* < 0.05). (B) Chao1 richness index of the hindgut of larvae fed different diets and with or without maternal influence treatment (ANOVA: Diet *F*_*2,24*_ = 2.26; *p* = 0.126; Treatment *F*_*1,24*_ = 1.636; *p* = 0.213; Diet*Treatment *F*_*2,24*_ = 5.635; *p* < 0.05).

Diet impacted community composition and overall microbiome diversity. SD‐fed larvae had greater Shannon's alpha diversity, meaning a more even distribution and a larger number of taxa, followed by GD‐fed larvae then HD‐fed larvae (ANOVA: Diet *F*_2,24_ = 27.5; *p* < 0.001; Treatment *F*_1,24_ = 4.99; *p* = 0.035, Figure 3). Larvae reared on silage dung in the maternally influenced brood ball (SD‐fed MIB larvae) had the greatest Chao1 richness (an estimate of the total number of species in the community, adjusted to account for rare species) followed by GD‐fed AB larvae (ANOVA: Diet *F*_2,24_ = 2.26; *p* = 0.126; Treatment *F*_1,24_ = 1.636; *p* = 0.213; Diet × Treatment *F*_2,24_ = 5.635; *p* = 0.010). Based on Tukey's HSD, SD‐fed MIB larvae were significantly different from SD‐fed AB and HD‐fed AB and MIB larvae, indicating that diet was the primary driver of richness, with maternal microbes exerting a significant effect only in combination with silage dung.

**FIGURE 4:**
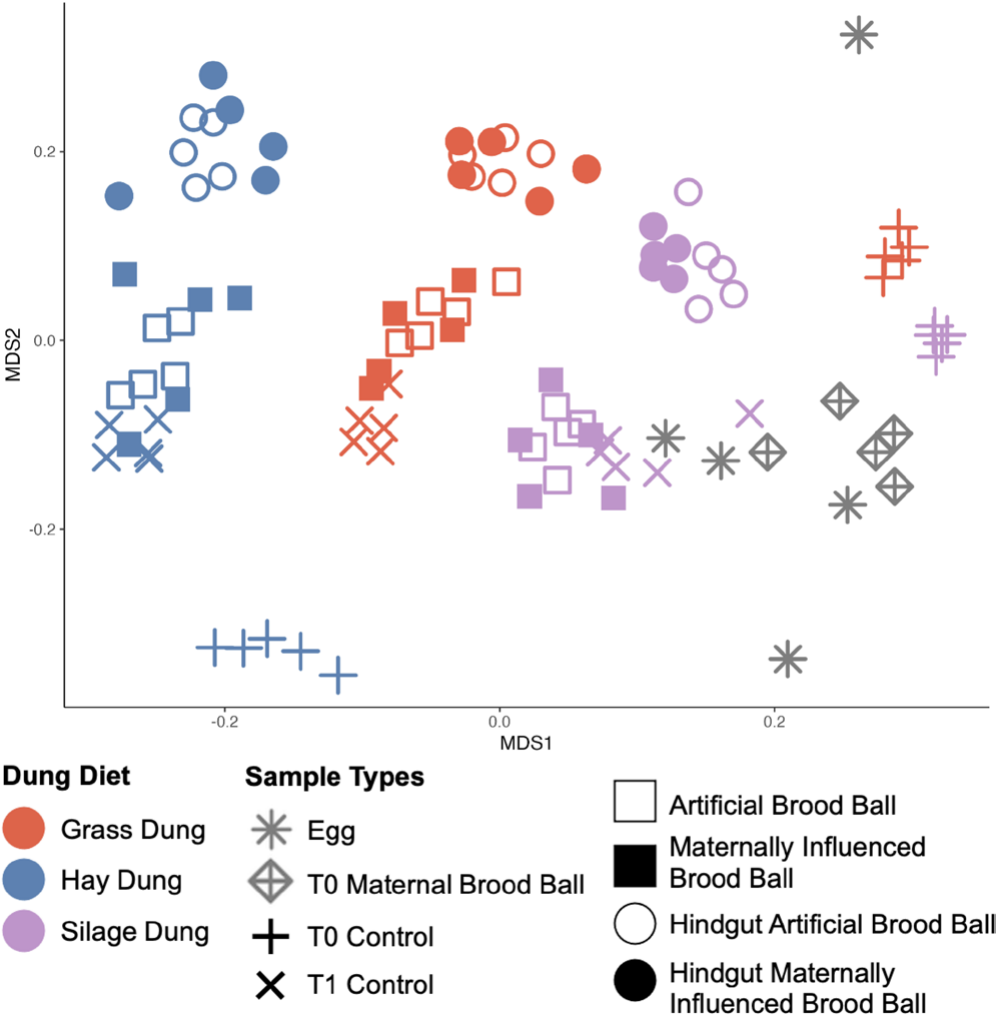
Beta diversity of all samples: Non‐metric multidimensional scaling plot based on Bray–Curtis dissimilarity of the hindgut of larvae fed different diets and with (MIB) or without (AB) maternal influence treatment, AB and MIB larval brood balls, brood ball controls, maternal brood ball, and sterilized eggs. Proximity of data points indicates similarity. Samples that have irrelevant dung diet (T0 Maternal Brood ball and Eggs) are in grey.

Larval hindgut microbial communities clustered by diet in the NMDS plot, with diet explaining 26% of the variation (PERMANOVA, *R* ^2^ = 0.26, *p* = 0.001, Figure 4). The presence of maternal influence explained about 3% of the variation and was not a significant driver of microbial communities (PERMANOVA, *R* ^2^ = 0.04, *p* = 0.125).

**FIGURE 5:**
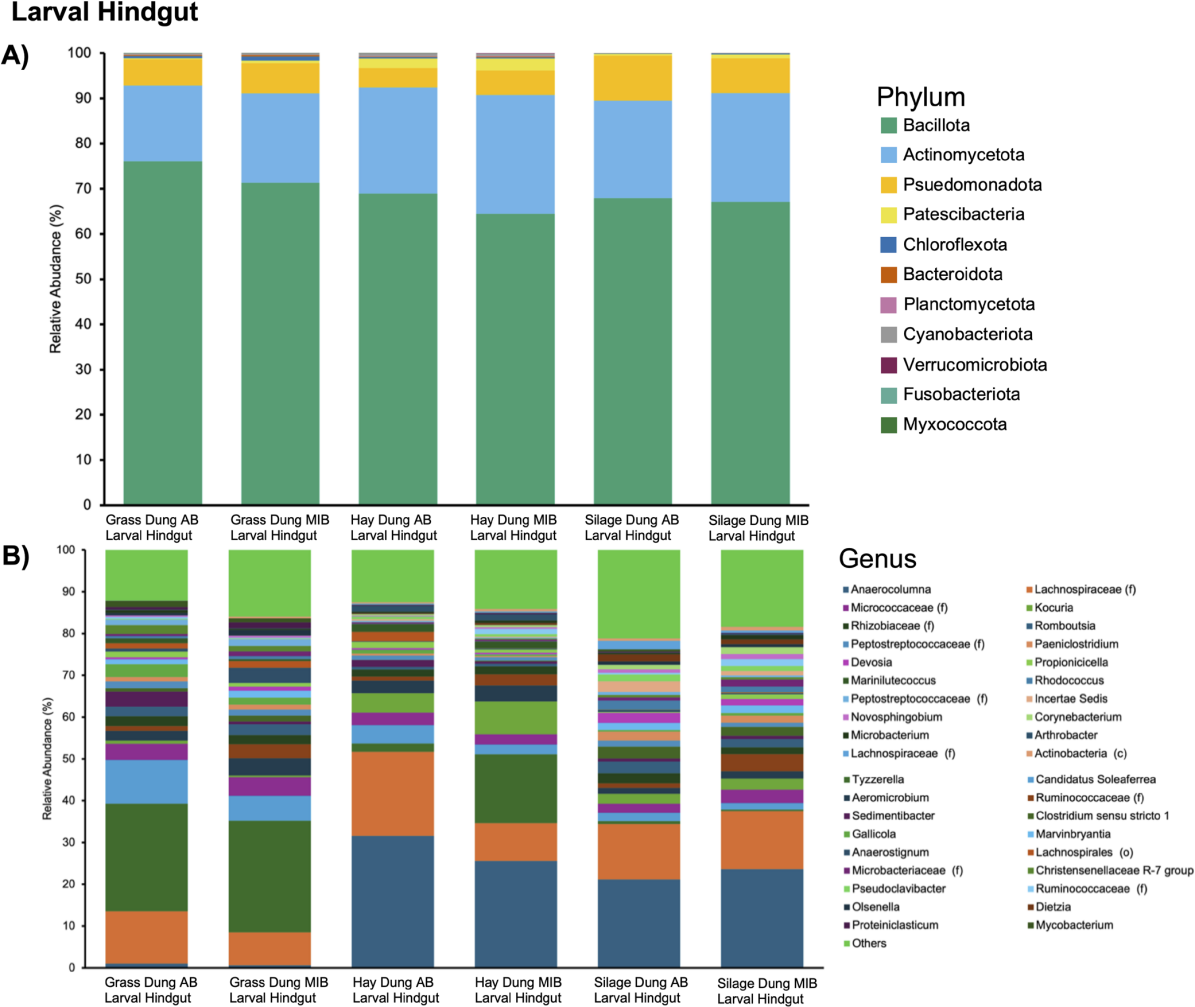
(A) Relative abundance of bacteria, identified at the phylum level, found in the hindgut of larvae fed different diets and with or without maternal influence treatment. Each bar is the average of the replicates (each bar *n* = 5). At the phylum level, the hindgut is dominated by Firmicutes and Actinobacteriota, with a lesser abundance of Proteobacteria and other phyla. (B) Relative abundance of the top 40 abundant bacteria (all other taxa labelled as Others), identified at the genus level, found in the hindgut of larvae fed different diets and with or without maternal influence treatment. For taxa that are not classified at the genus level, the lowest taxonomic class is labelled (f = Family, c = Class, and o = Order). Each bar is the average of the replicates (each bar *n* = 5).

At the phylum level, the hindgut was dominated by Bacillota and Actinomycetota, with a lesser abundance of Pseudomonadota (Figure 5a). In particular, microbes from the family Lachnospiraceae are highly abundant (~40%–50%) in all diet groups, followed by Ruminococcaceae and Micrococcaceae (Figure 5b). The abundance of different taxa at the genus level does indeed change between the diet groups. Anaerocolumna is around 30% abundant in SD‐fed and HD‐fed larvae regardless of treatment, yet is at a very low abundance (1%) in GD‐fed larvae (Figure 5b). Tyzzerella is highly abundant in GD‐fed larvae and HD‐fed MIB larvae. Tyzzerella is also an indicator species, or a microbial taxon that is uniquely associated with a particular group, for GD‐fed larvae, regardless of treatment.

Indicator species analysis, which determines taxa driving group‐level differences, of the hindgut microbiome determined distinct taxa that were associated with each larval diet. There were 100 taxa that were indicator species for the GD‐fed larvae, 67 for the HD‐fed larvae and 131 for the SD‐fed larvae (Table 1). Through examining the features that are significantly correlated with each diet group in the NMDS plot based on Bray‐Curtis distances, there was some overlap in taxa that were also indicator species (Table 1). Taxa within the phylum Actinomycetota and Bacillota were significantly correlated as well as indicator species in the hindgut of GD‐fed larvae. Other indicator species for GD‐fed larvae were within the phyla Actinomycetota, Bacillota and Pseudomonadota (Table 2). The indicator species in the hindgut of HD‐fed larvae also were from Actinomycetota and Bacillota, as well as Patescibacteria. The indicator species in the hindgut of SD‐fed larvae were from Actinomycetota, Bacillota and Pseudomonadota. Additionally, taxa that were highly correlated with the hindgut of SD‐fed larvae included *Marvinbryantia*, *Clostridium sensu stricto 1* and *Paeniclostridium*. When investigating core taxa by each diet group, there were 23 taxa in the phyla Bacillota that were present in 100% of all hindguts of SD‐fed larvae, and some were also indicator species (Table 2). There were 24 taxa in Actinomycetota and Pseudomonadota that formed the core microbiome of HD‐fed larvae. There were 15 taxa in Actinomycetota and Bacillota that formed the core microbiome of GD‐fed larvae. See Tables S1 and S2 for a full list of taxa that were indicator species and significantly correlated to larval hindguts in each diet group.

**TABLE 1:** Overlap between indicator species and significantly correlated taxa within the hindgut of larvae in each diet group.

Larval diet	Indicator species (*n*)	Significantly correlated taxa (*n*)	Overlap (n)
SD (Silage Dung)	100	8	8
HD (Hay Dung)	67	21	19
GD (Grass Dung)	131	26	17

**TABLE 2:** Taxonomic identity of indicator species and/or significantly correlated taxa in the larval hindgut by diet group.

Larval diet	Phyla	Families/Genera
GD	Actinomycetota	Mycobacteriaceae^a^, Nocardioidaceae^a^, Propionibacteriaceae^a^, Micrococcaceae^c^, ^d^
	Bacillota	Peptostreptococcaceae^a^ (*Paeniclostridium* ^d^, *Romboutsia* ^d^), Christensenellaceae^a^, Clostridiaceae^a^, Lachnospiraceae^d^ (*Tyzzerella*)^a^, Ruminococcaceae^a^ (*Candidatus Soleaferrea* ^d^), Anaerovoracaceae^a^, Peptoniphilaceae^c^ (*Gallicola* ^c^)
	Pseudomonadota	Rhizobiaceae (*Ensifer*)^a^
HD	Actinomycetota	Microtrichaceae^a^, Eggerthellaceae^a^, Micrococcaceae^a^, ^d^ (*Kocuria* ^d^), Nocardioidaceae^a^, ^d^ (*Aeromicrobium* ^d^), Propionibacteriaceae^c^, ^d^ (*Marinilutecoccus* ^d^)
	Bacillota	Sedimentibacteraceae^a^, Lachnospiraceae^a^, Ruminococcaceae^a^
	Patescibacteria	Saccharimonadaceae^a^
	Pseudomonadota	Rhizobiaceae^d^
SD	Actinomycetota	Dietziaceae^a^, Nocardiaceae^a^, Microbacteriaceae^a^, Streptomycetaceae^a^, Corynebacteriaceae^c^
	Bacillota	Peptostreptococcaceae^c^, ^d^ (*Paeniclostridium* ^b^, ^d^, *Romboutsia* ^d^), Christensenellaceae^c^, Clostridiaceae^c^, ^d^ (*Clostridium sensu stricto 1* ^b^, ^d^), Lachnospiraceae^c^, ^d^ (*Marvinbryantia* ^b^), Oscillospiraceae^c^, Anaerovoracaceae^c^
	Pseudomonadota	Devosiaceae^a^, Sphingomonadaceae^a^

^a^ Indicator Species only.

^b^ Significantly correlated only.

^c^ Both indicator species and significantly correlated.

^d^ Core taxa present in 100% of all hindgut samples by respective diet group.

The hindguts of SD and HD larvae have more similar predicted KEGG Orthology (KOs) functional pathways profiles compared to GD larvae (Figure S4a). Manipulation of the presence of maternal microbes was not a significant driver of different functional pathways. Functional pathway analysis identified several KOs that were differentially abundant between the different diet groups listed in Figure S4, Tables S3 and S4. In contrast to HD larvae, GD‐fed larvae were enriched in genes involved in secondary metabolite biosynthesis, lipid metabolism and carbohydrate utilization. SD‐fed larvae were depleted relative to GD‐fed larvae in KOs involved in nutrient transport.

### 3.3 Sources Contributing to the Microbial Community of Larval Hindguts

**FIGURE 6:**
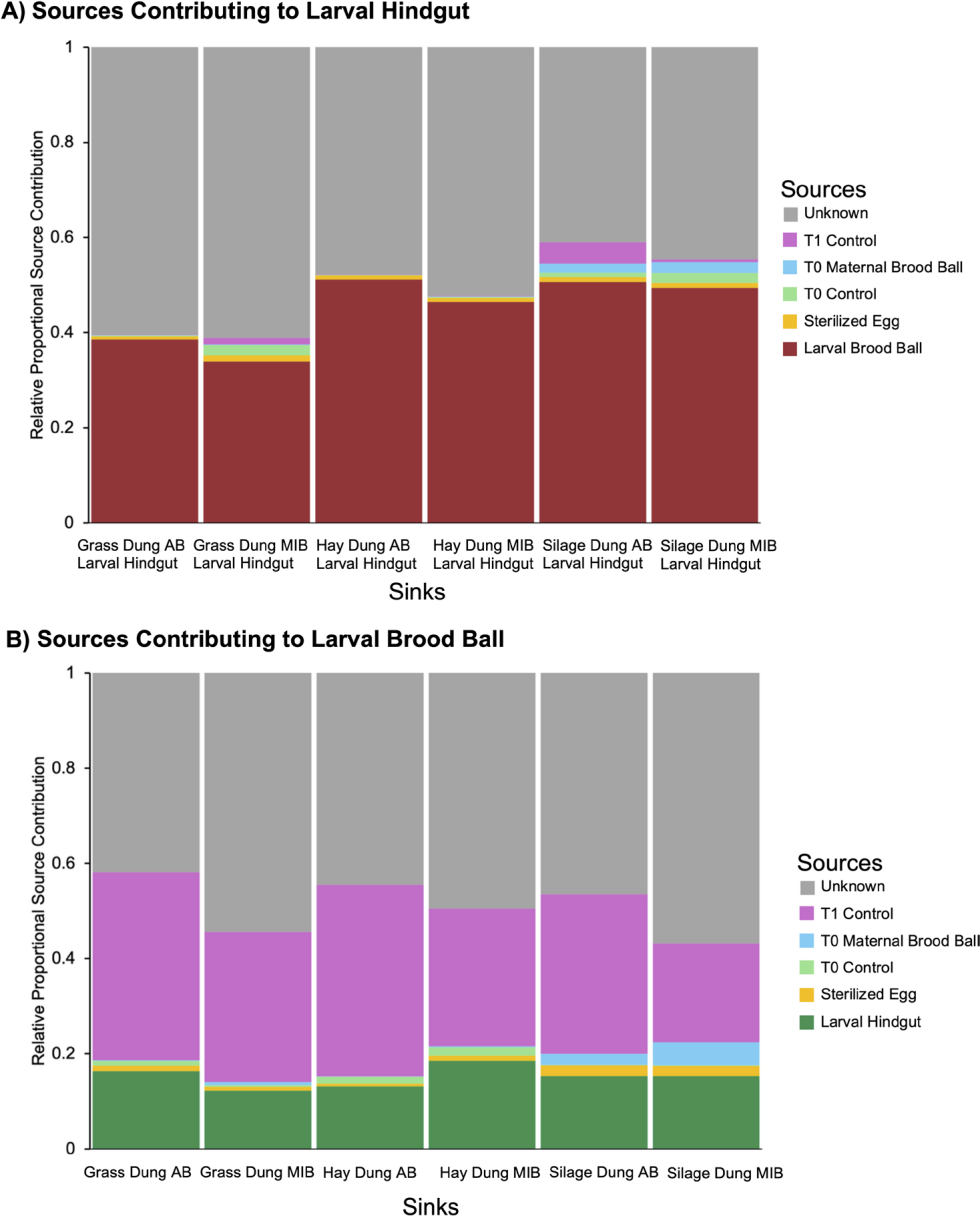
Estimates of relative proportional contributions of bacteria to pre‐determined sinks from pre‐determined sources were made using SourceTracker, a Bayesian method for estimating the proportional contributions of given sources at given sinks. (A) Sources contributing to the larval hindgut samples for each respective diet and maternal treatment groups. The brood ball contributes 40%–50% to the larval hindgut microbiome. (B) Sources contributing to the larval brood ball, mostly the larval hindgut, which contributes ~20% and the T1 Control, which contributes ~40%.

Overall, the brood ball had the highest relative proportional contribution (~40%–50%) of microbes to the hindgut, with the proportion slightly decreasing in the presence of maternal microbes within each diet group (Figure 6a). The T1 Control was the biggest contributor to the brood ball microbiome (~40%), suggesting that the microbial community shifts that occur naturally over time without the larva's impact are a major factor in shaping the brood ball microbiome (Figure 6b). The larval hindgut contributed ~20% of the microbial community, highlighting the reciprocal relationship between larvae and its brood ball environments. In contrast to the hypothesis that vertically inherited microbes play a major role in shaping the larval microbiome, the contribution of vertically transmitted microbes to the hindgut microbiome was very small. However, a proportion of unknown contributed to the larval hindguts and larval brood balls, likely because we did not capture all potential environmental or dietary sources in our experiment. Additionally, the large proportion of unknown could be due to unique taxa in the hindgut, particularly from the phyla Bacillota and Actinomycetota (Figures S5–S7). Patterns inferred from SourceTracker were consistent with ASV‐level overlap visualized in the Venn Diagrams, which highlight limited and diet‐dependent sharing of ASVs between maternal brood balls and larval hindguts, with overlap remaining small relative to total hindgut diversity. There were no core taxa observed in 100% of hindgut samples, but there were 2 taxa in the Family Lachnospiraceae present in 95% of all hindgut samples, and 23 taxa in the phyla Actinomycetota and Bacillota present in 70% of all hindgut samples.

### 3.4 Effect of Diet and Vertical Transmission on the Brood Ball Microbiome in the Presence and Absence of a Beetle Larva

Neither diet nor the presence of a maternal microbial inoculate affected brood ball Shannon's alpha diversity (ANOVA: Diet *F*_2,26_ = 0.409; *p* = 0.669; Treatment *F*_1,26_ = 0.028; *p* = 0.868) or Chao1 richness (ANOVA: Diet *F*_2,26_ = 0.233; *p* = 0.794; Treatment *F*_1,26_ = 0.456; *p* = 0.506) (Figure S8). However, both manipulations affected community composition. Diet explained 24% (PERMANOVA, *R* ^2^ = 0.24, *p* = 0.001) of the variation in the larval brood ball microbial community, while treatment explained ~4% (PERMANOVA, *R* ^2^ = 0.04, *p* = 0.059; Figure 4).

When we compared all brood ball types to each other, sample type was significantly driving the differences among all brood balls (PERMANOVA, *R* ^2^ = 0.15, *p* = 0.001; Figure 4). The microbial communities without a larvae sampled at the beginning of the experiment (T0 control) were clearly distinct from those sampled at the end of the experiment (T1 control), indicating temporal variation in microbial community composition (pairwise PERMANOVA, *R* ^2^ = 0.13, *p* = 0.002). Interestingly, the microbiomes of brood balls that were left empty for 10 days clustered with the brood balls that were inhabited by a larva, yet they were still significantly different (pairwise PERMANOVA, *R* ^2^ = 0.05, *p* = 0.002). This suggests that most of the changes accrued over the course of 10 days do so even in the absence of the beetle larva.

Relative to the GD T1 Control, the GD larval brood balls had taxa that were differentially abundant (ANCOM‐BC), particularly from the Families Lachnospiraceae and Peptostreptococcaceae among other taxa in Bacillota, Actinomycetota and Pseudomonadota. Similar taxa were enriched in HD larval brood balls compared to the HD control. However, these taxa were depleted in SD larval brood balls compared to the SD T1 Control. When comparing the T1 Control to the larval brood balls of larvae fed different diets, the larval brood balls had significantly different predicted KEGG pathways compared to the T1 Controls (PERMANOVA, *R* ^2^ = 0.19, *p* = 0.003; Figure S9).

## 4 Discussion

In this study, we examined how dietary changes and the vertical inheritance of microbes shape the larval gut morphology and microbial communities in *D. gazella*. We found that diet plays a dominant role in shaping the larval hindgut microbial diversity and composition, more so than the presence of maternal microbes. Diet also strongly affected larval size and the relative size of different gut sections while maternal microbes had a small effect on the relative size of the hindgut. Our data suggest that maternally inherited microbes contribute little to the larval gut, which is primarily shaped by the larval brood ball environment, although unique taxa occur in the gut. Larvae alter the microbial community of their brood ball, which highlights the host, gut microbe and environment interactions during development. Our findings emphasize how associations with microbial communities are especially important for animals relying on symbionts to digest compositionally variable diets. This study provides novel insights into the role of microbial inheritance and environmental microbes in shaping host gut community dynamics.

### 4.1 Gut Morphology Reflects Influences of Diet and Maternal Effects

Digestive tract morphology is plastic in response to nutrient composition in many animals (Karasov and Douglas 2013). For instance, in adult dung beetles, diet has been shown to influence hindgut morphology and bacterial communities in a species‐specific manner, highlighting the ability of dung beetles to utilize different digestive strategies (Ebert et al. 2021). Similar responses have been shown in dung beetle larvae, where diet influences gut morphology as well as gut retention time (Rohner and Moczek 2021). In our study, diet was the dominant factor influencing larval growth and the relative size of all three gut sections, suggesting that larval gut morphology is developmentally plastic. This plasticity may reflect adaptive responses to the chemical and microbial differences among dung types, particularly when encountering novel diets such as silage dung. While some dung beetle species are very specialized, most dung beetles commonly use dung of several herbivorous species and demonstrate dietary plasticity. For instance, dung beetles in the subfamily Aphodiinae have been shown to prefer introduced alpaca dung over local cattle dung in the European Alps, which highlights dung beetles' flexibility in responding to variation in dung quality (Rolando et al. 2024). The plastic responses seen in larval gut development might thus represent adaptive plasticity at the larval stage.

In our study, since GD‐fed larvae had larger body sizes than HD‐fed or SD‐fed larvae, the grass dung may be of higher quality or more easily digestible for the larvae. Diet can influence the area of the different sections of the gut, especially the hindgut, to support the digestive demands of the diet. Only in some cases did the presence of maternal microbes influence larval gut morphology. Hindgut area increased when larvae developed in maternally influenced brood balls, possibly indicating a higher fermentative capacity. Interestingly, the presence of maternal microbes, on average, increased overall larval body size but overall, vertically transmitted microbes play a relatively small role in hindgut development and the mechanisms behind this plasticity remain to be investigated. Thus, while vertical transmission explained little overall microbial compositional variance (3%, PERMANOVA n.s.), it influenced microbial richness and certain host traits. Notably, these effects on body size and survival indicate that vertically transmitted microbes can exert biologically meaningful effects on host fitness even when they comprise only a small fraction of the overall gut microbiome.

### 4.2 Diet as a Driver of Microbiome Composition and Potential Multifunctional Redundancy

In contrast to the hypothesis that silage dung would be the most nutritionally challenging diet, SD‐fed larvae had the most diverse microbial communities, as indicated by significantly higher alpha diversity compared to the other diet groups. Possibly the more nutrient‐rich and less fibrous silage dung diet supports a more diverse microbial community, offering more metabolic niches compared to more fibrous grass and hay dung diets. Cow dung is typically rich in cellulose and relatively poor in nitrogen, with a high Carbon to Nitrogen ratio (Hindrichsen et al. 2006). Yet, cow dung differs in nutrient composition and fermentative profiles based on the diet of the cow. For instance, a maize silage diet is rich in starch and has distinct microbial and chemical properties compared to the higher fibre hay and grass diets (Hindrichsen et al. 2006). These differences in the dung based on the diet of the cow certainly can influence substrate availability and shape the gut microbiome of the dung‐feeding larvae.

Diet has been found to be a driver of the gut microbiome of insects and other adult dung beetle species, with evidence of diet‐dependent shifts in microbiome composition (Shukla et al. 2016; Colman et al. 2012; Franzini et al. 2016). Further, the influence of the pedestal (or ‘maternal gift’) has been documented in other dung beetle species, such as *Ontophagus taurus*, and larvae share identical microbial taxa with the mother (Estes et al. 2013). In our study, the presence of maternal microbes did influence microbial richness, especially within SD‐fed larvae. SD‐fed larvae with access to a maternal inoculate had the highest microbial richness compared to the SD‐fed larvae without the inoculate and other larvae, suggesting that microbial inoculation does enrich microbial communities within silage dung.

Microbial treatment alone did not significantly shape overall community composition when examining beta diversity, and diet exerted a stronger effect on microbiome assembly. The larval hindgut communities clustered distinctly by diet and explained ~26% of the variation in microbial community structure, indicating compositional shifts across different dung types. Anaerocolumna, a fermentative cellulose degrader that produces the short‐chain fatty acid acetate, was abundant in SD‐fed and HD‐fed larvae, but nearly absent in GD‐fed larvae (Ueki et al. 2019). This suggests microbes are playing an important role in breaking down plant substrates in SD‐fed and HD‐fed larvae. On the other hand, Tyzzerella was more prevalent in GD‐fed larvae and HD‐fed MIB larvae. Tyzzerella is in the Family Lachnospiraceae, which is associated with fermentation and short‐chain fatty acid production. Tyzzerella has also been found as a core microbe in other dung beetle species, such as in the genus *Cephalodesmius* (Ebert et al. 2021). Thus, while the specific microbes present vary by diet, they largely perform similar fermentative functions.

We predicted that different diets may impact the function of the microbes in the hindgut of dung beetle larvae. SD‐fed larvae exhibited typical fermenting microbes that were both indicator species, core taxa and significantly correlated with the SD‐fed larvae including Lachnospiraceae (*Marvinbryantia*), Clostridiaceae (*Clostridium sensu stricto 1*) and Peptostreptococcaceae (*Paeniclostridium*). These taxa are associated with fermentative gut environments and short‐chain fatty acid production, with *Marvinbryantia* producing acetate and *Clostridium sensu stricto 1* producing butyrate, suggesting silage dung fostered a hindgut community that is more tailored towards metabolizing complex carbohydrates and amino acids to enhance digestive efficiency. Additionally, Peptostreptococcaceae is associated with protein digestion and found in animals with lower protein diets, and this taxa may aid larvae in amino acid scavenging from the low protein silage dung (Fan et al. 2017; Costello et al. 2010; Lemieux‐Labonté et al. 2022). In contrast, these taxa were not found as indicator species or significantly correlated with the hindgut of either the HD‐fed or GD‐fed larvae. Romboutsia was found in both SD‐fed and GD‐fed larvae as a core taxon and is associated with carbohydrate fermentation (Gerritsen et al. 2014). The GD‐fed larvae were marked by Actinomycetota (Micrococcaceae) and Bacillota (*Gallicola*), of which *Gallicola* is an obligate anaerobe that does not utilize carbohydrates for fermentation, but metabolizes peptone and amino acids to acetic and butyric acid (Ezaki 2015). Taxa that were indicator species, formed the core microbiome and significantly correlated to HD‐fed larvae were from other families within Actinomycetota, including Propionibacteriaceae, which produces propionic acid through fermentation (Turgay et al. 2022). Additionally, Rhizobiaceae, which was a core taxa present in 100% of the HD‐fed larvae may indicate nitrogen fixation in the gut, which has been found in termites and bark beetles (Morales‐Jiménez et al. 2013) to mediate nitrogen requirements due to the high C:N dung diet (Nardi et al. 2002; Alonso‐Pernas et al. 2017; Bar‐Shmuel et al. 2020). Additionally, there have been associations with *Euoniticellus* dung beetles and nitrogen supplementing bacteria in larvae that they obtained through maternal transmission or from the dung (Shukla et al. 2016).

Overall, these findings suggest that while all three diets supported microbial fermentation to varying degrees, contrary to our expectations, SD‐fed larvae had the most typical fermentative gut profile, harbouring taxa involved in butyrate‐producing Bacillota. HD‐fed larvae relied more on microbes within Actinomycetota that are involved in propionate‐producing pathways, whereas GD‐fed larvae had a microbial community that includes *Gallicola*, which utilizes amino acid metabolism. Thus, although there are some taxonomic differences due to the different diets, the functionality of the microbiota as fermenters remains the same, supporting the concept of multifunctional redundancy in the gut ecosystem (Moya and Ferrer 2016). This functional consistency, even across novel dung types such as silage, highlights the resilience of the association between dung beetle larvae and their microbiome to changes in resource composition.

Predicted functional profiles based on KEGG Orthology suggest that the larval microbiomes from SD‐fed and HD‐fed larvae are functionally more similar to each other compared to GD‐fed larvae, although there were no pathways involved in fermentation that were significantly enriched or depleted among different diet groups (Figure S4). Inspection of predicted KEGG pathways indicated that genes associated with acetate, butyrate and propionate metabolism were likely present across hindgut samples, consistent with shared fermentative potential rather than diet‐specific functional shifts (Table S5, Figures S10–S12). In HD‐fed larvae, enriched KO pathways included stress response and membrane transport functions, potentially reflecting microbial adaptation to the more recalcitrant and fibrous hay dung. SD‐fed larvae also exhibited enrichment in pathways related to stress response, which may indicate microbial responses to the chemical and nutritional novelty of silage dung. In contrast, GD‐fed larvae were enriched in genes involved in nutrient transport and secondary metabolism, suggesting a microbiome more suited for efficient nutrient acquisition. While functional predictions provide useful insights, they should be viewed as hypotheses of potential function, since they are inferred from 16S rRNA data in PICRUST2 and depend on reference genomes, requiring further validation with functional assays or metagenomic data. In addition, it is important to highlight that the dung used in the experiments was previously frozen to remove insect larvae and parasites and that we cannot exclude that freezing altered the functional profiles. However, this treatment was applied uniformly across all dung types and therefore cannot explain the observed differences among them. Overall, the microbiome results of the larval hindgut support the expectation that diet is a primary ecological driver shaping gut microbial diversity and communities, and the hindgut serves a role as a fermentation chamber for the host by breaking down complex carbohydrates.

### 4.3 Brood Ball Contributes Most to the Hindgut Microbiome

Our findings indicate that the brood ball is the primary microbial source for the larval hindgut microbiome, contributing 40%–50% of the community. This suggests that larvae acquire most of their gut microbes through ingestion of the brood ball environment. The larvae of other species of dung beetles, including *Onthophagus taurus* and *Catharsius molossus*, also acquire microbes from their brood ball environment, with the microbiota composition changing as larvae develop and consume their own excrement (Estes et al. 2013; Chen et al. 2024).

Despite the brood ball's dominant contribution, there was still a large proportion of an ‘unknown’ source. This may be explained by unique taxa in the hindgut, predominantly from the phyla Bacillota and Actinomycetota, which may represent gut‐specific taxa that change once established in the host. This highlights the complexity of microbial transmission and suggests that the gut is a selective environment that can shape a distinctive microbiome. This is consistent with findings from other insect systems, where the gut serves as a selective filter to maintain host‐specific microbes. For instance, the gut environment of the bean bug *Riptortus pedestris* selectively supports and retains only specific symbiont strains through microbial competition (Itoh et al. 2019). In bumblebees, there are conserved gut communities that persist across individuals and species that reflect host‐microbe coevolution and selection by the gut (Kwong and Moran 2015). In this realm, while the brood ball was the key source of the larval hindgut microbial communities, the larval gut plays an active role in shaping and maintaining a specialized microbiome.

Despite a high number of taxa unique to the hindgut, there were no taxa that formed the core microbiome at 100% prevalence in all individuals across different diets and treatments. The lack of core taxa is not unexpected, given inter‐individual variation and the influence of diet on microbial communities. However, the detection of two taxa in 95% of hindgut samples in the Family Lachnospiraceae highlights the presence of fermenting microbes in the larval hindgut.

### 4.4 The Larvae Actively Changed the Microbiome Composition of the Brood Ball Environment

Dung beetle larvae have been documented to manipulate their brood ball throughout development in ways that benefit their own growth and survival (Schwab et al. 2016; Schwab et al. 2017). This effect is thought to be driven by changes in the microbial community in the brood ball (Schwab et al. 2017). Our findings indicate that there are indeed large shifts in community composition over time, but there are shifts in microbial composition regardless of the presence of larvae. These temporal dynamics were relatively consistent across the three dung types, suggesting a common pattern of microbial turnover. However, the presence of a larva did have a significant effect on the microbial community. In artificial brood balls that inhabited larvae, there was a differential abundance of taxa, including Bacillota, involved in fermentation that were depleted in the controls compared to HD and GD larval brood balls respectively. Further, the functional pathways present in brood balls seem to have a larger variation and range compared to the T1 Controls (Figure S9). Additionally, the larval hindgut contributed ~20% to the larval brood ball community, while the T1 Control contributed the largest proportion (Figure 6b). This pattern garners some support for the external rumen hypothesis, where larvae actively engineer the brood ball microbiome to facilitate microbial fermentation and nutrient acquisition outside the gut, ultimately promoting growth and development (Halffter 1997; Holter 2016; McConnell and Rohner 2025).

## 5 Conclusions

Our findings show that larval diet is a primary driver that shapes the hindgut microbiome diversity and composition, while the presence of maternal microbes plays a comparatively minor role. The brood ball contributes almost half to the composition of the hindgut, yet the hindgut itself also harbours unique taxa, suggesting selective pressures within the gut environment. Larvae not only acquire microbes from their environment but also actively alter brood ball microbial communities. These findings highlight the reciprocal interactions between larvae and their microbial environments during development.

These results suggest that the dung beetle larval gut microbiome is structured less by vertical transmission from the mother, and more by dietary and environmental inputs, with variation in fermentative taxa across dung types differing in composition and novelty. This flexibility may be critical for dung beetles as they encounter compositionally novel resources, such as silage dung, highlighting the role of microbiome‐mediated plasticity in adapting to changing agricultural landscapes. Further, this work highlights how the interplay between diet, environmental microbes and limited maternal inheritance can shape gut communities in ways that influence host gut development and digestion. These insights contribute to our understanding of how symbioses mediate ecological resilience, informing predictions of how insects and microbes respond to shifts in resource availability changes.

## Author Contributions

Michelle J. Herrera conducted animal husbandry, tissue harvesting, molecular analyses, statistical analyses and study design. Avi Khanna was involved in study design, animal husbandry, gut segment and body size measurements and analysis and statistical analyses. Joshua A. Jones was involved in study design, dung processing and consulting on data analyses. Ricardo Betancur‐R. was involved in consulting on data analysis. Patrick T. Rohner was involved in study design, animal husbandry, tissue harvesting, molecular analyses and statistical analyses. All authors contributed to the writing of this manuscript.

## Funding

This work was supported by the Department of Ecology, Behaviour, and Evolution and the School of Biological Sciences at the University of California San Diego (to Patrick T. Rohner) and the University of California, San Diego Chancellors Postdoctoral Fellowship (to Michelle J. Herrera).

## Ethics Statement

The authors have nothing to report.

## Conflicts of Interest

The authors declare no conflicts of interest.

## Supporting information


**Figure S1:** Linear length for the body was measured via spiracle distance, which was correlated with individuals' body area (*R* = 0.81, *p* = 5.3e‐16).
**Figure S2:** Alpha rarefaction curves (based on observed features) for samples included in this study.
**Figure S3:** Residual total gut size, foregut size and midgut size of larvae. Error bars indicate 95% confidence intervals.
**Figure S4:** (A) Principal Coordinates Analysis (PCA) of beta diversity based on bray–curtis dissimilarity in KEGG Orthology (KO) functions of the larval hindgut.
**Figure S5:** Venn Diagram of Silage AB and MIB treatment.
**Figure S6:** Venn Diagram of Hay AB and MIB treatment.
**Figure S7:** Venn Diagram of Grass AB and MIB treatment.
**Figure S8:** Neither diet nor the presence of a maternal microbial inoculate affected the Shannon's alpha diversity (ANOVA: Diet *F*_*2,26*_ = 0.409; *p* = 0.669; Treatment *F*_*1,26*_ = 0.028; *p* = 0.868) or Chao1 richness (ANOVA: Diet *F*_*2,26*_ = 0.233; *p* = 0.794; Treatment *F*_*1,26*_ = 0.456; *p* = 0.506) in brood balls.
**Figure S9:** Principal coordinates analysis (PCA) of beta diversity based on bray–curtis dissimilarity in KEGG Orthology (KO) functions of all larval broodballs versus T1 Controls.
**Figure S10:** KEGG pathway map illustrating acetate production within pyruvate metabolism.
**Figure S11:** KEGG pathway map illustrating butyrate production within butanoate metabolism.
**Figure S12:** KEGG pathway map illustrating propionate production within propanoate metabolism.
**Table S1:** Full list of Indicator Species in all diet groups.
**Table S2:** Full list of taxa that are highly correlated with each respective diet group.
**Table S3:** Kegg Orthologs (KOs) significantly enriched or depleted in the hindgut microbiomes of hay dung fed larvae compared to grass dung fed larvae.
**Table S4:** Kegg Orthologs (KOs) significantly enriched or depleted in the hindgut microbiomes of silage dung fed larvae compared to grass dung fed larvae.
**Table S5:**. Predicted KEGG pathways related to fermentation in the larval hindgut.

## Data Availability

The datasets presented in this study can be found in online repositories; accession number for the Bioproject is PRJNA1338251 where the Biosamples and SRA files are located. Scripts and code are located in GitHub repository: https://github.com/mjherre1/D.gazella_microbiome.git.
