# Supplementary material for: Diet Outweighs Vertical Transmission in Shaping Dung Beetle Larval Gut Microbiomes
Source: Mol Ecol. 2026 Apr 3;35(7):e70336. doi: 10.1111/mec.70336 (PMC13047525; doi:10.1111/mec.70336)
Supplement: Supplementary file 1 — Figure S1: Linear length for the body was measured via spiracle distance, which was correlated with individuals' body area (R = 0.81, p = 5.3e‐16). Figure S2: Alpha rarefaction curves (based on observed features) for samples included in this study. Figure S3: Residual total gut size, foregut size and midgut size of larvae. Error bars indicate 95% confidence intervals. Figure S4: (A) Principal Coordinates Analysis (PCA) of beta diversity based on bray–curtis dissimilarity in KEGG Orthology (KO) functions of the larval hindgut. Figure S5: Venn Diagram of Silage AB and MIB treatment. Figure S6: Venn Diagram of Hay AB and MIB treatment. Figure S7: Venn Diagram of Grass AB and MIB treatment. Figure S8: Neither diet nor the presence of a maternal microbial inoculate affected the Shannon's alpha diversity (ANOVA: Diet F 2,26 = 0.409; p = 0.669; Treatment F 1,26 = 0.028; p = 0.868) or Chao1 richness (ANOVA: Diet F 2,26 = 0.233; p = 0.794; Treatment F 1,26 = 0.456; p = 0.506) in brood balls. Figure S9: Principal coordinates analysis (PCA) of beta diversity based on bray–curtis dissimilarity in KEGG Orthology (KO) functions of all larval broodballs versus T1 Controls. Figure S10: KEGG pathway map illustrating acetate production within pyruvate metabolism. Figure S11: KEGG pathway map illustrating butyrate production within butanoate metabolism. Figure S12: KEGG pathway map illustrating propionate production within propanoate metabolism. Table S1: Full list of Indicator Species in all diet groups. Table S2: Full list of taxa that are highly correlated with each respective diet group. Table S3: Kegg Orthologs (KOs) significantly enriched or depleted in the hindgut microbiomes of hay dung fed larvae compared to grass dung fed larvae. Table S4: Kegg Orthologs (KOs) significantly enriched or depleted in the hindgut microbiomes of silage dung fed larvae compared to grass dung fed larvae. Table S5:. Predicted KEGG pathways related to fermentation in the larval hindgut. [file MEC-35-e70336-s001.docx]

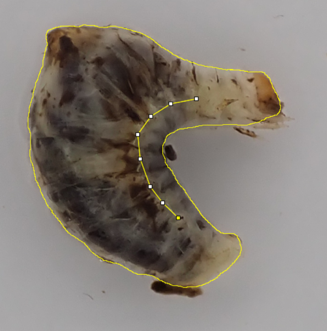

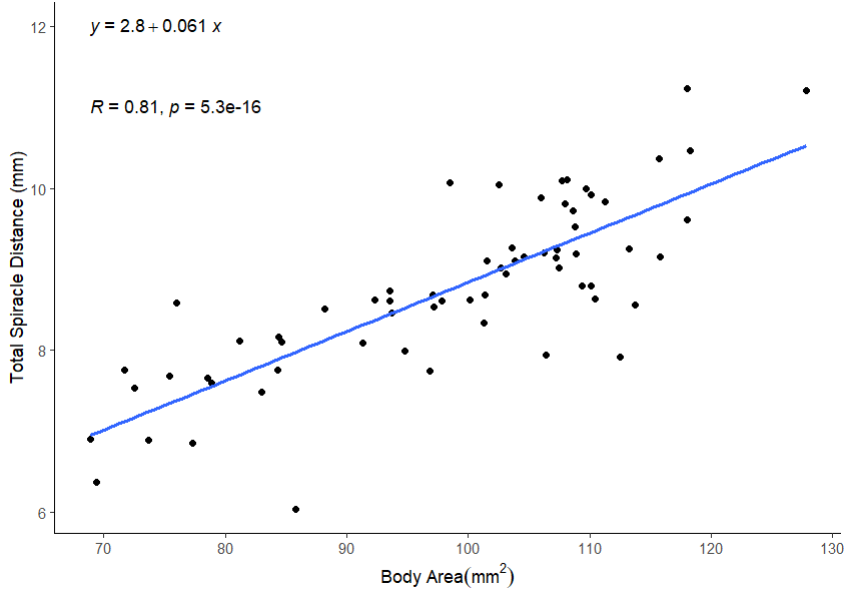


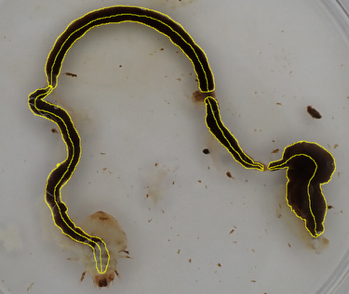


Figure S1. Linear length for the body was measured via spiracle distance, which was correlated with individuals’ body area (R =0.81, P = 5.3e-16). Images show how measurements of body area, gut area, and gut length were measured on ImageJ.


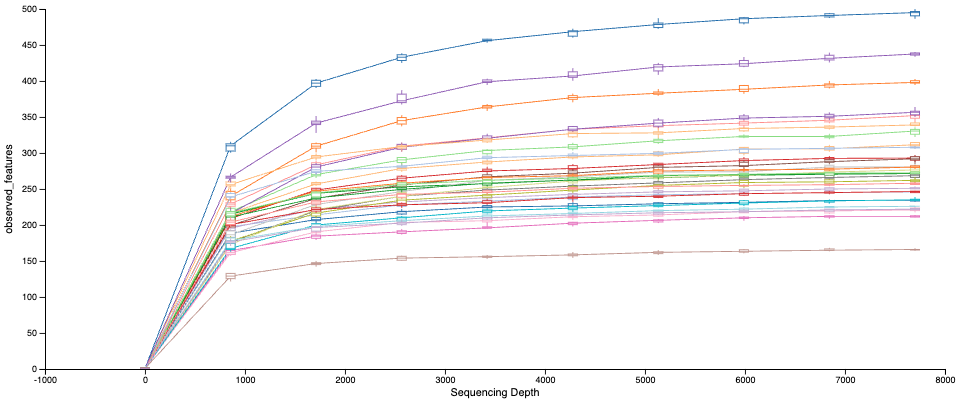


**B) Brood Ball samples**

**A) Hindgut samples**


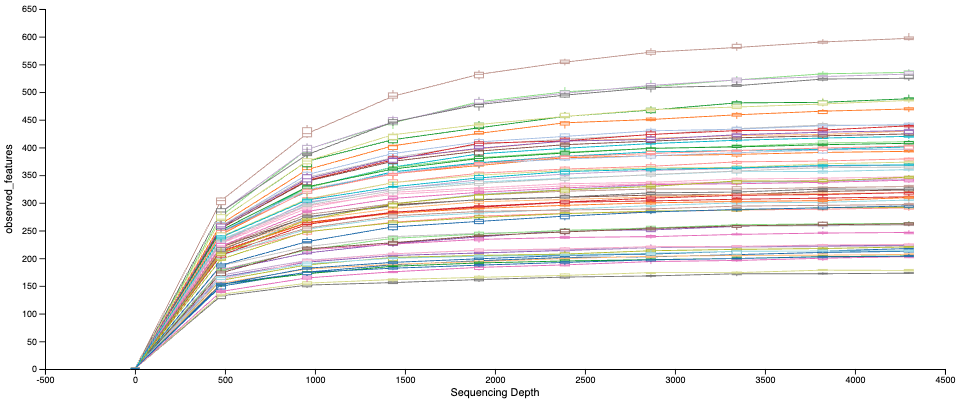


**B) All samples**


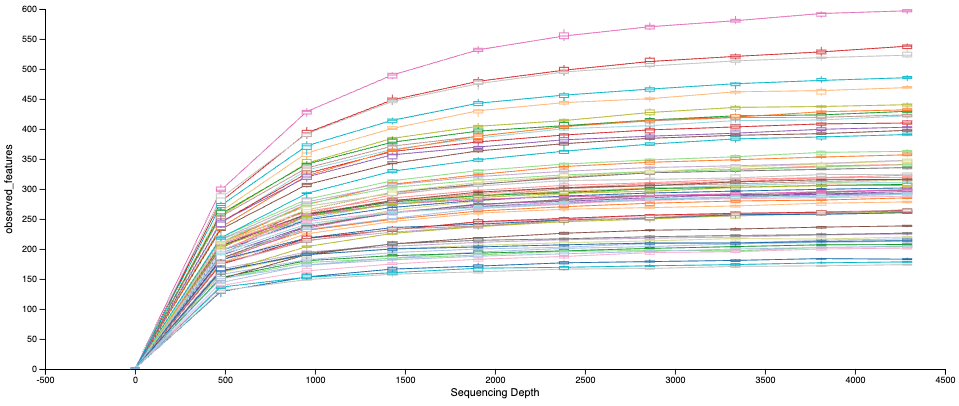


Figure S2. Alpha rarefaction curves (based on observed features) for samples included in this study. Curves depict observed ASV richness for each sample (as depicted by various colors) as a function of sequencing depth and are shown up to the selected rarefaction cutoff used for alpha and beta diversity analyses. Cutoff depths were chosen at points where curves began to level off, ensuring stable diversity estimates while retaining all samples.


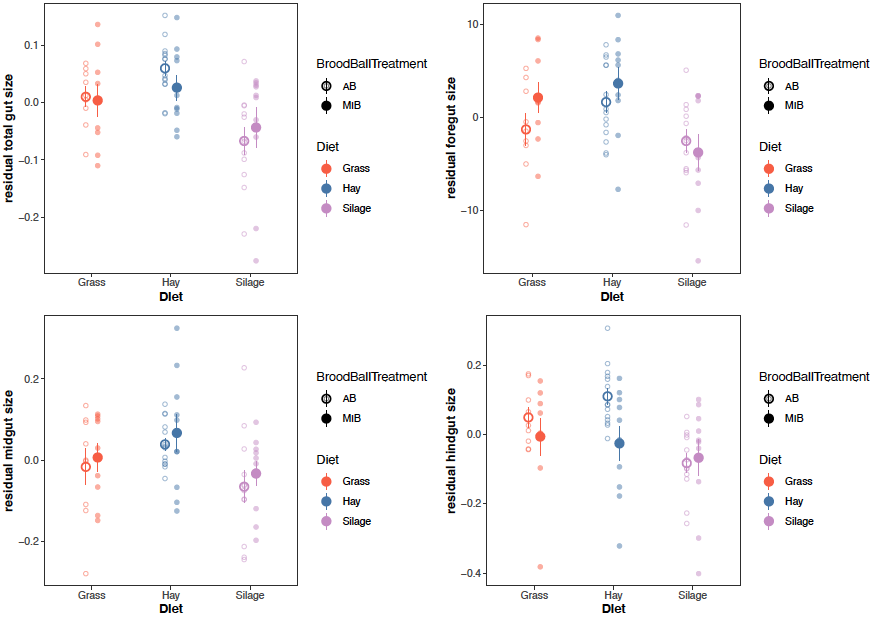

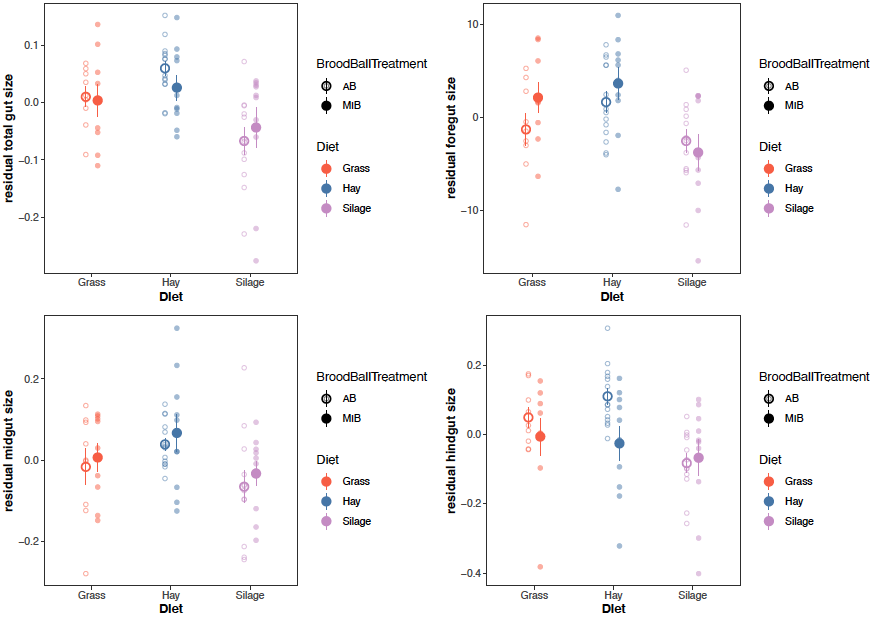


Figure S3. Residual total gut size, foregut size, and midgut size of larvae. Error bars indicate 95% confidence intervals.


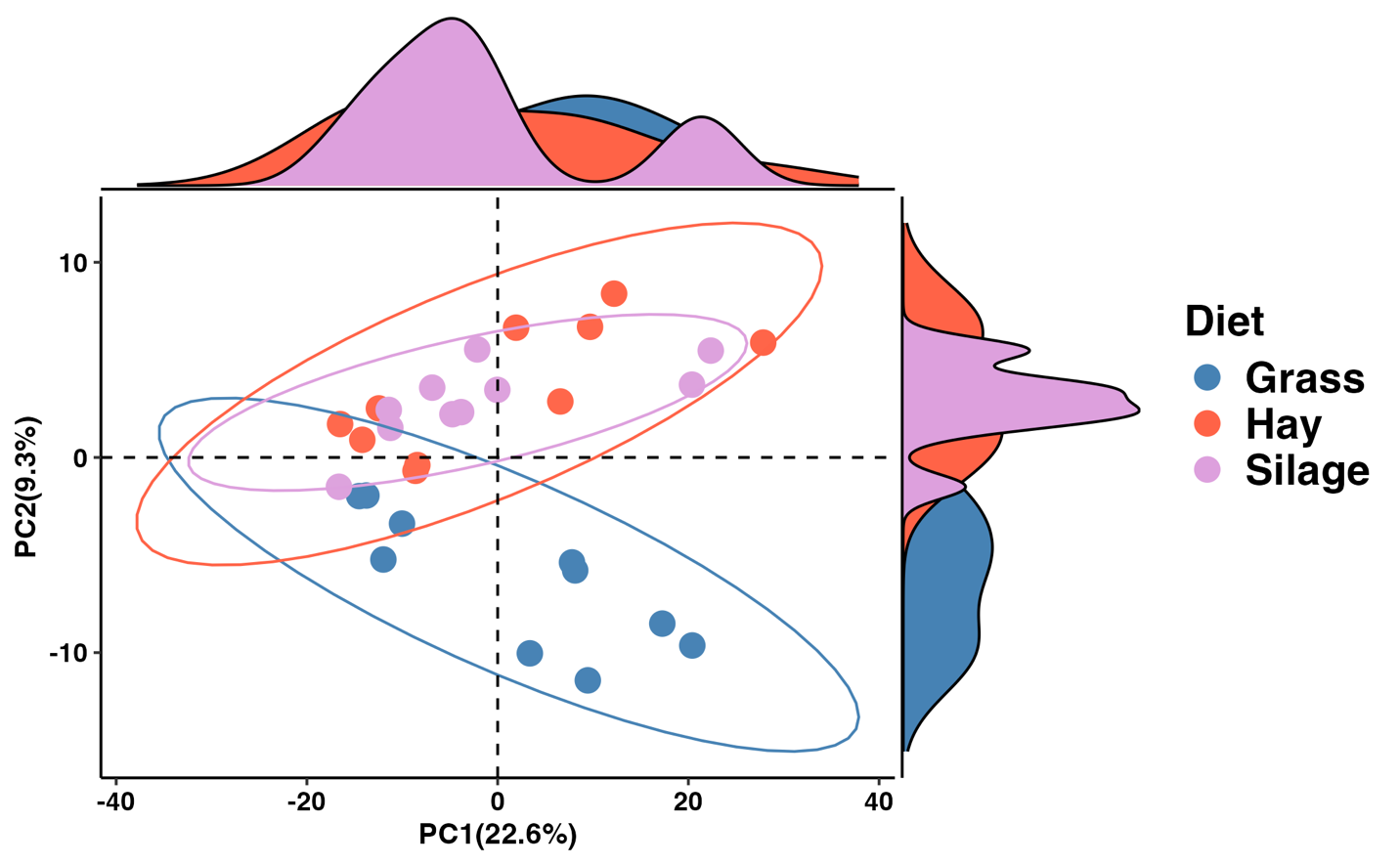


**B)**

**A)**

**
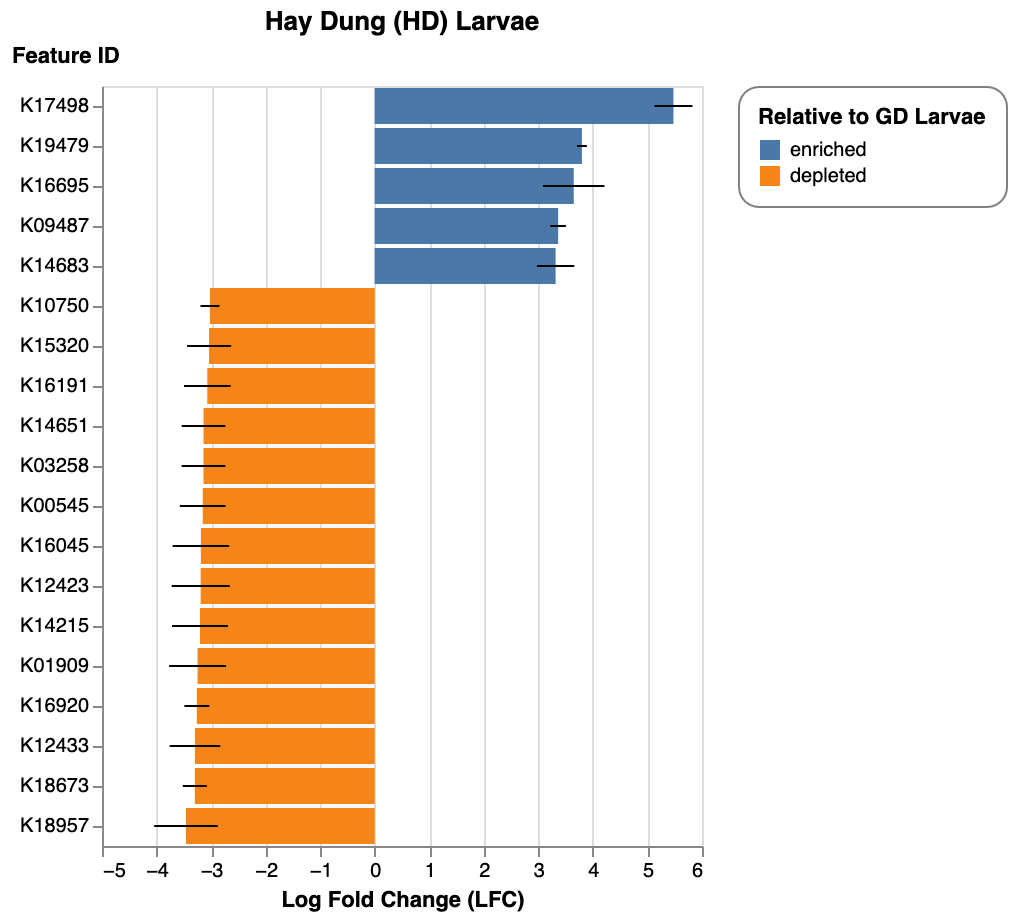
**

**
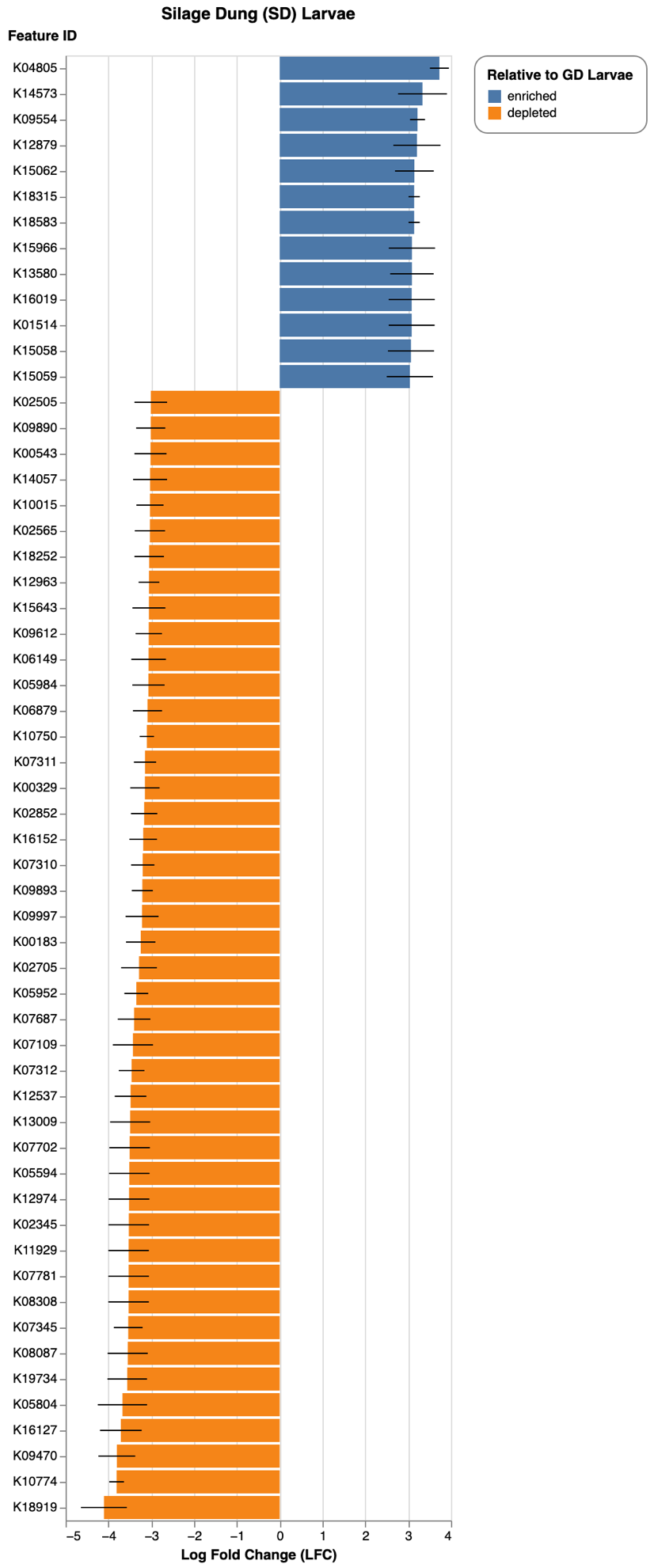
**

**C)**

Figure S4. A) Principal Coordinates Analysis (PCA) of beta diversity based on bray-curtis dissimilarity in KEGG Orthology (KO) functions of the larval hindgut. Each point represents a sample, and the samples that are closer together have more similar KO functional profiles. The different colors represent the different diet groups of Grass (blue), Hay (red), and Silage (purple). B) Differentially abundant KOs in the hindgut microbiome of HD larvae relative to GD larvae. Bars represent log fold change (LFC) values for individual Kos, with positive values indicating enrichment in HD larvae and negative values indicating enrichment in grass fed larvae. Only KOs with an absolute LFC ≥ 3 (i.e., LFC > 3 or < -3) are shown. Error bars reflect the lower and upper bounds of the LFC estimate (± standard error). Bar colors indicate direction of enrichment relative to GD larvae. See Table 3 for full KO annotations and Table S3 for a full list of differentially abundant KOs. C) Differentially abundant KOs in the hindgut microbiome of SD larvae relative to GD larvae. See Table 4 for full KO annotations and Table S4 for a full list of differentially abundant KOs.

For clarity, we focus on KOs with an absolute log fold change (LFC) ≥ 3. See Table S3 for a full list of additional KO IDs with smaller log fold changes that were differentially abundant between HD and GD larvae. Relative to GD larvae, HD larvae had enrichment of genes associated with stress response (heat shock protein HSP90B, K09487), membrane transport (lipopolysaccharide exporter, K16695), transcriptional regulation (SPN1 transcription factor, K17498), and ion transport (sodium-dependent phosphate cotransporter, K14683) (Figure S3b; Tables 3, S3). In contrast to HD larvae, GD-fed larvae were enriched in genes involved in secondary metabolite biosynthesis (polyketide synthase 5, K12433; 6-methylaslicylic acid synthase, K15320), lipid metabolism (fatty acid CoA ligase FadD21, K12423), carbohydrate utilization (beta-glucoside kinase, K18673), and components of transcriptional and translational machinery (TFIID subunit 15, K146511; translation initiation factor 4B, K03258).

Relative to GD larvae, SD larvae were enriched in genes related to stress signaling (nicotinic acetylcholine receptor alpha-3, K04805; CDC37, K09554), transcription and RNA processing (NOP4, K14573; THOC2, K12879), and secondary metabolism (e.g., dioxygenases K15058, K15059; monooxygenase mtmOIV, K15966; carbapenem and carboxymethylproline synthases, K18315, K18583) (Table 4). SD larvae were depleted relative to GD larvae in KOs involved in nutrient transport (histidine transporter, K10015; arginine transport, K09997) amongst other cellular functions. See Table S4 for a full list of additional KO IDs that were differentially abundant between SD and GD larvae.


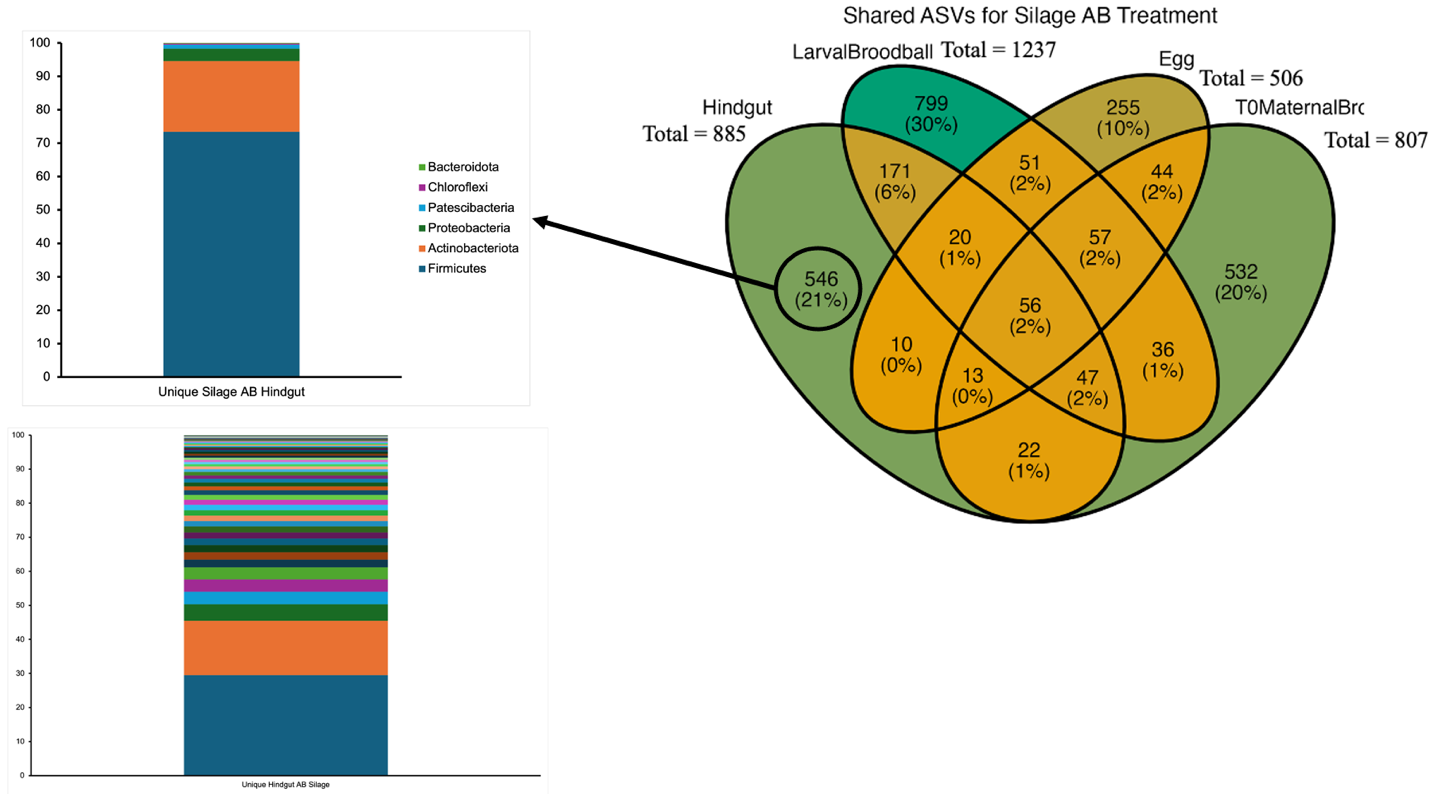
**
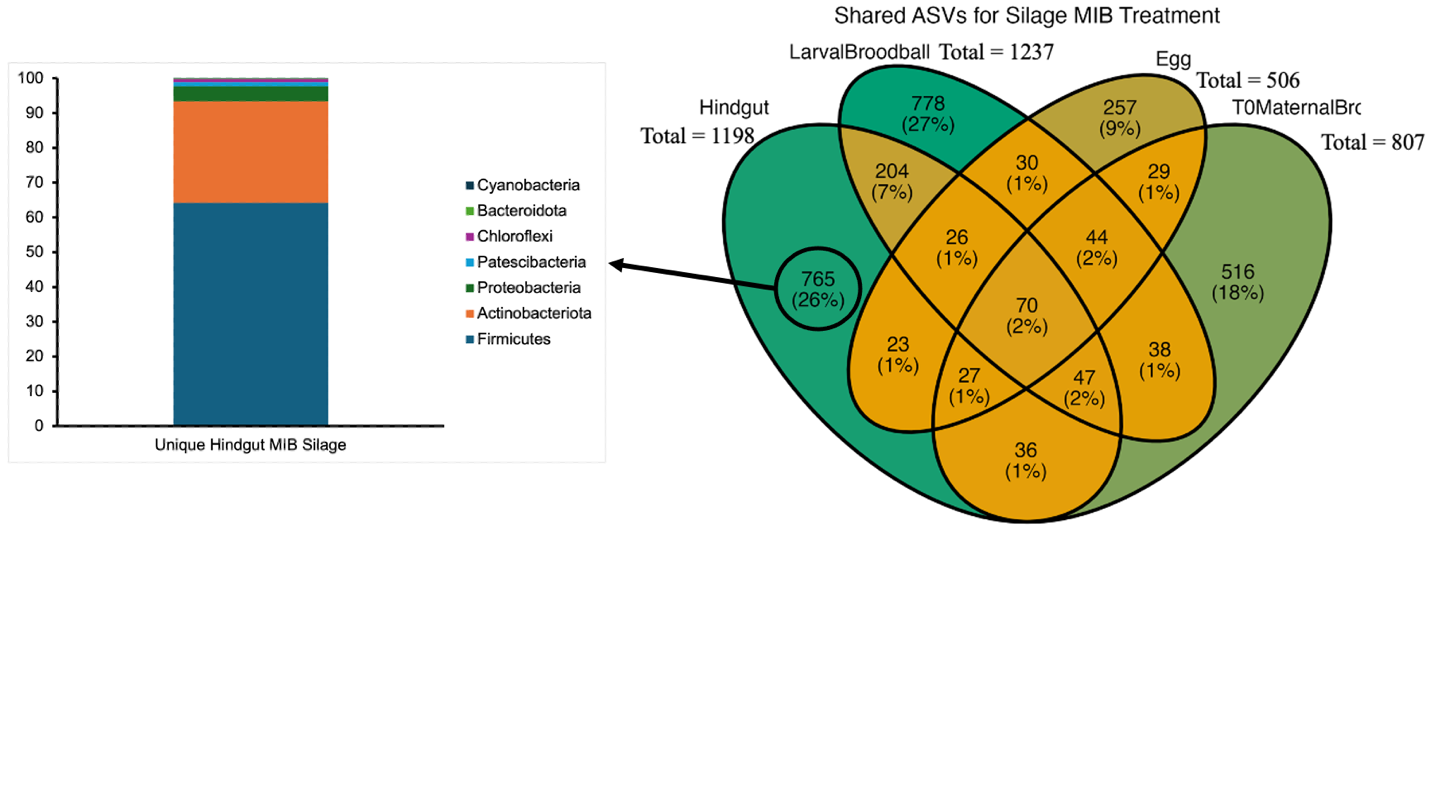
**

Figure S5. Venn Diagram of Silage AB and MIB treatment. Amount of T0Maternal BroodBall shared with Hindgut does increase from 22 ASVs in the AB Treatment to 36 ASVs in MIB treatment. The ASVs that are unique to the hindgut are depicted in the taxa bar plot, and Firmicutes and Actinobacteriota are highly abundant.


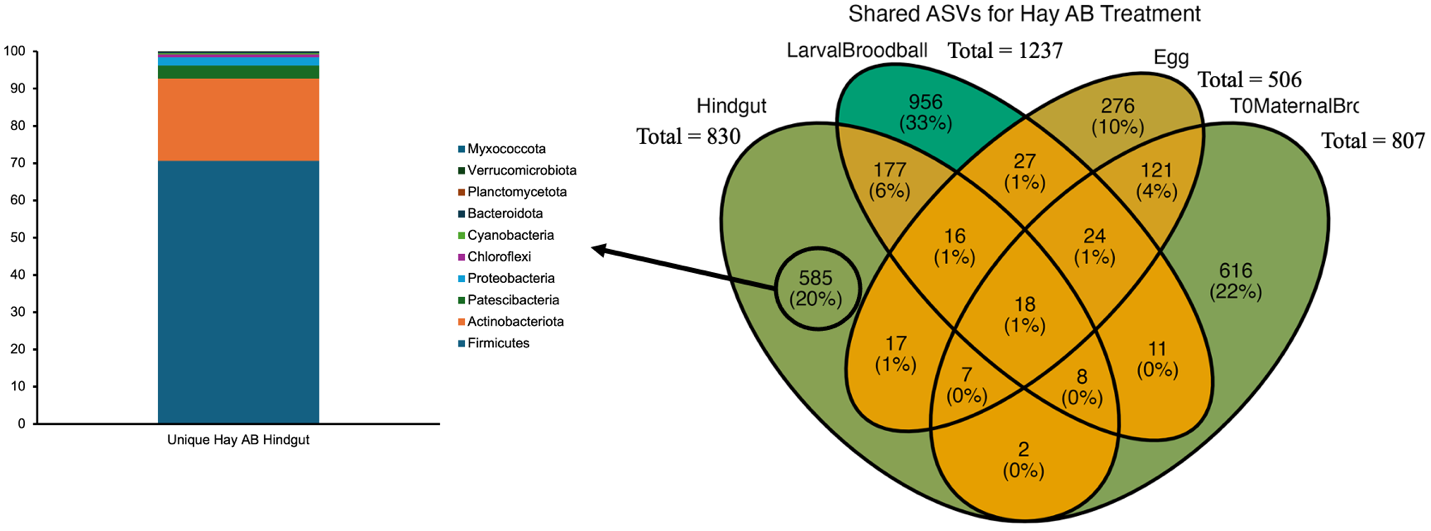

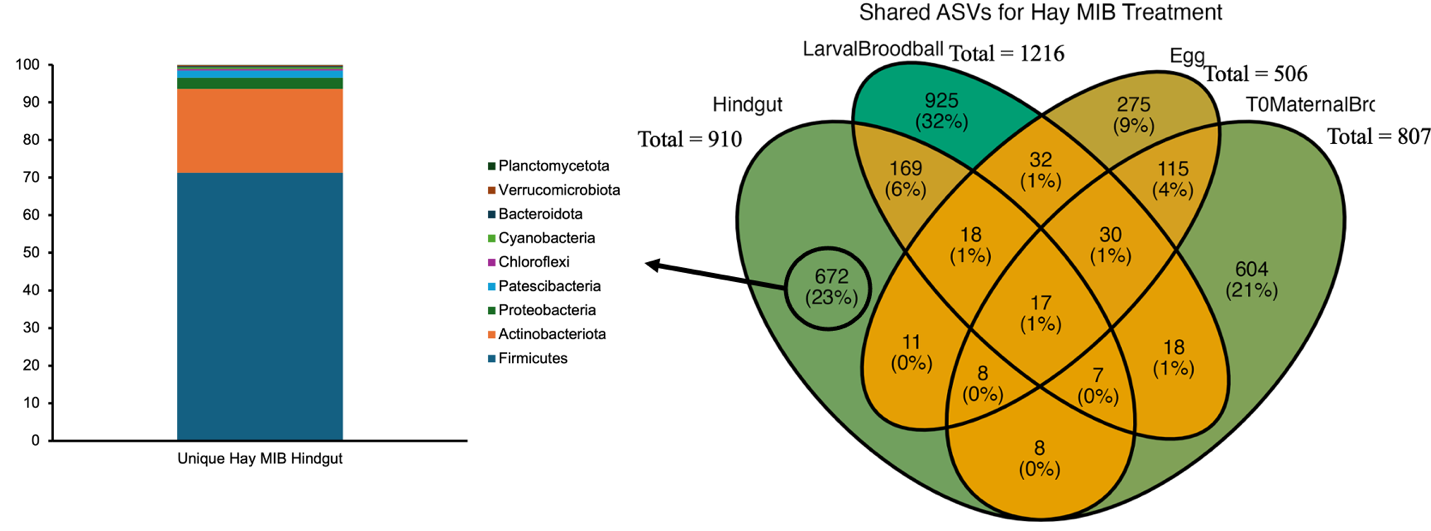


Figure S6. Venn Diagram of Hay AB and MIB treatment. Amount of T0Maternal BroodBall shared with Hindgut does increase from 2 ASVs in AB to 8 ASVs in MIB treatments. The ASVs that are unique to the hindgut are depicted in the taxa bar plot, and Firmicutes and Actinobacteriota are highly abundant.


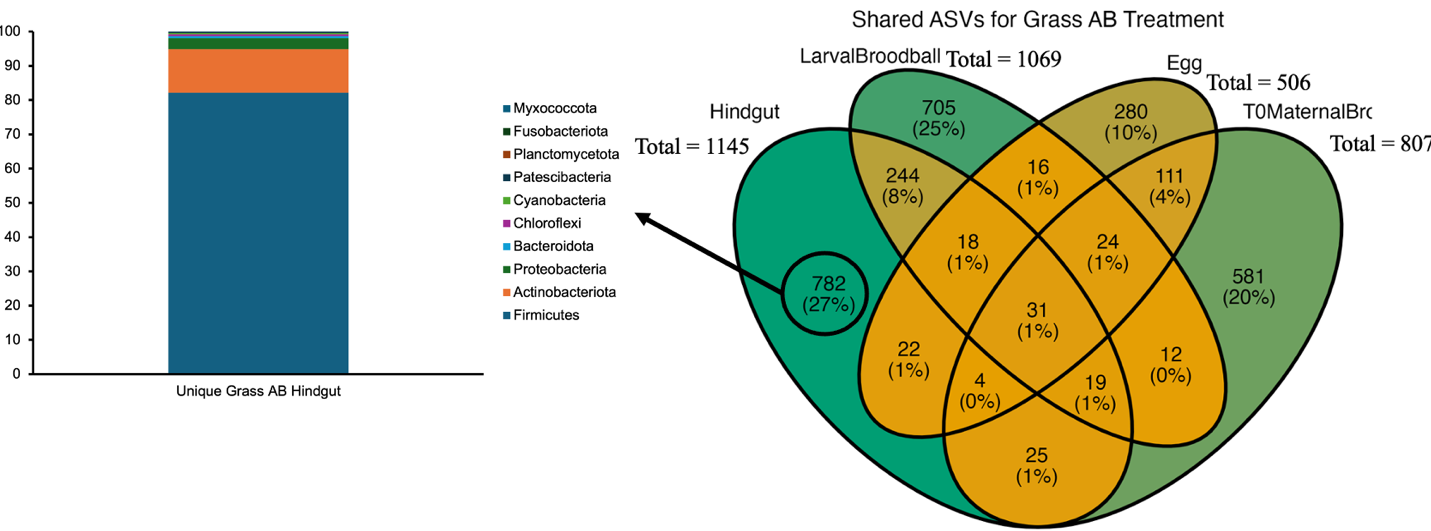

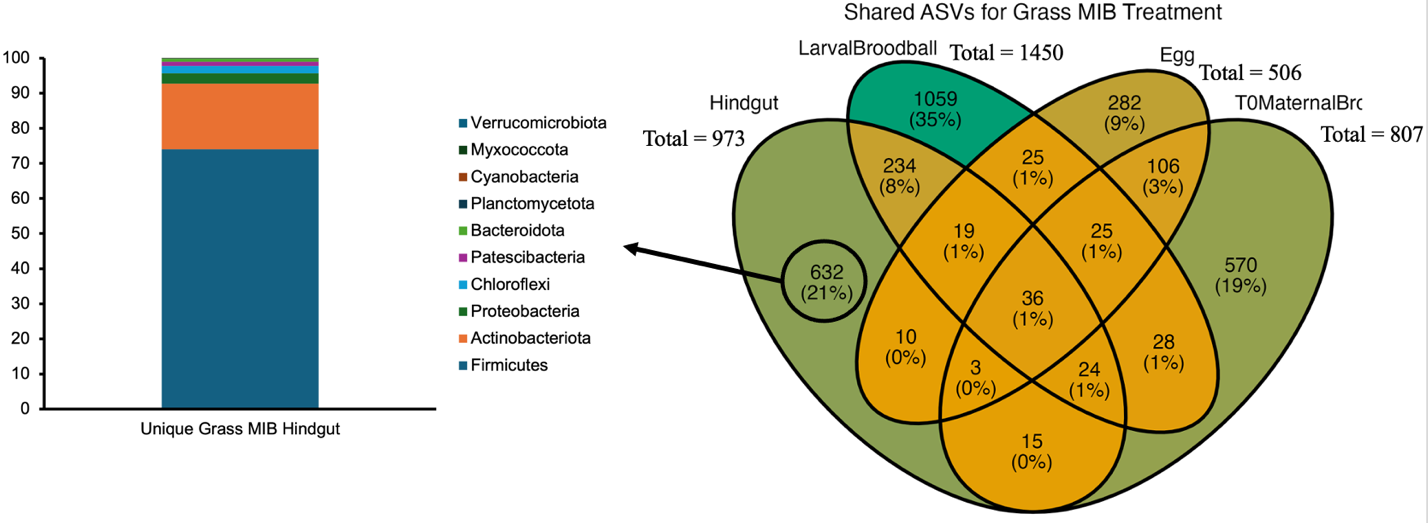


Figure S7. Venn Diagram of Grass AB and MIB treatment. Amount of T0Maternal BroodBall shared with Hindgut decreases from 25 ASVs in AB to 15 ASVs in MIB treatments. The ASVs that are unique to the hindgut are depicted in the taxa bar plot, and Firmicutes and Actinobacteriota are highly abundant.


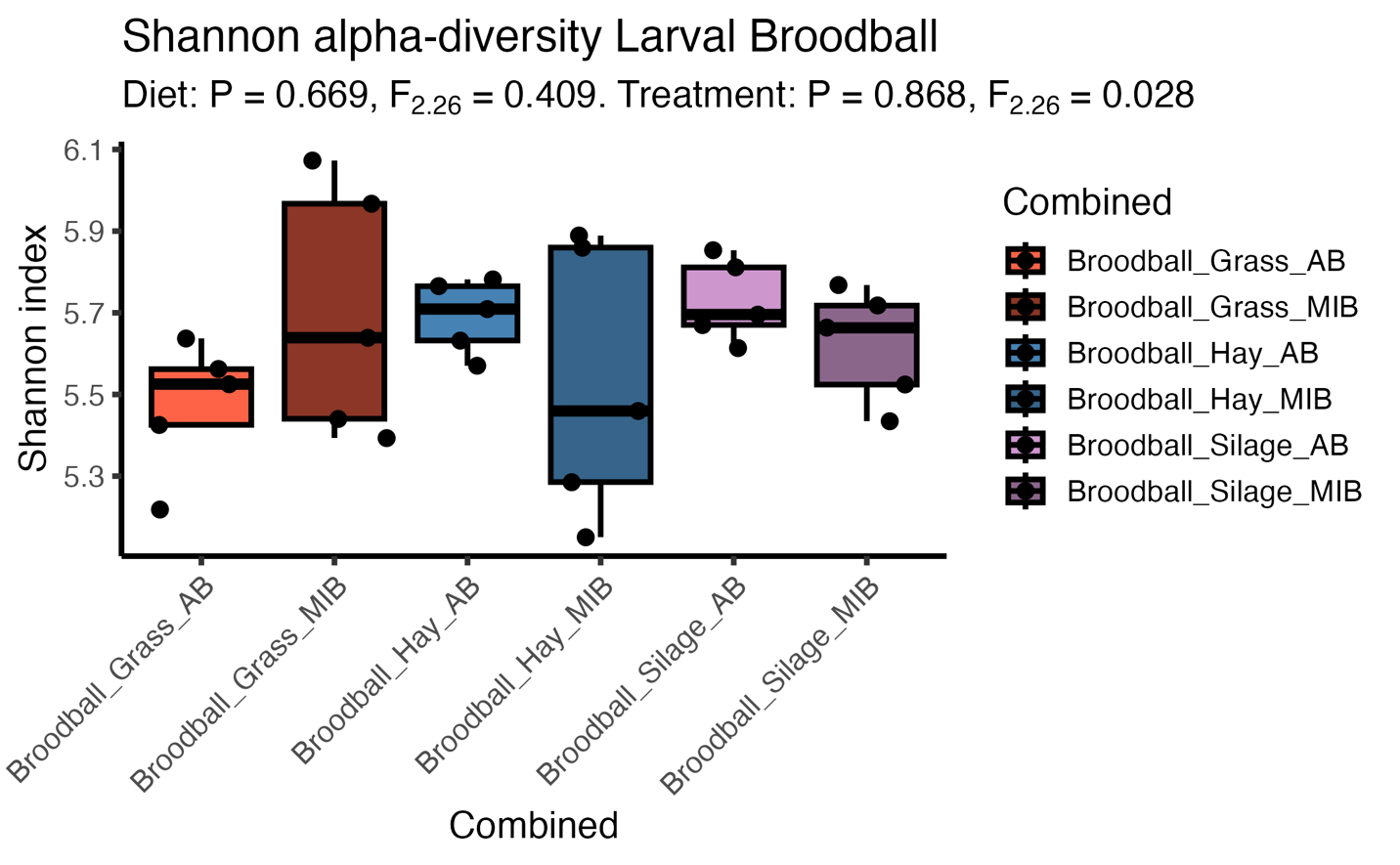


**
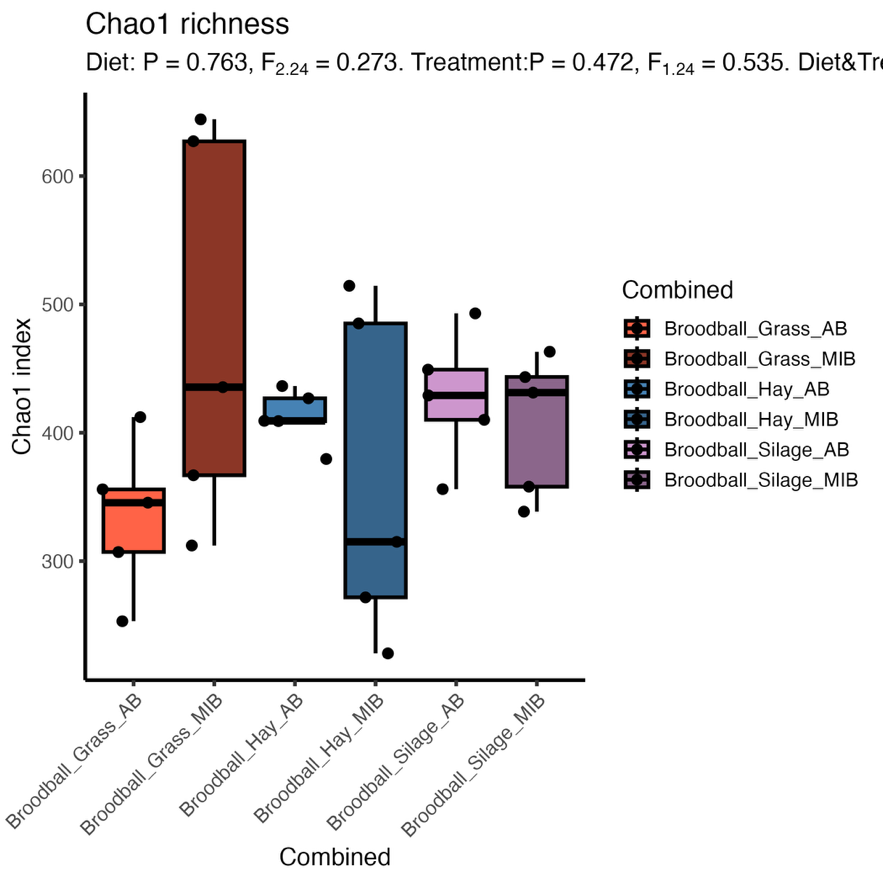
**

Figure S8. Neither diet nor the presence of a maternal microbial inoculate affected the Shannon’s alpha diversity (ANOVA: Diet *F_2,26_* = 0.409; *P* = 0.669; Treatment *F_1,26_* = 0.028; *P* = 0.868) or Chao1 richness (ANOVA: Diet *F_2,26_* = 0.233; *P* = 0.794; Treatment *F_1,26_* = 0.456; *P* = 0.506) in brood balls.


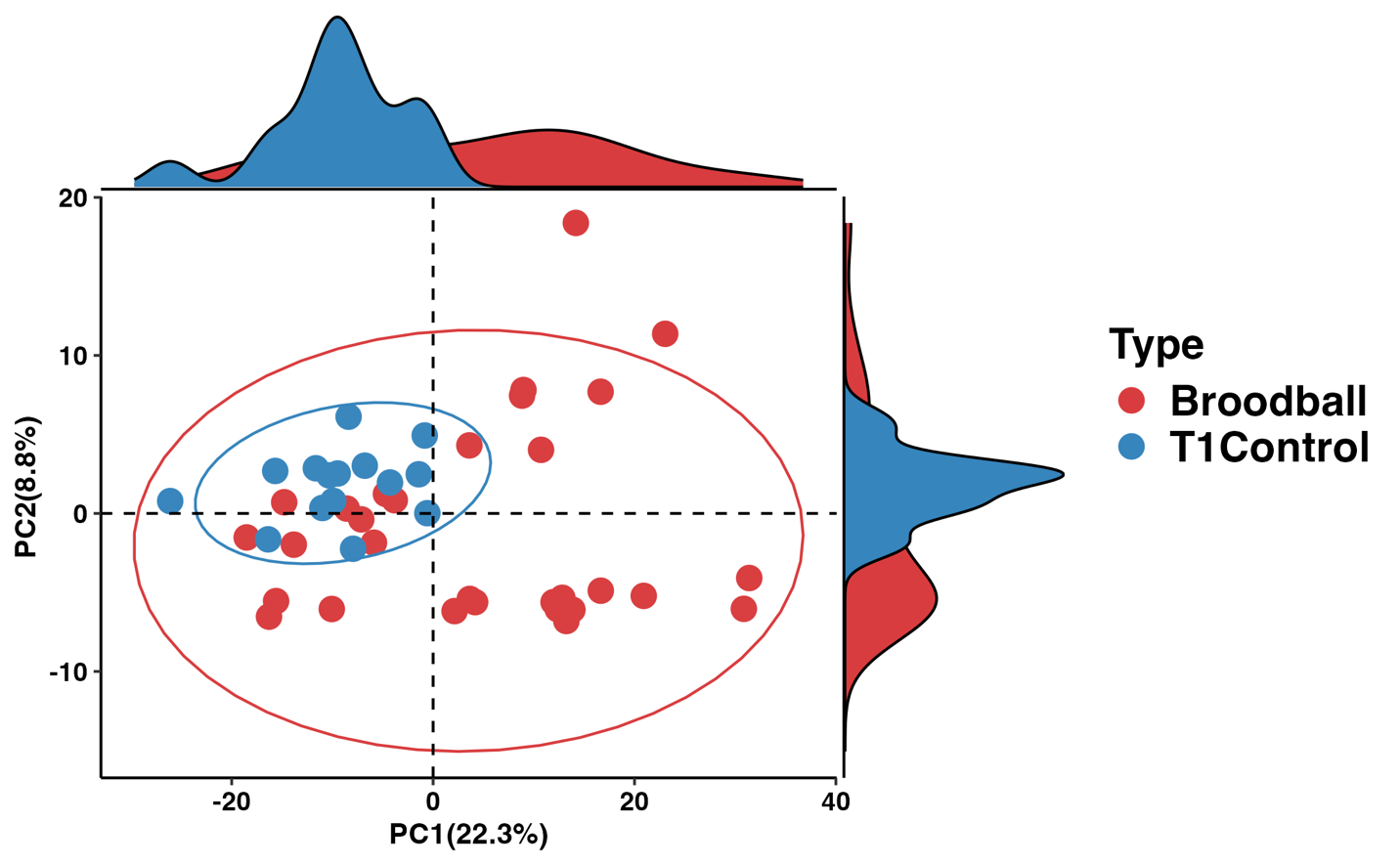


Figure S9. Principal Coordinates Analysis (PCA) of beta diversity based on bray-curtis dissimilarity in KEGG Orthology (KO) functions of all larval broodballs vs T1 Controls. Each point represents a sample, and the samples that are closer together have more similar KO functional profiles. Overall, larval brood balls and T1 Control samples are significantly different from each other (PERMANOVA, R^2^ = 0.194, *P* = 0.003). Penicillin and cephalosporin biosynthesis pathways were enriched in the SD larval brood ball. However, there were no significantly different KEGG pathways between the HD or GD larval brood balls and their respective T1 Controls.


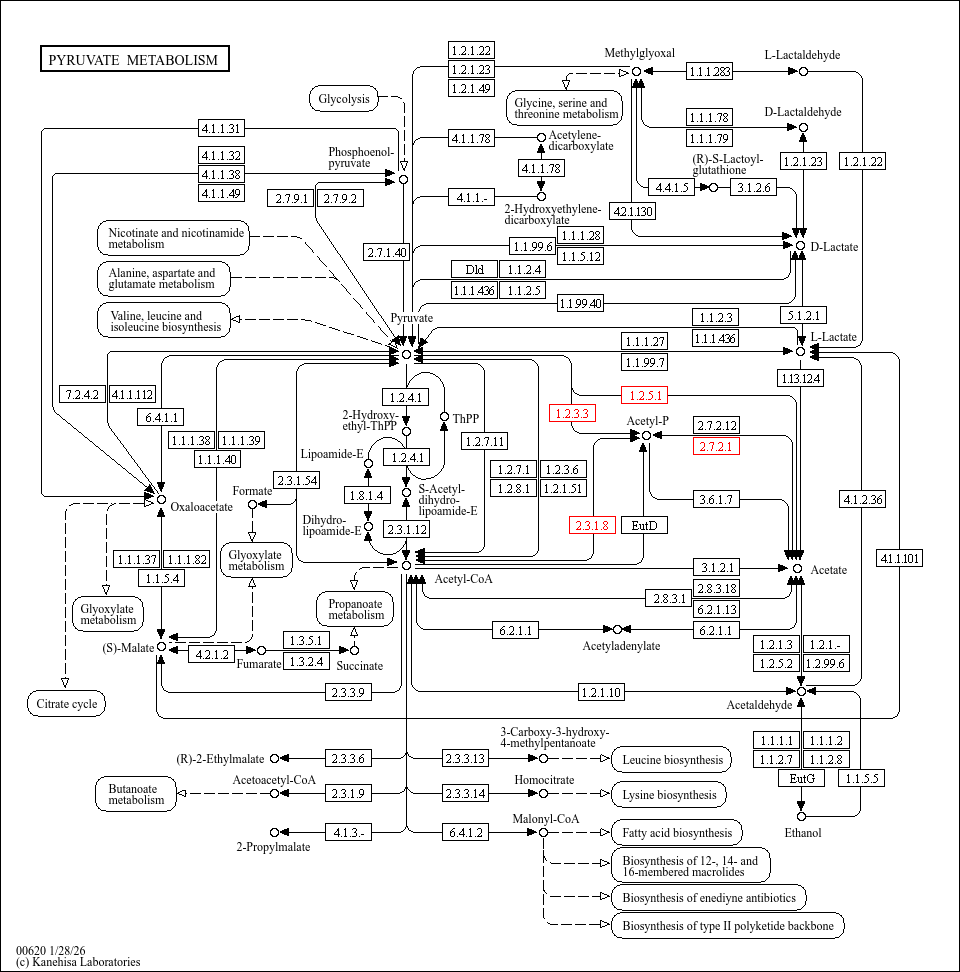


Figure S10: KEGG pathway map illustrating acetate production within pyruvate metabolism. Enzymatic steps highlighted in red represent KEGG Orthologs detected in predicted metagenomes of larval hindgut samples in this study and summarized in Table S5. These highlighted pathways indicate predicted functional potential for acetate production and were broadly present in the hindgut across larval diet groups, yet were not differentially enriched among treatments.


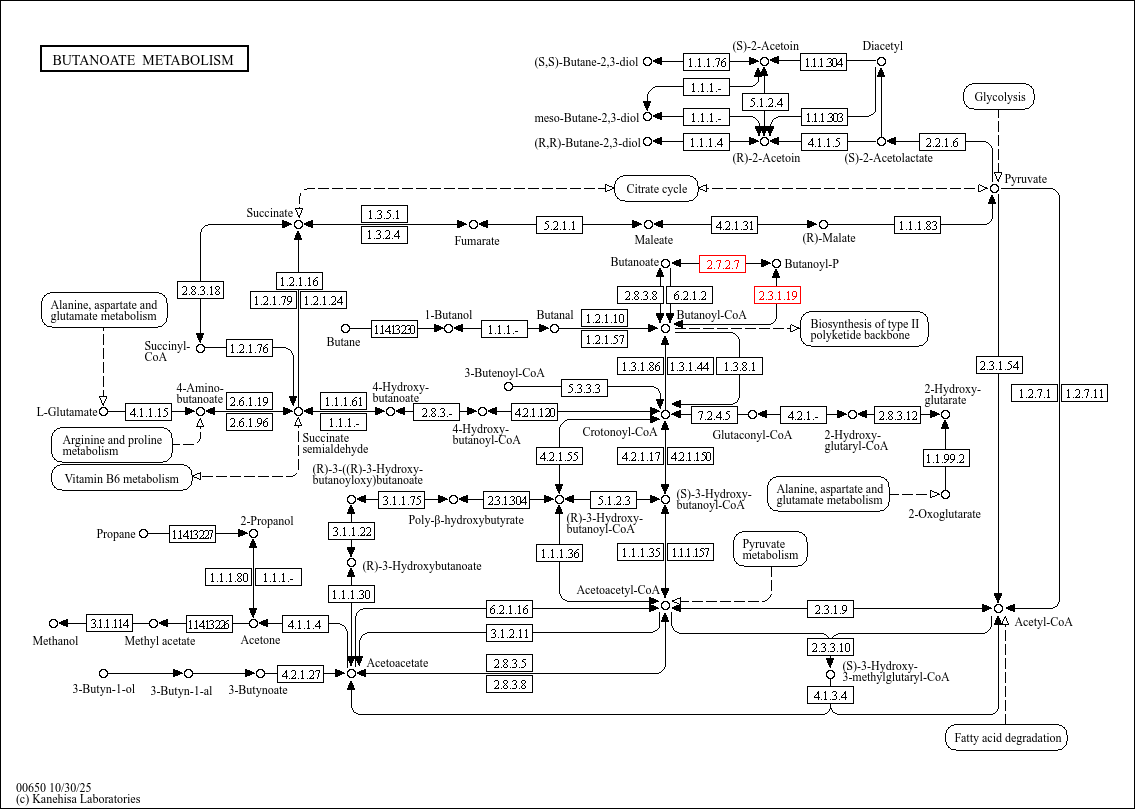


Figure S11: KEGG pathway map illustrating butyrate production within butanoate metabolism. Enzymatic steps highlighted in red represent KEGG Orthologs detected in predicted metagenomes of larval hindgut samples in this study and summarized in Table S5. These highlighted pathways indicate predicted functional potential for butyrate metabolism and were broadly present in the hindgut across larval diet groups, yet were not differentially enriched among treatments.


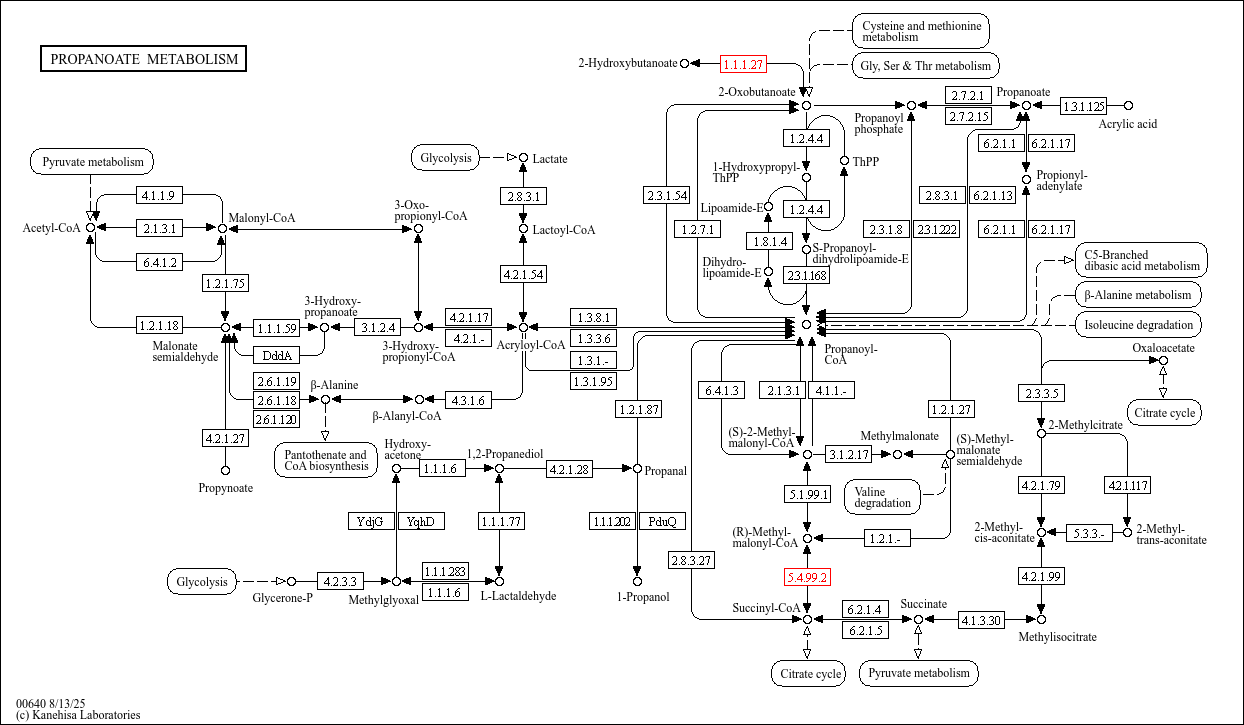


Figure S12: KEGG pathway map illustrating propionate production within propanoate metabolism. Enzymatic steps highlighted in red represent KEGG Orthologs detected in predicted metagenomes of larval hindgut samples in this study and summarized in Table S5. These highlighted pathways indicate predicted functional potential and were broadly present in the hindgut across larval diet groups, yet were not differentially enriched among treatments.

| Table S1 Full list of Indicator Species in all diet groups | | | |
| --- | --- | --- | --- |
| ISA Feature ID | ISA Taxon | ISA Diet group | Highly correlated with respective diet group |
| a425109375469315519422240485ddd4 | d__Bacteria; p__Actinomycetota; c__Actinobacteria; o__Corynebacteriales; f__Corynebacteriaceae; g__Corynebacterium | Silage |  |
| 092d1a502778a8623c96fde213b36a28 | d__Bacteria; p__Actinomycetota; c__Actinobacteria; o__Corynebacteriales; f__Dietziaceae; g__Dietzia; s__Dietzia_sp. | Silage |  |
| 1a00c433e734152c15b9daceb1bcc4f9 | d__Bacteria; p__Actinomycetota; c__Actinobacteria; o__Corynebacteriales; f__Dietziaceae; g__Dietzia; s__Dietzia_sp. | Silage | Highly correlated |
| 37845503050ebe39fa9357c083508644 | d__Bacteria; p__Actinomycetota; c__Actinobacteria; o__Corynebacteriales; f__Dietziaceae; g__Dietzia; s__Dietzia_sp. | Silage |  |
| 45fd9f9009db2aa2db19c8b7703c297f | d__Bacteria; p__Actinomycetota; c__Actinobacteria; o__Corynebacteriales; f__Dietziaceae; g__Dietzia; s__Dietzia_sp. | Silage |  |
| 4df0be2a4643acbf425b560ba2e384cd | d__Bacteria; p__Actinomycetota; c__Actinobacteria; o__Corynebacteriales; f__Dietziaceae; g__Dietzia; s__Dietzia_sp. | Silage | Highly correlated |
| 6013cb06aaa64d38178ee623e8a0360e | d__Bacteria; p__Actinomycetota; c__Actinobacteria; o__Corynebacteriales; f__Dietziaceae; g__Dietzia; s__Dietzia_sp. | Silage | Highly correlated |
| 783f997478a3614be59569ece59aeb8a | d__Bacteria; p__Actinomycetota; c__Actinobacteria; o__Corynebacteriales; f__Dietziaceae; g__Dietzia; s__Dietzia_sp. | Silage |  |
| 92e92e04c0b2b1e7413fe0c9184d37ca | d__Bacteria; p__Actinomycetota; c__Actinobacteria; o__Corynebacteriales; f__Dietziaceae; g__Dietzia; s__Dietzia_sp. | Silage |  |
| 9ccff28c7245125a8ed518bf2bc33fc6 | d__Bacteria; p__Actinomycetota; c__Actinobacteria; o__Corynebacteriales; f__Dietziaceae; g__Dietzia; s__Dietzia_sp. | Silage | Highly correlated |
| 9e0cda1021c99ec7542b99b24bea1916 | d__Bacteria; p__Actinomycetota; c__Actinobacteria; o__Corynebacteriales; f__Dietziaceae; g__Dietzia; s__Dietzia_sp. | Silage |  |
| b37b9694cd508e15a4521dea64bd392d | d__Bacteria; p__Actinomycetota; c__Actinobacteria; o__Corynebacteriales; f__Dietziaceae; g__Dietzia; s__Dietzia_sp. | Silage |  |
| b7ab363c77cf1a39c5869db770cdb8d1 | d__Bacteria; p__Actinomycetota; c__Actinobacteria; o__Corynebacteriales; f__Dietziaceae; g__Dietzia; s__Dietzia_sp. | Silage |  |
| d360ddc385dadfda8aa72c52c54cd5a5 | d__Bacteria; p__Actinomycetota; c__Actinobacteria; o__Corynebacteriales; f__Dietziaceae; g__Dietzia; s__Dietzia_sp. | Silage |  |
| e9bf3d17787db6f9f4afbffb33e9094d | d__Bacteria; p__Actinomycetota; c__Actinobacteria; o__Corynebacteriales; f__Dietziaceae; g__Dietzia; s__Dietzia_sp. | Silage |  |
| 353c78b8bacb681cae667eb3fa8de177 | d__Bacteria; p__Actinomycetota; c__Actinobacteria; o__Corynebacteriales; f__Nocardiaceae | Silage |  |
| 4fa7d602b0f8d2d0826e44a480ed53d5 | d__Bacteria; p__Actinomycetota; c__Actinobacteria; o__Corynebacteriales; f__Nocardiaceae | Silage |  |
| 8096c714fb3dcebd372092b03fef674c | d__Bacteria; p__Actinomycetota; c__Actinobacteria; o__Corynebacteriales; f__Nocardiaceae | Silage |  |
| b3637754ea76312966f7bfeb6cd64908 | d__Bacteria; p__Actinomycetota; c__Actinobacteria; o__Corynebacteriales; f__Nocardiaceae | Silage |  |
| b819d483d0f97d37d58222c4847932f0 | d__Bacteria; p__Actinomycetota; c__Actinobacteria; o__Corynebacteriales; f__Nocardiaceae | Silage |  |
| c5266ef9682d9889ac5f43758f8ab7bc | d__Bacteria; p__Actinomycetota; c__Actinobacteria; o__Corynebacteriales; f__Nocardiaceae | Silage |  |
| cc5c20aaecf02219e08446f826d923ce | d__Bacteria; p__Actinomycetota; c__Actinobacteria; o__Corynebacteriales; f__Nocardiaceae | Silage |  |
| ead2ad65f18fc4a36dadf1e04c7f40a5 | d__Bacteria; p__Actinomycetota; c__Actinobacteria; o__Corynebacteriales; f__Nocardiaceae | Silage |  |
| 648de969fd55076d1337026c11bf6190 | d__Bacteria; p__Actinomycetota; c__Actinobacteria; o__Corynebacteriales; f__Nocardiaceae; g__Rhodococcus | Silage |  |
| cf0b39dec946ca58affaddd881dce71a | d__Bacteria; p__Actinomycetota; c__Actinobacteria; o__Corynebacteriales; f__Nocardiaceae; g__Rhodococcus | Silage |  |
| cf56182fe2d5e44c71194a1f77245a55 | d__Bacteria; p__Actinomycetota; c__Actinobacteria; o__Corynebacteriales; f__Nocardiaceae; g__Rhodococcus | Silage |  |
| d4bd6408b6b52f96315fd0a6e254e9d0 | d__Bacteria; p__Actinomycetota; c__Actinobacteria; o__Corynebacteriales; f__Nocardiaceae; g__Rhodococcus | Silage |  |
| f97fd2471e0b300a51205e3efe8aee1c | d__Bacteria; p__Actinomycetota; c__Actinobacteria; o__Corynebacteriales; f__Nocardiaceae; g__Rhodococcus | Silage |  |
| 0dc550c24be5e84550357e8e8309f303 | d__Bacteria; p__Actinomycetota; c__Actinobacteria; o__Corynebacteriales; f__Nocardiaceae; g__Rhodococcus; s__Rhodococcus_rhodochrous | Silage |  |
| 1e262ef00f15f01e61aa3a43e183541b | d__Bacteria; p__Actinomycetota; c__Actinobacteria; o__Corynebacteriales; f__Nocardiaceae; g__Rhodococcus; s__Rhodococcus_rhodochrous | Silage |  |
| 2c933b3f737bd3eb338134072300f5a7 | d__Bacteria; p__Actinomycetota; c__Actinobacteria; o__Corynebacteriales; f__Nocardiaceae; g__Rhodococcus; s__Rhodococcus_rhodochrous | Silage |  |
| 43a13e1d3337224e165dabef80e73fab | d__Bacteria; p__Actinomycetota; c__Actinobacteria; o__Corynebacteriales; f__Nocardiaceae; g__Rhodococcus; s__Rhodococcus_rhodochrous | Silage |  |
| 57cd751ba2ac376df90e6cdab8e71a2d | d__Bacteria; p__Actinomycetota; c__Actinobacteria; o__Corynebacteriales; f__Nocardiaceae; g__Rhodococcus; s__Rhodococcus_rhodochrous | Silage | Highly correlated |
| 8559d8aaa5554ddd923fc46816662473 | d__Bacteria; p__Actinomycetota; c__Actinobacteria; o__Corynebacteriales; f__Nocardiaceae; g__Rhodococcus; s__Rhodococcus_rhodochrous | Silage | Highly correlated |
| 8d4aaad56cd4d9f8ff04b01ad64ad766 | d__Bacteria; p__Actinomycetota; c__Actinobacteria; o__Corynebacteriales; f__Nocardiaceae; g__Rhodococcus; s__Rhodococcus_rhodochrous | Silage |  |
| 936b30ffeb14d774a4eaf4eeee8c895e | d__Bacteria; p__Actinomycetota; c__Actinobacteria; o__Corynebacteriales; f__Nocardiaceae; g__Rhodococcus; s__Rhodococcus_rhodochrous | Silage |  |
| c01c275513a48c3e8666231a4933572c | d__Bacteria; p__Actinomycetota; c__Actinobacteria; o__Corynebacteriales; f__Nocardiaceae; g__Rhodococcus; s__Rhodococcus_rhodochrous | Silage |  |
| c407ff3fa3eabee4c892774eec771133 | d__Bacteria; p__Actinomycetota; c__Actinobacteria; o__Corynebacteriales; f__Nocardiaceae; g__Rhodococcus; s__Rhodococcus_rhodochrous | Silage | Highly correlated |
| 973fc02ba8d340ba20b88267b2bf49d3 | d__Bacteria; p__Actinomycetota; c__Actinobacteria; o__Micrococcales; f__Microbacteriaceae | Silage |  |
| ea4e302643309e30cb92150941f4ad06 | d__Bacteria; p__Actinomycetota; c__Actinobacteria; o__Micrococcales; f__Microbacteriaceae | Silage |  |
| f185d13e633c71e76b4610bc48ee89c7 | d__Bacteria; p__Actinomycetota; c__Actinobacteria; o__Micrococcales; f__Microbacteriaceae | Silage |  |
| 1dbb84535055b0af835e3d786f916c77 | d__Bacteria; p__Actinomycetota; c__Actinobacteria; o__Micrococcales; f__Microbacteriaceae; g__Pseudoclavibacter; s__Pseudoclavibacter_sp. | Silage |  |
| 37261d816787b3dabbf30f64a92cab88 | d__Bacteria; p__Actinomycetota; c__Actinobacteria; o__Micrococcales; f__Microbacteriaceae; g__Pseudoclavibacter; s__Pseudoclavibacter_sp. | Silage |  |
| 96e129797eb17b8464000c784bc2dec8 | d__Bacteria; p__Actinomycetota; c__Actinobacteria; o__Micrococcales; f__Microbacteriaceae; g__Pseudoclavibacter; s__Pseudoclavibacter_sp. | Silage |  |
| cfca0bc7fb0f3f71340ae986b8df25d1 | d__Bacteria; p__Actinomycetota; c__Actinobacteria; o__Micrococcales; f__Microbacteriaceae; g__Pseudoclavibacter; s__Pseudoclavibacter_sp. | Silage |  |
| 02257b2bfef4c8fbdd4f51d3574ec627 | d__Bacteria; p__Actinomycetota; c__Actinobacteria; o__Micrococcales; f__Micrococcaceae | Silage |  |
| 079be4d2ec54350a282004c800dcbe48 | d__Bacteria; p__Actinomycetota; c__Actinobacteria; o__Micrococcales; f__Micrococcaceae | Silage |  |
| 15cc2ec892bf170eb3f3b2cec01ffd69 | d__Bacteria; p__Actinomycetota; c__Actinobacteria; o__Micrococcales; f__Micrococcaceae | Silage |  |
| 25ad8d88ec5c8ef9ed3bbc2599147bd8 | d__Bacteria; p__Actinomycetota; c__Actinobacteria; o__Micrococcales; f__Micrococcaceae | Silage |  |
| 264433aa3b4091f2aa52a6746984e49b | d__Bacteria; p__Actinomycetota; c__Actinobacteria; o__Micrococcales; f__Micrococcaceae | Silage |  |
| 2c81e8c721f3e2b1d1428000f20ac25b | d__Bacteria; p__Actinomycetota; c__Actinobacteria; o__Micrococcales; f__Micrococcaceae | Silage |  |
| 772642abd57747b49b0323925de88ea0 | d__Bacteria; p__Actinomycetota; c__Actinobacteria; o__Micrococcales; f__Micrococcaceae | Silage |  |
| 8d6535597d34a8e45e32f41b2fc25c2f | d__Bacteria; p__Actinomycetota; c__Actinobacteria; o__Micrococcales; f__Micrococcaceae | Silage |  |
| 96a33ebd568682e6e427080c55a780be | d__Bacteria; p__Actinomycetota; c__Actinobacteria; o__Micrococcales; f__Micrococcaceae | Silage |  |
| 9e35b3fd0884813af273fb22691d3f1a | d__Bacteria; p__Actinomycetota; c__Actinobacteria; o__Micrococcales; f__Micrococcaceae | Silage |  |
| b8c06661257dbeb373b096d681cdb747 | d__Bacteria; p__Actinomycetota; c__Actinobacteria; o__Micrococcales; f__Micrococcaceae | Silage |  |
| 0497234e0702306bb808a020f144c97a | d__Bacteria; p__Actinomycetota; c__Actinobacteria; o__Micrococcales; f__Micrococcaceae; g__Kocuria | Silage |  |
| 0d5c69d2a9030f724230e6720bdf1bfd | d__Bacteria; p__Actinomycetota; c__Actinobacteria; o__Micrococcales; f__Micrococcaceae; g__Kocuria | Silage |  |
| 16801f35825416cc6ff90559163de78e | d__Bacteria; p__Actinomycetota; c__Actinobacteria; o__Micrococcales; f__Micrococcaceae; g__Kocuria | Silage |  |
| 99b3ed8871a14e4c281be0173b896ab5 | d__Bacteria; p__Actinomycetota; c__Actinobacteria; o__Micrococcales; f__Micrococcaceae; g__Kocuria | Silage |  |
| a243cb9d209a06acc7825a17e8d9c363 | d__Bacteria; p__Actinomycetota; c__Actinobacteria; o__Micrococcales; f__Micrococcaceae; g__Kocuria | Silage |  |
| bdf44a86affeab27ee24fbefd863c07b | d__Bacteria; p__Actinomycetota; c__Actinobacteria; o__Micrococcales; f__Micrococcaceae; g__Kocuria | Silage |  |
| 1dc952d74a8fef97e8c164387c8b8551 | d__Bacteria; p__Actinomycetota; c__Actinobacteria; o__Streptomycetales; f__Streptomycetaceae; g__Streptomyces | Silage |  |
| 4ec2c52cb9a9c9e50168ad2f54d91287 | d__Bacteria; p__Bacillota; c__Clostridia; o__Christensenellales; f__Christensenellaceae; g__Christensenellaceae_R-7_group | Silage |  |
| db637f2ffe685c6711cc7c8018ef2aaf | d__Bacteria; p__Bacillota; c__Clostridia; o__Christensenellales; f__Christensenellaceae; g__Christensenellaceae_R-7_group | Silage |  |
| db8046f215275a2587f250eac7b5693d | d__Bacteria; p__Bacillota; c__Clostridia; o__Christensenellales; f__Christensenellaceae; g__Christensenellaceae_R-7_group | Silage |  |
| 001a6347c327d6fa510dc46cef264f03 | d__Bacteria; p__Bacillota; c__Clostridia; o__Clostridiales; f__Clostridiaceae; g__Clostridium_sensu_stricto_1; s__uncultured_bacterium | Silage | Highly correlated |
| 17d5c3d6876708919e5199044fe8495d | d__Bacteria; p__Bacillota; c__Clostridia; o__Clostridiales; f__Clostridiaceae; g__Clostridium_sensu_stricto_1; s__uncultured_bacterium | Silage | Highly correlated |
| 3b464637eeca7f182743772189d9a1c7 | d__Bacteria; p__Bacillota; c__Clostridia; o__Clostridiales; f__Clostridiaceae; g__Clostridium_sensu_stricto_1; s__uncultured_bacterium | Silage | Highly correlated |
| 40e15bbedd6f50bdcbf8e8689bc0d24e | d__Bacteria; p__Bacillota; c__Clostridia; o__Clostridiales; f__Clostridiaceae; g__Clostridium_sensu_stricto_1; s__uncultured_bacterium | Silage | Highly correlated |
| 83f0d5b3ced211875a7a2327b9b5679f | d__Bacteria; p__Bacillota; c__Clostridia; o__Clostridiales; f__Clostridiaceae; g__Clostridium_sensu_stricto_1; s__uncultured_bacterium | Silage | Highly correlated |
| fead5e314e40d81faaa9b5fff46779f7 | d__Bacteria; p__Bacillota; c__Clostridia; o__Clostridiales; f__Clostridiaceae; g__Clostridium_sensu_stricto_1; s__uncultured_bacterium | Silage |  |
| 8798eebdf4f5e6f2cb9eab37568b1abe | d__Bacteria; p__Bacillota; c__Clostridia; o__Clostridiales; f__Clostridiaceae; g__Clostridium_sensu_stricto_13; s__bacterium_Te48R | Silage |  |
| 9677ecf7f42953e2cc851d8e767b760f | d__Bacteria; p__Bacillota; c__Clostridia; o__Clostridiales; f__Clostridiaceae; g__Clostridium_sensu_stricto_13; s__bacterium_Te48R | Silage | Highly correlated |
| b2075fdd35a250b706243f665f5e80ca | d__Bacteria; p__Bacillota; c__Clostridia; o__Clostridiales; f__Clostridiaceae; g__Clostridium_sensu_stricto_13; s__bacterium_Te48R | Silage |  |
| ec00178348d01e20206af971bbd9fdf6 | d__Bacteria; p__Bacillota; c__Clostridia; o__Clostridiales; f__Clostridiaceae; g__Clostridium_sensu_stricto_13; s__bacterium_Te48R | Silage |  |
| 73bd32cd536fa3312c7cf2d567f5b66f | d__Bacteria; p__Bacillota; c__Clostridia; o__Lachnospirales; f__Lachnospiraceae | Silage |  |
| 74a70d15c4f5857817e234f87a7cdd24 | d__Bacteria; p__Bacillota; c__Clostridia; o__Lachnospirales; f__Lachnospiraceae | Silage |  |
| 7899f8519668f532051ee5b006818517 | d__Bacteria; p__Bacillota; c__Clostridia; o__Lachnospirales; f__Lachnospiraceae | Silage |  |
| 23ac2d71f7e37f354a579b38d756d7f1 | d__Bacteria; p__Bacillota; c__Clostridia; o__Lachnospirales; f__Lachnospiraceae; g__Anaerocolumna; s__uncultured_bacterium | Silage |  |
| 2607f4ea6ee9d89cea5d4338c9af4d4e | d__Bacteria; p__Bacillota; c__Clostridia; o__Lachnospirales; f__Lachnospiraceae; g__Anaerocolumna; s__uncultured_bacterium | Silage |  |
| 5c4c35e323a49413405b234cbc618bfa | d__Bacteria; p__Bacillota; c__Clostridia; o__Lachnospirales; f__Lachnospiraceae; g__Anaerocolumna; s__uncultured_bacterium | Silage |  |
| 674b76fd9ad857d0c23dee961c14f4b9 | d__Bacteria; p__Bacillota; c__Clostridia; o__Lachnospirales; f__Lachnospiraceae; g__Anaerocolumna; s__uncultured_bacterium | Silage |  |
| 6e6454dbdb92a882a363a9e43b6cc00a | d__Bacteria; p__Bacillota; c__Clostridia; o__Lachnospirales; f__Lachnospiraceae; g__Anaerocolumna; s__uncultured_bacterium | Silage |  |
| 83735911ef561e94c43ab151f1b43943 | d__Bacteria; p__Bacillota; c__Clostridia; o__Lachnospirales; f__Lachnospiraceae; g__Anaerocolumna; s__uncultured_bacterium | Silage |  |
| 8e8fb402d0511785c85d2c56d864670f | d__Bacteria; p__Bacillota; c__Clostridia; o__Lachnospirales; f__Lachnospiraceae; g__Anaerocolumna; s__uncultured_bacterium | Silage |  |
| a27b13e7d68d80b9ac6a139c02fab990 | d__Bacteria; p__Bacillota; c__Clostridia; o__Lachnospirales; f__Lachnospiraceae; g__Anaerocolumna; s__uncultured_bacterium | Silage |  |
| b241db86e6f8a10b3f291cc29e4d34cb | d__Bacteria; p__Bacillota; c__Clostridia; o__Lachnospirales; f__Lachnospiraceae; g__Anaerocolumna; s__uncultured_bacterium | Silage |  |
| b2ab9ff9851b22ff76474d1bb6788423 | d__Bacteria; p__Bacillota; c__Clostridia; o__Lachnospirales; f__Lachnospiraceae; g__Anaerocolumna; s__uncultured_bacterium | Silage |  |
| d19255fbd8fcd3168c6686cc739dd241 | d__Bacteria; p__Bacillota; c__Clostridia; o__Lachnospirales; f__Lachnospiraceae; g__Anaerocolumna; s__uncultured_bacterium | Silage |  |
| d87f05b849c170afbf210ed10a9285ce | d__Bacteria; p__Bacillota; c__Clostridia; o__Lachnospirales; f__Lachnospiraceae; g__Anaerocolumna; s__uncultured_bacterium | Silage |  |
| dbefb9260225e584428c7b956aba60e9 | d__Bacteria; p__Bacillota; c__Clostridia; o__Lachnospirales; f__Lachnospiraceae; g__Anaerocolumna; s__uncultured_bacterium | Silage |  |
| e1c18e5e63c45285cfc59436910ea417 | d__Bacteria; p__Bacillota; c__Clostridia; o__Lachnospirales; f__Lachnospiraceae; g__Anaerocolumna; s__uncultured_bacterium | Silage |  |
| e7c04320d282081ecc92a872aab2465e | d__Bacteria; p__Bacillota; c__Clostridia; o__Lachnospirales; f__Lachnospiraceae; g__Anaerocolumna; s__uncultured_bacterium | Silage |  |
| ebb3d1280cdcde2dd12cfff3972863c8 | d__Bacteria; p__Bacillota; c__Clostridia; o__Lachnospirales; f__Lachnospiraceae; g__Anaerocolumna; s__uncultured_bacterium | Silage |  |
| fadec9fa63424841d7dda3e2a5763b3e | d__Bacteria; p__Bacillota; c__Clostridia; o__Lachnospirales; f__Lachnospiraceae; g__Anaerocolumna; s__uncultured_bacterium | Silage |  |
| 4eaf46fbc675528fc3d059500ce6682e | d__Bacteria; p__Bacillota; c__Clostridia; o__Lachnospirales; f__Lachnospiraceae; g__Anaerostignum; s__uncultured_Clostridium | Silage |  |
| 5284c8c48e47d9243d1a3a48683d6af6 | d__Bacteria; p__Bacillota; c__Clostridia; o__Lachnospirales; f__Lachnospiraceae; g__Anaerostignum; s__uncultured_Clostridium | Silage | Highly correlated |
| 5853c5d0b6dadda79a4c0522af1f81b8 | d__Bacteria; p__Bacillota; c__Clostridia; o__Lachnospirales; f__Lachnospiraceae; g__Lachnospiraceae_UCG-009; s__uncultured_rumen | Silage |  |
| dee184f4ca5e387cad0db50804d831f4 | d__Bacteria; p__Bacillota; c__Clostridia; o__Lachnospirales; f__Lachnospiraceae; g__Lachnospiraceae_UCG-009; s__uncultured_rumen | Silage |  |
| 511577123e1757d2fa26982a0ef2376d | d__Bacteria; p__Bacillota; c__Clostridia; o__Lachnospirales; f__Lachnospiraceae; g__Marvinbryantia; s__uncultured_bacterium | Silage |  |
| 88893320012e2aafb70a8fca1a09c505 | d__Bacteria; p__Bacillota; c__Clostridia; o__Lachnospirales; f__Lachnospiraceae; g__Marvinbryantia; s__uncultured_bacterium | Silage |  |
| 8a9affc852986d47ab84940b8aa44b4f | d__Bacteria; p__Bacillota; c__Clostridia; o__Lachnospirales; f__Lachnospiraceae; g__Marvinbryantia; s__uncultured_bacterium | Silage | Highly correlated |
| ac08b5302d750563abb349643ebd1780 | d__Bacteria; p__Bacillota; c__Clostridia; o__Lachnospirales; f__Lachnospiraceae; g__Marvinbryantia; s__uncultured_bacterium | Silage |  |
| b849a0b3fdf1836e53deb9e4fbe34f16 | d__Bacteria; p__Bacillota; c__Clostridia; o__Lachnospirales; f__Lachnospiraceae; g__Marvinbryantia; s__uncultured_bacterium | Silage |  |
| fe4597496bf10963f3098f3ff38ce6bc | d__Bacteria; p__Bacillota; c__Clostridia; o__Lachnospirales; f__Lachnospiraceae; g__Marvinbryantia; s__uncultured_bacterium | Silage |  |
| 0132088b7ac6645f2cd6f952b1364347 | d__Bacteria; p__Bacillota; c__Clostridia; o__Lachnospirales; f__Lachnospiraceae; g__uncultured; s__uncultured_bacterium | Silage |  |
| a6fe38f365a1c849ec82b57ecc781df4 | d__Bacteria; p__Bacillota; c__Clostridia; o__Lachnospirales; f__Lachnospiraceae; g__uncultured; s__uncultured_bacterium | Silage |  |
| a7a7ab68f15e7d2efc59292382b13df6 | d__Bacteria; p__Bacillota; c__Clostridia; o__Lachnospirales; f__Lachnospiraceae; g__uncultured; s__uncultured_bacterium | Silage |  |
| f916654a6a2c6d7e99bf26dbb01e5b79 | d__Bacteria; p__Bacillota; c__Clostridia; o__Lachnospirales; f__Lachnospiraceae; g__uncultured; s__uncultured_bacterium | Silage |  |
| fc35b49c046ad38b96047f629782857c | d__Bacteria; p__Bacillota; c__Clostridia; o__Lachnospirales; f__Lachnospiraceae; g__uncultured; s__uncultured_bacterium | Silage |  |
| eae3467cf7de5d3c0a17e781de7471bf | d__Bacteria; p__Bacillota; c__Clostridia; o__Oscillospirales; f__Oscillospiraceae; g__Colidextribacter; s__uncultured_bacterium | Silage |  |
| fb63d7153c6c85a25a564aeabe25293d | d__Bacteria; p__Bacillota; c__Clostridia; o__Oscillospirales; f__Oscillospiraceae; g__Colidextribacter; s__uncultured_bacterium | Silage |  |
| d1badaba9517f0c15642325d33650e5b | d__Bacteria; p__Bacillota; c__Clostridia; o__Peptostreptococcales-Tissierellales | Silage |  |
| 5c82dceabd3954ae1998aff30ecddbce | d__Bacteria; p__Bacillota; c__Clostridia; o__Peptostreptococcales-Tissierellales; f__Anaerovoracaceae; g__Anaerovorax; s__uncultured_Bacillota | Silage |  |
| 788d56563ed7e8f4be895ae2ddc25288 | d__Bacteria; p__Bacillota; c__Clostridia; o__Peptostreptococcales-Tissierellales; f__Peptostreptococcaceae; g__Paeniclostridium | Silage | Highly correlated |
| b353831e964af2fec47e224e4c25fc56 | d__Bacteria; p__Bacillota; c__Clostridia; o__Peptostreptococcales-Tissierellales; f__Peptostreptococcaceae; g__Paeniclostridium | Silage |  |
| cf919e72e917be4c7d53fb5866f5b9b6 | d__Bacteria; p__Bacillota; c__Clostridia; o__Peptostreptococcales-Tissierellales; f__Peptostreptococcales-Tissierellales; g__Serpentinicella; s__uncultured_bacterium | Silage |  |
| ec0ff447404d1a43e3bfd1996d822b1e | d__Bacteria; p__Bacillota; c__Clostridia; o__Peptostreptococcales-Tissierellales; f__Peptostreptococcales-Tissierellales; g__Serpentinicella; s__uncultured_bacterium | Silage |  |
| 4970cad5e8b992aacedb8f904102efe2 | d__Bacteria; p__Psuedomonadota; c__AlphaPsuedomonadota; o__Rhizobiales | Silage |  |
| 778b65c55da0ecb7d41c831b7d81022d | d__Bacteria; p__Psuedomonadota; c__AlphaPsuedomonadota; o__Rhizobiales | Silage |  |
| cb1db646732a62395342918bab169adf | d__Bacteria; p__Psuedomonadota; c__AlphaPsuedomonadota; o__Rhizobiales | Silage |  |
| ebbce7a351d9aaa04de6ad0cf74f57ec | d__Bacteria; p__Psuedomonadota; c__AlphaPsuedomonadota; o__Rhizobiales | Silage |  |
| a9677c0270224ade2f79651cf7a5905d | d__Bacteria; p__Psuedomonadota; c__AlphaPsuedomonadota; o__Rhizobiales; f__Devosiaceae; g__Devosia; s__Devosia_riboflavina | Silage |  |
| c2b84ddc945db7eb466a2f2eb4566e79 | d__Bacteria; p__Psuedomonadota; c__AlphaPsuedomonadota; o__Rhizobiales; f__Devosiaceae; g__Devosia; s__Devosia_riboflavina | Silage | Highly correlated |
| d47a4452536bf770a0b8f78636af2258 | d__Bacteria; p__Psuedomonadota; c__AlphaPsuedomonadota; o__Rhizobiales; f__Devosiaceae; g__Devosia; s__Devosia_riboflavina | Silage |  |
| 36ca7cdae4111f048046bd08c53aef73 | d__Bacteria; p__Psuedomonadota; c__AlphaPsuedomonadota; o__Sphingomonadales; f__Sphingomonadaceae; g__Novosphingobium | Silage |  |
| 3857b6974c3e9218b0e1d91ca768af08 | d__Bacteria; p__Psuedomonadota; c__AlphaPsuedomonadota; o__Sphingomonadales; f__Sphingomonadaceae; g__Novosphingobium | Silage |  |
| 624743d8f08ea31c7ca9d353acbe099c | d__Bacteria; p__Psuedomonadota; c__AlphaPsuedomonadota; o__Sphingomonadales; f__Sphingomonadaceae; g__Novosphingobium | Silage |  |
| bd74426a87dfd4efafe55303f66fd3cd | d__Bacteria; p__Psuedomonadota; c__AlphaPsuedomonadota; o__Sphingomonadales; f__Sphingomonadaceae; g__Novosphingobium | Silage |  |
| d3c15d2732456d9d45b1986a4ee43a38 | d__Bacteria; p__Psuedomonadota; c__AlphaPsuedomonadota; o__Sphingomonadales; f__Sphingomonadaceae; g__Novosphingobium | Silage |  |
| 3d672fb45e9c21ed2d62977cbf46c849 | d__Bacteria; p__Actinomycetota; c__Acidimicrobiia; o__Microtrichales; f__Microtrichaceae; g__uncultured; s__uncultured_bacterium | Hay |  |
| 501b7bf09084c61cd5a5075528beaced | d__Bacteria; p__Actinomycetota; c__Acidimicrobiia; o__Microtrichales; f__Microtrichaceae; g__uncultured; s__uncultured_bacterium | Hay |  |
| 64ac9c053c14fee458691419558547fe | d__Bacteria; p__Actinomycetota; c__Acidimicrobiia; o__Microtrichales; f__Microtrichaceae; g__uncultured; s__uncultured_bacterium | Hay |  |
| 6f2235029ff569de3cafd6a52cf68592 | d__Bacteria; p__Actinomycetota; c__Acidimicrobiia; o__Microtrichales; f__Microtrichaceae; g__uncultured; s__uncultured_bacterium | Hay |  |
| 71ed7bc8d3f22300ee5d2a65f665731b | d__Bacteria; p__Actinomycetota; c__Acidimicrobiia; o__Microtrichales; f__Microtrichaceae; g__uncultured; s__uncultured_bacterium | Hay |  |
| 7e13b9bc9f62547f4149ce8a2e607d1f | d__Bacteria; p__Actinomycetota; c__Acidimicrobiia; o__Microtrichales; f__Microtrichaceae; g__uncultured; s__uncultured_bacterium | Hay |  |
| e5268465761f4f85bbe06d49dff8af0b | d__Bacteria; p__Actinomycetota; c__Acidimicrobiia; o__Microtrichales; f__Microtrichaceae; g__uncultured; s__uncultured_bacterium | Hay | Highly correlated |
| f51f0b642bc1b88fca5b97655c1bb0af | d__Bacteria; p__Actinomycetota; c__Acidimicrobiia; o__Microtrichales; f__Microtrichaceae; g__uncultured; s__uncultured_bacterium | Hay | Highly correlated |
| fad6ce297087fda6b970b866a7277b1b | d__Bacteria; p__Actinomycetota; c__Acidimicrobiia; o__Microtrichales; f__Microtrichaceae; g__uncultured; s__uncultured_bacterium | Hay |  |
| 286589c85c50b1e2f788a4cd9530c774 | d__Bacteria; p__Actinomycetota; c__Actinobacteria; o__Micrococcales; f__Micrococcaceae | Hay |  |
| 62824e8d315610bc181aff54b86b7111 | d__Bacteria; p__Actinomycetota; c__Actinobacteria; o__Micrococcales; f__Micrococcaceae | Hay |  |
| 848f16d3efe3ee7964b1443c42a1d8a0 | d__Bacteria; p__Actinomycetota; c__Actinobacteria; o__Micrococcales; f__Micrococcaceae | Hay |  |
| bd3d3d72f5de1b751f33f3ace3641b51 | d__Bacteria; p__Actinomycetota; c__Actinobacteria; o__Micrococcales; f__Micrococcaceae | Hay |  |
| f5c7eb397542a02268a9347ac8b961bf | d__Bacteria; p__Actinomycetota; c__Actinobacteria; o__Micrococcales; f__Micrococcaceae | Hay |  |
| 399810bb299a88794dd66ad18af99db9 | d__Bacteria; p__Actinomycetota; c__Actinobacteria; o__Micrococcales; f__Micrococcaceae; g__Arthrobacter; s__Arthrobacter_sp. | Hay | Highly correlated |
| 400f466429c8df3b6544f6346e1f85a7 | d__Bacteria; p__Actinomycetota; c__Actinobacteria; o__Micrococcales; f__Micrococcaceae; g__Arthrobacter; s__Arthrobacter_sp. | Hay |  |
| 7c2ca646bd0b9fdab76c6c569504a8a8 | d__Bacteria; p__Actinomycetota; c__Actinobacteria; o__Micrococcales; f__Micrococcaceae; g__Arthrobacter; s__Arthrobacter_sp. | Hay |  |
| b531a5cb0a5bc6e8c5908f58108e48a9 | d__Bacteria; p__Actinomycetota; c__Actinobacteria; o__Micrococcales; f__Micrococcaceae; g__Arthrobacter; s__Arthrobacter_sp. | Hay |  |
| 31ad0d6535de138825a8069032499963 | d__Bacteria; p__Actinomycetota; c__Actinobacteria; o__Micrococcales; f__Micrococcaceae; g__Kocuria | Hay | Highly correlated |
| afcb367a60e7440f741a00edafa57ece | d__Bacteria; p__Actinomycetota; c__Actinobacteria; o__Micrococcales; f__Micrococcaceae; g__Kocuria | Hay | Highly correlated |
| db8b461299effcb498bf2bfb5331fda0 | d__Bacteria; p__Actinomycetota; c__Actinobacteria; o__Micrococcales; f__Micrococcaceae; g__Kocuria | Hay | Highly correlated |
| f63f4e22824d59e2ccb906e3e66e942e | d__Bacteria; p__Actinomycetota; c__Actinobacteria; o__Micrococcales; f__Micrococcaceae; g__Kocuria | Hay | Highly correlated |
| 3880f0a107c7e9e3af4a85f7cf61e975 | d__Bacteria; p__Actinomycetota; c__Actinobacteria; o__Micrococcales; f__Micrococcaceae; g__Kocuria; s__Kocuria_flava | Hay | Highly correlated |
| 965f80756270460d53ebd59d80291358 | d__Bacteria; p__Actinomycetota; c__Actinobacteria; o__Micrococcales; f__Micrococcaceae; g__Kocuria; s__Kocuria_flava | Hay | Highly correlated |
| ab0935255d5e24b52fe55548fbd6331b | d__Bacteria; p__Actinomycetota; c__Actinobacteria; o__Micrococcales; f__Micrococcaceae; g__Kocuria; s__Kocuria_flava | Hay | Highly correlated |
| becdabcbc16a242819a042dcb9b5988b | d__Bacteria; p__Actinomycetota; c__Actinobacteria; o__Micrococcales; f__Micrococcaceae; g__Kocuria; s__Kocuria_flava | Hay | Highly correlated |
| 04e1ccba7cd2b971cbeb29b39655240f | d__Bacteria; p__Actinomycetota; c__Actinobacteria; o__Propionibacteriales; f__Nocardioidaceae; g__Aeromicrobium; s__uncultured_bacterium | Hay |  |
| 098fbde8c50c6fd64766576a1e2c76ec | d__Bacteria; p__Actinomycetota; c__Actinobacteria; o__Propionibacteriales; f__Nocardioidaceae; g__Aeromicrobium; s__uncultured_bacterium | Hay |  |
| 2b5a0ef39d93c385668669af739b9bfb | d__Bacteria; p__Actinomycetota; c__Actinobacteria; o__Propionibacteriales; f__Nocardioidaceae; g__Aeromicrobium; s__uncultured_bacterium | Hay |  |
| 96c22324641877db2e2014e408125d27 | d__Bacteria; p__Actinomycetota; c__Actinobacteria; o__Propionibacteriales; f__Nocardioidaceae; g__Aeromicrobium; s__uncultured_bacterium | Hay |  |
| db6fc7de377acdd459cb862b1a8628ee | d__Bacteria; p__Actinomycetota; c__Actinobacteria; o__Propionibacteriales; f__Nocardioidaceae; g__Aeromicrobium; s__uncultured_bacterium | Hay |  |
| 99e1c344fe962a2ca899f2aac9a93024 | d__Bacteria; p__Actinomycetota; c__Actinobacteria; o__Propionibacteriales; f__Propionibacteriaceae | Hay |  |
| 4f78a95348652c49f82d39751f416fb2 | d__Bacteria; p__Actinomycetota; c__Actinobacteria; o__Propionibacteriales; f__Propionibacteriaceae; g__Marinilutecoccus; s__unidentified | Hay | Highly correlated |
| 58a7b7b1771ca643b0db8965fbf35136 | d__Bacteria; p__Actinomycetota; c__Actinobacteria; o__Propionibacteriales; f__Propionibacteriaceae; g__Marinilutecoccus; s__unidentified | Hay | Highly correlated |
| 6a54b20c2ec0304ed97779c81765a015 | d__Bacteria; p__Actinomycetota; c__Actinobacteria; o__Propionibacteriales; f__Propionibacteriaceae; g__Marinilutecoccus; s__unidentified | Hay | Highly correlated |
| 836e4caf2b77b16e0904db4a2589a8a4 | d__Bacteria; p__Actinomycetota; c__Actinobacteria; o__Propionibacteriales; f__Propionibacteriaceae; g__Marinilutecoccus; s__unidentified | Hay | Highly correlated |
| 87fe22feff974805fb29464a01f9d07c | d__Bacteria; p__Actinomycetota; c__Actinobacteria; o__Propionibacteriales; f__Propionibacteriaceae; g__Marinilutecoccus; s__unidentified | Hay | Highly correlated |
| 9f4a2d3ff0efc82892bdf059295012c5 | d__Bacteria; p__Actinomycetota; c__Actinobacteria; o__Propionibacteriales; f__Propionibacteriaceae; g__Marinilutecoccus; s__unidentified | Hay | Highly correlated |
| cbcedad1fbfcf0e03fe9f287ba9d7613 | d__Bacteria; p__Actinomycetota; c__Actinobacteria; o__Propionibacteriales; f__Propionibacteriaceae; g__Marinilutecoccus; s__unidentified | Hay | Highly correlated |
| fa435e67f679b190843742ad87801606 | d__Bacteria; p__Actinomycetota; c__Actinobacteria; o__Propionibacteriales; f__Propionibacteriaceae; g__Marinilutecoccus; s__unidentified | Hay | Highly correlated |
| 28afebcfda3409eda437f145d46aeb4b | d__Bacteria; p__Actinomycetota; c__Coriobacteriia; o__Coriobacteriales; f__Coriobacteriales_Incertae_Sedis; g__Raoultibacter; s__metagenome | Hay |  |
| 78713206dc121eb98593cb2a6a189591 | d__Bacteria; p__Actinomycetota; c__Coriobacteriia; o__Coriobacteriales; f__Coriobacteriales_Incertae_Sedis; g__Raoultibacter; s__metagenome | Hay |  |
| ecbd2e66f9a53d31344f8fe162224c62 | d__Bacteria; p__Actinomycetota; c__Coriobacteriia; o__Coriobacteriales; f__Coriobacteriales_Incertae_Sedis; g__Raoultibacter; s__metagenome | Hay |  |
| fa0dab5cfa416f2846127f0799360d63 | d__Bacteria; p__Actinomycetota; c__Coriobacteriia; o__Coriobacteriales; f__Coriobacteriales_Incertae_Sedis; g__Raoultibacter; s__metagenome | Hay |  |
| 37f017ad78f60e00272f0047b8f33a41 | d__Bacteria; p__Cyanobacteria; c__Cyanobacteriia | Hay |  |
| 257f18f783d8da7fb188c908b9a7c488 | d__Bacteria; p__Bacillota; c__Clostridia; o__Lachnospirales; f__Lachnospiraceae | Hay |  |
| f825beda1748ad5b63b430558f48967c | d__Bacteria; p__Bacillota; c__Clostridia; o__Lachnospirales; f__Lachnospiraceae | Hay |  |
| 0ee96c9795b82bf16afc1e057c4e1ac9 | d__Bacteria; p__Bacillota; c__Clostridia; o__Lachnospirales; f__Lachnospiraceae; g__Anaerocolumna; s__uncultured_bacterium | Hay |  |
| 17794fbb72cd5a0ba35c18575f90e608 | d__Bacteria; p__Bacillota; c__Clostridia; o__Lachnospirales; f__Lachnospiraceae; g__Anaerocolumna; s__uncultured_bacterium | Hay |  |
| 41af6d5976b1311a07e8719712630c4d | d__Bacteria; p__Bacillota; c__Clostridia; o__Lachnospirales; f__Lachnospiraceae; g__Anaerocolumna; s__uncultured_bacterium | Hay |  |
| 478b406bd3e46917ad8d8693a9372e3d | d__Bacteria; p__Bacillota; c__Clostridia; o__Lachnospirales; f__Lachnospiraceae; g__Anaerocolumna; s__uncultured_bacterium | Hay |  |
| 531998dc532e69ef8317bebc10ca0e67 | d__Bacteria; p__Bacillota; c__Clostridia; o__Lachnospirales; f__Lachnospiraceae; g__Anaerocolumna; s__uncultured_bacterium | Hay |  |
| 5e480c82484c6de65a45512539ef7b77 | d__Bacteria; p__Bacillota; c__Clostridia; o__Lachnospirales; f__Lachnospiraceae; g__Anaerocolumna; s__uncultured_bacterium | Hay |  |
| 61b54d45f11edf78446674a31f5eecf5 | d__Bacteria; p__Bacillota; c__Clostridia; o__Lachnospirales; f__Lachnospiraceae; g__Anaerocolumna; s__uncultured_bacterium | Hay |  |
| 68928bdd2c368931cfbab3e64ae59ff3 | d__Bacteria; p__Bacillota; c__Clostridia; o__Lachnospirales; f__Lachnospiraceae; g__Anaerocolumna; s__uncultured_bacterium | Hay |  |
| 6be9e7b04222f09d686e1166f0ba2fa4 | d__Bacteria; p__Bacillota; c__Clostridia; o__Lachnospirales; f__Lachnospiraceae; g__Anaerocolumna; s__uncultured_bacterium | Hay |  |
| 7f5be932dd8f3902357fb7af2f5328bd | d__Bacteria; p__Bacillota; c__Clostridia; o__Lachnospirales; f__Lachnospiraceae; g__Anaerocolumna; s__uncultured_bacterium | Hay |  |
| 813e425fd3ff3387c89cd0d74ac2345a | d__Bacteria; p__Bacillota; c__Clostridia; o__Lachnospirales; f__Lachnospiraceae; g__Anaerocolumna; s__uncultured_bacterium | Hay |  |
| a7eb4727acdac70dc2c2fbc2d439b913 | d__Bacteria; p__Bacillota; c__Clostridia; o__Lachnospirales; f__Lachnospiraceae; g__Anaerocolumna; s__uncultured_bacterium | Hay |  |
| b63ca3d67d7d20c21acf2cc78bbce7a2 | d__Bacteria; p__Bacillota; c__Clostridia; o__Lachnospirales; f__Lachnospiraceae; g__Anaerocolumna; s__uncultured_bacterium | Hay |  |
| c694eaee2860785cff921e989f12b590 | d__Bacteria; p__Bacillota; c__Clostridia; o__Lachnospirales; f__Lachnospiraceae; g__Anaerocolumna; s__uncultured_bacterium | Hay |  |
| 21cf742539a2b7aa14b53f6c6bbcfd8a | d__Bacteria; p__Bacillota; c__Clostridia; o__Oscillospirales; f__Ruminococcaceae; g__Incertae_Sedis; s__uncultured_bacterium | Hay |  |
| bdb093e451ca21fd2451e75e028076d8 | d__Bacteria; p__Bacillota; c__Clostridia; o__Peptostreptococcales-Tissierellales; f__Peptostreptococcaceae; g__uncultured; s__uncultured_bacterium | Hay |  |
| 8580145427e876ab4e6440bd90b22cd5 | d__Bacteria; p__Bacillota; c__Clostridia; o__Peptostreptococcales-Tissierellales; f__Sedimentibacteraceae; g__Sedimentibacter; s__uncultured_bacterium | Hay |  |
| 89582396241ade5a7f609bac75dd0871 | d__Bacteria; p__Bacillota; c__Clostridia; o__Peptostreptococcales-Tissierellales; f__Sedimentibacteraceae; g__Sedimentibacter; s__uncultured_bacterium | Hay |  |
| dd59e3c4b1cfa73f4e30a2e246560fe5 | d__Bacteria; p__Patescibacteria; c__Saccharimonadia; o__Saccharimonadales | Hay |  |
| c127743b07991bd476de677f25cf5dc1 | d__Bacteria; p__Patescibacteria; c__Saccharimonadia; o__Saccharimonadales; f__Saccharimonadaceae; g__TM7a; s__uncultured_bacterium | Hay |  |
| 4274c323363b569769ba2bc1f45ffbce | d__Bacteria; p__Actinomycetota; c__Actinobacteria; o__Corynebacteriales; f__Mycobacteriaceae; g__Mycobacterium | Grass |  |
| 3968ebdd98c0192401577092247cc10d | d__Bacteria; p__Actinomycetota; c__Actinobacteria; o__Corynebacteriales; f__Mycobacteriaceae; g__Mycobacterium; s__Mycobacterium_sp. | Grass |  |
| 735c3b945e319b8dcc20195ae61e99ff | d__Bacteria; p__Actinomycetota; c__Actinobacteria; o__Corynebacteriales; f__Mycobacteriaceae; g__Mycobacterium; s__Mycobacterium_sp. | Grass |  |
| a04df4ba075e3a5bda77cf19a63ccc06 | d__Bacteria; p__Actinomycetota; c__Actinobacteria; o__Corynebacteriales; f__Mycobacteriaceae; g__Mycobacterium; s__Mycobacterium_sp. | Grass |  |
| c16be9a8a574e8f694107bf004ad731a | d__Bacteria; p__Actinomycetota; c__Actinobacteria; o__Corynebacteriales; f__Mycobacteriaceae; g__Mycobacterium; s__Mycobacterium_sp. | Grass |  |
| 6f2f34fd18ab62efc270e9a60934909d | d__Bacteria; p__Actinomycetota; c__Actinobacteria; o__Corynebacteriales; f__Nocardiaceae; g__Rhodococcus; s__Rhodococcus_erythropolis | Grass |  |
| da1d2d9a2132cbf50aeee9e95fb6083f | d__Bacteria; p__Actinomycetota; c__Actinobacteria; o__Corynebacteriales; f__Nocardiaceae; g__Rhodococcus; s__Rhodococcus_erythropolis | Grass |  |
| 041d0969bd916270dc8df6f75fe23174 | d__Bacteria; p__Actinomycetota; c__Actinobacteria; o__Micrococcales; f__Micrococcaceae | Grass | Highly correlated |
| 18551e8178debb58fe80efb03e83739e | d__Bacteria; p__Actinomycetota; c__Actinobacteria; o__Micrococcales; f__Micrococcaceae | Grass |  |
| 31e0482aa7f9c6c0aeec9ad9045d3fc5 | d__Bacteria; p__Actinomycetota; c__Actinobacteria; o__Micrococcales; f__Micrococcaceae | Grass |  |
| 4d88d33af08d92f17d6fbc43bfd564cf | d__Bacteria; p__Actinomycetota; c__Actinobacteria; o__Micrococcales; f__Micrococcaceae | Grass |  |
| 5f6fad4f2203f79f4c64e5b768493e47 | d__Bacteria; p__Actinomycetota; c__Actinobacteria; o__Micrococcales; f__Micrococcaceae | Grass |  |
| 9da03dc913553fd68a0eeb2d1fa38e67 | d__Bacteria; p__Actinomycetota; c__Actinobacteria; o__Micrococcales; f__Micrococcaceae | Grass | Highly correlated |
| 9fb4449c3d70aa7b60c01e82caecae62 | d__Bacteria; p__Actinomycetota; c__Actinobacteria; o__Micrococcales; f__Micrococcaceae | Grass |  |
| b280d4a8377d1c69dd46ae56f66d791d | d__Bacteria; p__Actinomycetota; c__Actinobacteria; o__Micrococcales; f__Micrococcaceae | Grass |  |
| b52b5e275d3470329832efa1b1da10d5 | d__Bacteria; p__Actinomycetota; c__Actinobacteria; o__Micrococcales; f__Micrococcaceae | Grass | Highly correlated |
| b8dc66be558fe35ae5b25305cd90bf3c | d__Bacteria; p__Actinomycetota; c__Actinobacteria; o__Micrococcales; f__Micrococcaceae | Grass |  |
| bd288740266c4423b7e8d9b9c9ff49c7 | d__Bacteria; p__Actinomycetota; c__Actinobacteria; o__Micrococcales; f__Micrococcaceae | Grass |  |
| cdbeb740cfa16e12955e2064d58646fe | d__Bacteria; p__Actinomycetota; c__Actinobacteria; o__Micrococcales; f__Micrococcaceae | Grass |  |
| f4873b84ebaf60c98a6da81c9a2cb123 | d__Bacteria; p__Actinomycetota; c__Actinobacteria; o__Micrococcales; f__Micrococcaceae | Grass |  |
| 0d282fdcf78a904140b245ab6a88af1c | d__Bacteria; p__Actinomycetota; c__Actinobacteria; o__Propionibacteriales; f__Nocardioidaceae; g__Aeromicrobium; s__uncultured_bacterium | Grass |  |
| 1088c11e3a61a27056e707591f88d2a4 | d__Bacteria; p__Actinomycetota; c__Actinobacteria; o__Propionibacteriales; f__Nocardioidaceae; g__Aeromicrobium; s__uncultured_bacterium | Grass |  |
| 288e29bf330204041a1856961383794a | d__Bacteria; p__Actinomycetota; c__Actinobacteria; o__Propionibacteriales; f__Nocardioidaceae; g__Aeromicrobium; s__uncultured_bacterium | Grass |  |
| 28ff08de62561e79b54e2163a32db636 | d__Bacteria; p__Actinomycetota; c__Actinobacteria; o__Propionibacteriales; f__Nocardioidaceae; g__Aeromicrobium; s__uncultured_bacterium | Grass |  |
| 35955ad1e24f4f09736019f760ec83d8 | d__Bacteria; p__Actinomycetota; c__Actinobacteria; o__Propionibacteriales; f__Nocardioidaceae; g__Aeromicrobium; s__uncultured_bacterium | Grass |  |
| 98048f8493fdd8133c22873711b409a9 | d__Bacteria; p__Actinomycetota; c__Actinobacteria; o__Propionibacteriales; f__Nocardioidaceae; g__Aeromicrobium; s__uncultured_bacterium | Grass |  |
| 9c1b2485bafba0b5e8d9c01cd65439a3 | d__Bacteria; p__Actinomycetota; c__Actinobacteria; o__Propionibacteriales; f__Nocardioidaceae; g__Aeromicrobium; s__uncultured_bacterium | Grass |  |
| cd12d5d94b73c4fad1484fa7673f2024 | d__Bacteria; p__Actinomycetota; c__Actinobacteria; o__Propionibacteriales; f__Nocardioidaceae; g__Aeromicrobium; s__uncultured_bacterium | Grass |  |
| ecbec26714703cab1c43c15d344473eb | d__Bacteria; p__Actinomycetota; c__Actinobacteria; o__Propionibacteriales; f__Nocardioidaceae; g__Aeromicrobium; s__uncultured_bacterium | Grass |  |
| faa1af6289493a055624a26d3b5d1191 | d__Bacteria; p__Actinomycetota; c__Actinobacteria; o__Propionibacteriales; f__Nocardioidaceae; g__Aeromicrobium; s__uncultured_bacterium | Grass |  |
| 3dcf23923f59de26b6d6f64656855bfc | d__Bacteria; p__Actinomycetota; c__Actinobacteria; o__Propionibacteriales; f__Propionibacteriaceae; g__Marinilutecoccus | Grass |  |
| 40adc68f73e151e9291eee3c860e7bea | d__Bacteria; p__Actinomycetota; c__Coriobacteriia; o__Coriobacteriales; f__Coriobacteriales_Incertae_Sedis; g__Raoultibacter; s__metagenome | Grass |  |
| 5ad1d20c8077148e492803f5ac520134 | d__Bacteria; p__Actinomycetota; c__Coriobacteriia; o__Coriobacteriales; f__Coriobacteriales_Incertae_Sedis; g__Raoultibacter; s__metagenome | Grass |  |
| c018be3b9bf70152ae7f0b001607212c | d__Bacteria; p__Actinomycetota; c__Coriobacteriia; o__Coriobacteriales; f__Coriobacteriales_Incertae_Sedis; g__Raoultibacter; s__metagenome | Grass |  |
| 6648b5d7aa45b201191cd54b66d9586c | d__Bacteria; p__Actinomycetota; c__Coriobacteriia; o__Coriobacteriales; f__Eggerthellaceae; g__DNF00809; s__uncultured_bacterium | Grass |  |
| 7e206a4453cd99b6d23abe21fecb0834 | d__Bacteria; p__Cyanobacteria; c__Cyanobacteriia | Grass |  |
| 01da355d530e24f7b62316b89e901e3c | d__Bacteria; p__Bacillota; c__Clostridia; o__Christensenellales; f__Christensenellaceae; g__Christensenellaceae_R-7_group; s__uncultured_bacterium | Grass |  |
| 09f59505f3e620c48fb3fdd1c136dce0 | d__Bacteria; p__Bacillota; c__Clostridia; o__Christensenellales; f__Christensenellaceae; g__Christensenellaceae_R-7_group; s__uncultured_bacterium | Grass |  |
| bd8664d9a09ee649571271b30a61fc0c | d__Bacteria; p__Bacillota; c__Clostridia; o__Christensenellales; f__Christensenellaceae; g__Christensenellaceae_R-7_group; s__uncultured_bacterium | Grass |  |
| 1080556944639fdf696ee04a03843565 | d__Bacteria; p__Bacillota; c__Clostridia; o__Clostridiales; f__Clostridiaceae; g__Proteiniclasticum; s__uncultured_microorganism | Grass |  |
| 3cd4b958cb9d1b1c615ba778a67ffb0e | d__Bacteria; p__Bacillota; c__Clostridia; o__Clostridiales; f__Clostridiaceae; g__Proteiniclasticum; s__uncultured_microorganism | Grass |  |
| 65fe886a793a05023fadd26d5a0498c3 | d__Bacteria; p__Bacillota; c__Clostridia; o__Clostridiales; f__Clostridiaceae; g__Proteiniclasticum; s__uncultured_microorganism | Grass |  |
| 67766a52fbae24b1bd4071683894bcf3 | d__Bacteria; p__Bacillota; c__Clostridia; o__Clostridiales; f__Clostridiaceae; g__Proteiniclasticum; s__uncultured_microorganism | Grass |  |
| a5d5301a6434e6a33f00e406fd83956a | d__Bacteria; p__Bacillota; c__Clostridia; o__Clostridiales; f__Clostridiaceae; g__Proteiniclasticum; s__uncultured_microorganism | Grass |  |
| 82bb5ca3f9c987e40479027377763399 | d__Bacteria; p__Bacillota; c__Clostridia; o__Lachnospirales | Grass |  |
| 8c0380f499997ca80a1b3a49f0c6e2cd | d__Bacteria; p__Bacillota; c__Clostridia; o__Lachnospirales | Grass |  |
| a790290a14a98966e369bb88501a76de | d__Bacteria; p__Bacillota; c__Clostridia; o__Lachnospirales | Grass |  |
| c8994f1d8ab42f361ae14d02babb5812 | d__Bacteria; p__Bacillota; c__Clostridia; o__Lachnospirales | Grass |  |
| 280c167e5c3bfbb34f28b1a70b373176 | d__Bacteria; p__Bacillota; c__Clostridia; o__Lachnospirales; f__Lachnospiraceae | Grass |  |
| 4335af64dd99843b741d277c2b92d63c | d__Bacteria; p__Bacillota; c__Clostridia; o__Lachnospirales; f__Lachnospiraceae | Grass |  |
| 78e8bfd015f971a97d70005d45e43b10 | d__Bacteria; p__Bacillota; c__Clostridia; o__Lachnospirales; f__Lachnospiraceae | Grass |  |
| 962e48345a2d71d383b3fdc3c18b269a | d__Bacteria; p__Bacillota; c__Clostridia; o__Lachnospirales; f__Lachnospiraceae | Grass |  |
| 96e9bc518503bd70c16f65466b217f06 | d__Bacteria; p__Bacillota; c__Clostridia; o__Lachnospirales; f__Lachnospiraceae | Grass |  |
| a79f6ffa11e5e3b77e1440a0d4c6cebd | d__Bacteria; p__Bacillota; c__Clostridia; o__Lachnospirales; f__Lachnospiraceae | Grass |  |
| e2bc8b86648a522ca1bfb95de6402474 | d__Bacteria; p__Bacillota; c__Clostridia; o__Lachnospirales; f__Lachnospiraceae | Grass |  |
| e3e944630cd6c9fe525ee01309cd38a5 | d__Bacteria; p__Bacillota; c__Clostridia; o__Lachnospirales; f__Lachnospiraceae | Grass |  |
| fb5c08d77d4f7dbb62d92953ab2734d5 | d__Bacteria; p__Bacillota; c__Clostridia; o__Lachnospirales; f__Lachnospiraceae | Grass |  |
| fdccc12daf14afd7f46d5f6ec6cd2813 | d__Bacteria; p__Bacillota; c__Clostridia; o__Lachnospirales; f__Lachnospiraceae | Grass |  |
| 2dde39066b6242a11928cf6e7cde1fa7 | d__Bacteria; p__Bacillota; c__Clostridia; o__Lachnospirales; f__Lachnospiraceae; g__Tyzzerella; s__uncultured_bacterium | Grass |  |
| 50ae4d4f359878a5256329528b6d8a34 | d__Bacteria; p__Bacillota; c__Clostridia; o__Lachnospirales; f__Lachnospiraceae; g__Tyzzerella; s__uncultured_bacterium | Grass |  |
| 5906b1858554b9f2f4c7c2005a5785cc | d__Bacteria; p__Bacillota; c__Clostridia; o__Lachnospirales; f__Lachnospiraceae; g__Tyzzerella; s__uncultured_bacterium | Grass |  |
| 7564bd7bfd730e465a9aacb73fe60cce | d__Bacteria; p__Bacillota; c__Clostridia; o__Lachnospirales; f__Lachnospiraceae; g__Tyzzerella; s__uncultured_bacterium | Grass |  |
| d6202e4a56332e24735f52acf3851257 | d__Bacteria; p__Bacillota; c__Clostridia; o__Lachnospirales; f__Lachnospiraceae; g__Tyzzerella; s__uncultured_bacterium | Grass |  |
| fa828ec04f48d6fae81b399a77e0a28f | d__Bacteria; p__Bacillota; c__Clostridia; o__Lachnospirales; f__Lachnospiraceae; g__Tyzzerella; s__uncultured_bacterium | Grass |  |
| 24669b1d9656c7fd1d36a39787ec538d | d__Bacteria; p__Bacillota; c__Clostridia; o__Oscillospirales; f__Ruminococcaceae; g__Candidatus_Soleaferrea; s__uncultured_bacterium | Grass |  |
| 248a3f80105488d0738e9623b7787d45 | d__Bacteria; p__Bacillota; c__Clostridia; o__Oscillospirales; f__Ruminococcaceae; g__Candidatus_Soleaferrea; s__uncultured_bacterium | Grass |  |
| 40865bfda29ee59e6b58a4af70502951 | d__Bacteria; p__Bacillota; c__Clostridia; o__Oscillospirales; f__Ruminococcaceae; g__Candidatus_Soleaferrea; s__uncultured_bacterium | Grass |  |
| 435df76d3b27e5401e3953037dac9834 | d__Bacteria; p__Bacillota; c__Clostridia; o__Oscillospirales; f__Ruminococcaceae; g__Candidatus_Soleaferrea; s__uncultured_bacterium | Grass |  |
| 4e571588707e150f54c40ac31f337f06 | d__Bacteria; p__Bacillota; c__Clostridia; o__Oscillospirales; f__Ruminococcaceae; g__Candidatus_Soleaferrea; s__uncultured_bacterium | Grass |  |
| 67650cfac7eea5b3932efec56d465838 | d__Bacteria; p__Bacillota; c__Clostridia; o__Oscillospirales; f__Ruminococcaceae; g__Candidatus_Soleaferrea; s__uncultured_bacterium | Grass |  |
| 9e752af40803892c50860b573bea0eeb | d__Bacteria; p__Bacillota; c__Clostridia; o__Oscillospirales; f__Ruminococcaceae; g__Candidatus_Soleaferrea; s__uncultured_bacterium | Grass |  |
| a81a908214aa45369f36a8580ae1b42f | d__Bacteria; p__Bacillota; c__Clostridia; o__Oscillospirales; f__Ruminococcaceae; g__Candidatus_Soleaferrea; s__uncultured_bacterium | Grass |  |
| ab18122506ad048ea459818412c00ba5 | d__Bacteria; p__Bacillota; c__Clostridia; o__Oscillospirales; f__Ruminococcaceae; g__Candidatus_Soleaferrea; s__uncultured_bacterium | Grass |  |
| b414afb74a573bc3c453a42f80e743fd | d__Bacteria; p__Bacillota; c__Clostridia; o__Oscillospirales; f__Ruminococcaceae; g__Candidatus_Soleaferrea; s__uncultured_bacterium | Grass |  |
| b4e558e1700b5cd9b96969282c04dd57 | d__Bacteria; p__Bacillota; c__Clostridia; o__Oscillospirales; f__Ruminococcaceae; g__Candidatus_Soleaferrea; s__uncultured_bacterium | Grass |  |
| c38ac24cae4fb4a4e4df95c8dd2f5ec0 | d__Bacteria; p__Bacillota; c__Clostridia; o__Oscillospirales; f__Ruminococcaceae; g__Candidatus_Soleaferrea; s__uncultured_bacterium | Grass |  |
| e2d271d78cd6be464cc9436c157a1200 | d__Bacteria; p__Bacillota; c__Clostridia; o__Oscillospirales; f__Ruminococcaceae; g__Candidatus_Soleaferrea; s__uncultured_bacterium | Grass |  |
| e678b5cc8bbee5d93dec70743042cd23 | d__Bacteria; p__Bacillota; c__Clostridia; o__Oscillospirales; f__Ruminococcaceae; g__Candidatus_Soleaferrea; s__uncultured_bacterium | Grass |  |
| f71f42b0d3af4245fb7d06eb8e40a65e | d__Bacteria; p__Bacillota; c__Clostridia; o__Oscillospirales; f__Ruminococcaceae; g__Candidatus_Soleaferrea; s__uncultured_bacterium | Grass |  |
| ff7d3a04dc5953b25164037331c8fb3f | d__Bacteria; p__Bacillota; c__Clostridia; o__Oscillospirales; f__Ruminococcaceae; g__Candidatus_Soleaferrea; s__uncultured_bacterium | Grass |  |
| 10c2299ba4d9492978db67f898dd82a3 | d__Bacteria; p__Bacillota; c__Clostridia; o__Peptostreptococcales-Tissierellales; f__Anaerovoracaceae; g__Anaerovorax; s__uncultured_Bacillota | Grass |  |
| 31e28aab6c0671192d49b3099abfb175 | d__Bacteria; p__Bacillota; c__Clostridia; o__Peptostreptococcales-Tissierellales; f__Anaerovoracaceae; g__Anaerovorax; s__uncultured_Bacillota | Grass |  |
| be3fab95d8ee03100126df35b7e5c171 | d__Bacteria; p__Bacillota; c__Clostridia; o__Peptostreptococcales-Tissierellales; f__Anaerovoracaceae; g__Anaerovorax; s__uncultured_Bacillota | Grass |  |
| c80a43b3c4c63b05c85df99c1dde46d5 | d__Bacteria; p__Bacillota; c__Clostridia; o__Peptostreptococcales-Tissierellales; f__Anaerovoracaceae; g__Anaerovorax; s__uncultured_Bacillota | Grass |  |
| e414781df65464bb2a8e9cc0c8135710 | d__Bacteria; p__Bacillota; c__Clostridia; o__Peptostreptococcales-Tissierellales; f__Anaerovoracaceae; g__Anaerovorax; s__uncultured_Bacillota | Grass |  |
| 20cb6c414e68a556d4af95f2518cdc0b | d__Bacteria; p__Bacillota; c__Clostridia; o__Peptostreptococcales-Tissierellales; f__Peptostreptococcaceae | Grass |  |
| 42dbf94e74245b363b7a469f04b489fe | d__Bacteria; p__Bacillota; c__Clostridia; o__Peptostreptococcales-Tissierellales; f__Peptostreptococcaceae | Grass |  |
| 5af7bba308dad663a1ef99a84d6b5071 | d__Bacteria; p__Bacillota; c__Clostridia; o__Peptostreptococcales-Tissierellales; f__Peptostreptococcaceae | Grass |  |
| 9ed41755b5732c51632d79860f619e60 | d__Bacteria; p__Bacillota; c__Clostridia; o__Peptostreptococcales-Tissierellales; f__Peptostreptococcaceae | Grass |  |
| b6fdf40b09812f65c19080141a0fcf2d | d__Bacteria; p__Bacillota; c__Clostridia; o__Peptostreptococcales-Tissierellales; f__Peptostreptococcaceae | Grass |  |
| d349727f5f3d8878239412bd0daff0ea | d__Bacteria; p__Bacillota; c__Clostridia; o__Peptostreptococcales-Tissierellales; f__Peptostreptococcaceae | Grass |  |
| 57723a89c2e126ef8b196f1643417c0a | d__Bacteria; p__Bacillota; c__Clostridia; o__Peptostreptococcales-Tissierellales; f__Peptostreptococcaceae; g__uncultured; s__uncultured_bacterium | Grass |  |
| 300bf2323a79f9d0f72ba98aa515336c | d__Bacteria; p__Bacillota; c__Clostridia; o__Peptostreptococcales-Tissierellales; f__Peptostreptococcales-Tissierellales; g__Gallicola; s__uncultured_bacterium | Grass | Highly correlated |
| 33bc50ca36a4e5adcf4372d4c2995fef | d__Bacteria; p__Bacillota; c__Clostridia; o__Peptostreptococcales-Tissierellales; f__Peptostreptococcales-Tissierellales; g__Gallicola; s__uncultured_bacterium | Grass | Highly correlated |
| 8082f88a5e05bb0433b6a0f25a412611 | d__Bacteria; p__Bacillota; c__Clostridia; o__Peptostreptococcales-Tissierellales; f__Peptostreptococcales-Tissierellales; g__Gallicola; s__uncultured_bacterium | Grass | Highly correlated |
| b07ded419d52e7e435d9b2af2fc1766d | d__Bacteria; p__Bacillota; c__Clostridia; o__Peptostreptococcales-Tissierellales; f__Peptostreptococcales-Tissierellales; g__Gallicola; s__uncultured_bacterium | Grass | Highly correlated |
| d029379a15c591fc0a96213a6f8d4ccf | d__Bacteria; p__Bacillota; c__Clostridia; o__Peptostreptococcales-Tissierellales; f__Peptostreptococcales-Tissierellales; g__Gallicola; s__uncultured_bacterium | Grass | Highly correlated |
| 39d7dd9c49977ba6bc68eabbb60649b9 | d__Bacteria; p__Bacillota; c__Clostridia; o__Peptostreptococcales-Tissierellales; f__Peptostreptococcales-Tissierellales; g__Tissierella; s__uncultured_bacterium | Grass |  |
| 5684ea722748629c7c98dac3b3af49e7 | d__Bacteria; p__Psuedomonadota; c__AlphaPsuedomonadota; o__Rhizobiales; f__Rhizobiaceae; g__Ensifer | Grass |  |
| a24f88e09243ef9d559d16f1f05edd6a | d__Bacteria; p__Psuedomonadota; c__AlphaPsuedomonadota; o__Rhizobiales; f__Rhizobiaceae; g__Ensifer | Grass |  |

| Table S2 Full list of taxa that are highly correlated with each respective diet group | | |
| --- | --- | --- |
| Highly correlated Feature ID | Highly correlated Taxon | Highly correlated Diet group |
| 717520341cc529dff78c58790d4cabc3 | d__Bacteria; p__Bacillota; c__Clostridia; o__Peptostreptococcales-Tissierellales; f__Peptostreptococcaceae; g__uncultured; s__uncultured_bacterium | Silage |
| e42d24cfb93825366121de08f3c9fe98 | d__Bacteria; p__Bacillota; c__Clostridia; o__Peptostreptococcales-Tissierellales; f__Peptostreptococcaceae; g__Romboutsia | Silage |
| 0f69faca7e09c24ee80045bb81994af7 | d__Bacteria; p__Bacillota; c__Clostridia; o__Peptostreptococcales-Tissierellales; f__Peptostreptococcaceae; g__Romboutsia | Silage |
| 6f6b0e4360aa298e5a52c5b0802f4928 | d__Bacteria; p__Bacillota; c__Clostridia; o__Peptostreptococcales-Tissierellales; f__Peptostreptococcaceae; g__Romboutsia | Silage |
| a11a48f918586ae3f8356fc0a04e5051 | d__Bacteria; p__Bacillota; c__Clostridia; o__Peptostreptococcales-Tissierellales; f__Peptostreptococcaceae; g__Romboutsia | Silage |
| 8a9affc852986d47ab84940b8aa44b4f | d__Bacteria; p__Bacillota; c__Clostridia; o__Lachnospirales; f__Lachnospiraceae; g__Marvinbryantia; s__uncultured_bacterium | Silage |
| 7f43a9636651e4f067e044f954955ed8 | d__Bacteria; p__Psuedomonadota; c__AlphaPsuedomonadota; o__Rhizobiales; f__Devosiaceae; g__Devosia; s__Devosia_riboflavina | Silage |
| c2b84ddc945db7eb466a2f2eb4566e79 | d__Bacteria; p__Psuedomonadota; c__AlphaPsuedomonadota; o__Rhizobiales; f__Devosiaceae; g__Devosia; s__Devosia_riboflavina | Silage |
| d27c0126f73548870f42db6a77b45893 | d__Bacteria; p__Bacillota; c__Clostridia; o__Lachnospirales; f__Lachnospiraceae; g__Lachnoclostridium; s__unidentified | Silage |
| 4df0be2a4643acbf425b560ba2e384cd | d__Bacteria; p__Actinomycetota; c__Actinobacteria; o__Corynebacteriales; f__Dietziaceae; g__Dietzia; s__Dietzia_sp. | Silage |
| c407ff3fa3eabee4c892774eec771133 | d__Bacteria; p__Actinomycetota; c__Actinobacteria; o__Corynebacteriales; f__Nocardiaceae; g__Rhodococcus; s__Rhodococcus_rhodochrous | Silage |
| 83f0d5b3ced211875a7a2327b9b5679f | d__Bacteria; p__Bacillota; c__Clostridia; o__Clostridiales; f__Clostridiaceae; g__Clostridium_sensu_stricto_1; s__uncultured_bacterium | Silage |
| 9ccff28c7245125a8ed518bf2bc33fc6 | d__Bacteria; p__Actinomycetota; c__Actinobacteria; o__Corynebacteriales; f__Dietziaceae; g__Dietzia; s__Dietzia_sp. | Silage |
| 1a00c433e734152c15b9daceb1bcc4f9 | d__Bacteria; p__Actinomycetota; c__Actinobacteria; o__Corynebacteriales; f__Dietziaceae; g__Dietzia; s__Dietzia_sp. | Silage |
| f8e4be8e90dc245aed2811c612d66f00 | d__Bacteria; p__Bacillota; c__Clostridia; o__Clostridiales; f__Clostridiaceae; g__Clostridium_sensu_stricto_1; s__uncultured_bacterium | Silage |
| 6013cb06aaa64d38178ee623e8a0360e | d__Bacteria; p__Actinomycetota; c__Actinobacteria; o__Corynebacteriales; f__Dietziaceae; g__Dietzia; s__Dietzia_sp. | Silage |
| 8f6d5a34c654393b8443db97864a4af5 | d__Bacteria; p__Bacillota; c__Clostridia; o__Peptostreptococcales-Tissierellales; f__Peptostreptococcaceae; g__Paeniclostridium | Silage |
| 9677ecf7f42953e2cc851d8e767b760f | d__Bacteria; p__Bacillota; c__Clostridia; o__Clostridiales; f__Clostridiaceae; g__Clostridium_sensu_stricto_13; s__bacterium_Te48R | Silage |
| 5284c8c48e47d9243d1a3a48683d6af6 | d__Bacteria; p__Bacillota; c__Clostridia; o__Lachnospirales; f__Lachnospiraceae; g__Anaerostignum; s__uncultured_Clostridium | Silage |
| 57cd751ba2ac376df90e6cdab8e71a2d | d__Bacteria; p__Actinomycetota; c__Actinobacteria; o__Corynebacteriales; f__Nocardiaceae; g__Rhodococcus; s__Rhodococcus_rhodochrous | Silage |
| 8559d8aaa5554ddd923fc46816662473 | d__Bacteria; p__Actinomycetota; c__Actinobacteria; o__Corynebacteriales; f__Nocardiaceae; g__Rhodococcus; s__Rhodococcus_rhodochrous | Silage |
| 17d5c3d6876708919e5199044fe8495d | d__Bacteria; p__Bacillota; c__Clostridia; o__Clostridiales; f__Clostridiaceae; g__Clostridium_sensu_stricto_1; s__uncultured_bacterium | Silage |
| 40e15bbedd6f50bdcbf8e8689bc0d24e | d__Bacteria; p__Bacillota; c__Clostridia; o__Clostridiales; f__Clostridiaceae; g__Clostridium_sensu_stricto_1; s__uncultured_bacterium | Silage |
| 3b464637eeca7f182743772189d9a1c7 | d__Bacteria; p__Bacillota; c__Clostridia; o__Clostridiales; f__Clostridiaceae; g__Clostridium_sensu_stricto_1; s__uncultured_bacterium | Silage |
| 001a6347c327d6fa510dc46cef264f03 | d__Bacteria; p__Bacillota; c__Clostridia; o__Clostridiales; f__Clostridiaceae; g__Clostridium_sensu_stricto_1; s__uncultured_bacterium | Silage |
| 788d56563ed7e8f4be895ae2ddc25288 | d__Bacteria; p__Bacillota; c__Clostridia; o__Peptostreptococcales-Tissierellales; f__Peptostreptococcaceae; g__Paeniclostridium | Silage |
| 6a54b20c2ec0304ed97779c81765a015 | d__Bacteria; p__Actinomycetota; c__Actinobacteria; o__Propionibacteriales; f__Propionibacteriaceae; g__Marinilutecoccus; s__unidentified | Hay |
| 836e4caf2b77b16e0904db4a2589a8a4 | d__Bacteria; p__Actinomycetota; c__Actinobacteria; o__Propionibacteriales; f__Propionibacteriaceae; g__Marinilutecoccus; s__unidentified | Hay |
| 58a7b7b1771ca643b0db8965fbf35136 | d__Bacteria; p__Actinomycetota; c__Actinobacteria; o__Propionibacteriales; f__Propionibacteriaceae; g__Marinilutecoccus; s__unidentified | Hay |
| 9f4a2d3ff0efc82892bdf059295012c5 | d__Bacteria; p__Actinomycetota; c__Actinobacteria; o__Propionibacteriales; f__Propionibacteriaceae; g__Marinilutecoccus; s__unidentified | Hay |
| 87fe22feff974805fb29464a01f9d07c | d__Bacteria; p__Actinomycetota; c__Actinobacteria; o__Propionibacteriales; f__Propionibacteriaceae; g__Marinilutecoccus; s__unidentified | Hay |
| cbcedad1fbfcf0e03fe9f287ba9d7613 | d__Bacteria; p__Actinomycetota; c__Actinobacteria; o__Propionibacteriales; f__Propionibacteriaceae; g__Marinilutecoccus; s__unidentified | Hay |
| 4f78a95348652c49f82d39751f416fb2 | d__Bacteria; p__Actinomycetota; c__Actinobacteria; o__Propionibacteriales; f__Propionibacteriaceae; g__Marinilutecoccus; s__unidentified | Hay |
| fa435e67f679b190843742ad87801606 | d__Bacteria; p__Actinomycetota; c__Actinobacteria; o__Propionibacteriales; f__Propionibacteriaceae; g__Marinilutecoccus; s__unidentified | Hay |
| 8e30dbec71b844ce6db3761d4708389a | d__Bacteria; p__Actinomycetota; c__Actinobacteria; o__Propionibacteriales; f__Propionibacteriaceae; g__Marinilutecoccus; s__unidentified | Hay |
| e9214d2f3d4ec7f8aeec52835e7ec655 | d__Bacteria; p__Actinomycetota; c__Actinobacteria; o__Propionibacteriales; f__Nocardioidaceae; g__Aeromicrobium; s__uncultured_bacterium | Hay |
| f63f4e22824d59e2ccb906e3e66e942e | d__Bacteria; p__Actinomycetota; c__Actinobacteria; o__Micrococcales; f__Micrococcaceae; g__Kocuria | Hay |
| 31ad0d6535de138825a8069032499963 | d__Bacteria; p__Actinomycetota; c__Actinobacteria; o__Micrococcales; f__Micrococcaceae; g__Kocuria | Hay |
| db8b461299effcb498bf2bfb5331fda0 | d__Bacteria; p__Actinomycetota; c__Actinobacteria; o__Micrococcales; f__Micrococcaceae; g__Kocuria | Hay |
| afcb367a60e7440f741a00edafa57ece | d__Bacteria; p__Actinomycetota; c__Actinobacteria; o__Micrococcales; f__Micrococcaceae; g__Kocuria | Hay |
| f51f0b642bc1b88fca5b97655c1bb0af | d__Bacteria; p__Actinomycetota; c__Acidimicrobiia; o__Microtrichales; f__Microtrichaceae; g__uncultured; s__uncultured_bacterium | Hay |
| e5268465761f4f85bbe06d49dff8af0b | d__Bacteria; p__Actinomycetota; c__Acidimicrobiia; o__Microtrichales; f__Microtrichaceae; g__uncultured; s__uncultured_bacterium | Hay |
| 399810bb299a88794dd66ad18af99db9 | d__Bacteria; p__Actinomycetota; c__Actinobacteria; o__Micrococcales; f__Micrococcaceae; g__Arthrobacter; s__Arthrobacter_sp. | Hay |
| 3880f0a107c7e9e3af4a85f7cf61e975 | d__Bacteria; p__Actinomycetota; c__Actinobacteria; o__Micrococcales; f__Micrococcaceae; g__Kocuria; s__Kocuria_flava | Hay |
| 965f80756270460d53ebd59d80291358 | d__Bacteria; p__Actinomycetota; c__Actinobacteria; o__Micrococcales; f__Micrococcaceae; g__Kocuria; s__Kocuria_flava | Hay |
| ab0935255d5e24b52fe55548fbd6331b | d__Bacteria; p__Actinomycetota; c__Actinobacteria; o__Micrococcales; f__Micrococcaceae; g__Kocuria; s__Kocuria_flava | Hay |
| becdabcbc16a242819a042dcb9b5988b | d__Bacteria; p__Actinomycetota; c__Actinobacteria; o__Micrococcales; f__Micrococcaceae; g__Kocuria; s__Kocuria_flava | Hay |
| 041d0969bd916270dc8df6f75fe23174 | d__Bacteria; p__Actinomycetota; c__Actinobacteria; o__Micrococcales; f__Micrococcaceae | Grass |
| b07ded419d52e7e435d9b2af2fc1766d | d__Bacteria; p__Bacillota; c__Clostridia; o__Peptostreptococcales-Tissierellales; f__Peptostreptococcales-Tissierellales; g__Gallicola; s__uncultured_bacterium | Grass |
| b52b5e275d3470329832efa1b1da10d5 | d__Bacteria; p__Actinomycetota; c__Actinobacteria; o__Micrococcales; f__Micrococcaceae | Grass |
| d029379a15c591fc0a96213a6f8d4ccf | d__Bacteria; p__Bacillota; c__Clostridia; o__Peptostreptococcales-Tissierellales; f__Peptostreptococcales-Tissierellales; g__Gallicola; s__uncultured_bacterium | Grass |
| 300bf2323a79f9d0f72ba98aa515336c | d__Bacteria; p__Bacillota; c__Clostridia; o__Peptostreptococcales-Tissierellales; f__Peptostreptococcales-Tissierellales; g__Gallicola; s__uncultured_bacterium | Grass |
| 33bc50ca36a4e5adcf4372d4c2995fef | d__Bacteria; p__Bacillota; c__Clostridia; o__Peptostreptococcales-Tissierellales; f__Peptostreptococcales-Tissierellales; g__Gallicola; s__uncultured_bacterium | Grass |
| 9da03dc913553fd68a0eeb2d1fa38e67 | d__Bacteria; p__Actinomycetota; c__Actinobacteria; o__Micrococcales; f__Micrococcaceae | Grass |
| 8082f88a5e05bb0433b6a0f25a412611 | d__Bacteria; p__Bacillota; c__Clostridia; o__Peptostreptococcales-Tissierellales; f__Peptostreptococcales-Tissierellales; g__Gallicola; s__uncultured_bacterium | Grass |

| Table S3 Kegg Orthologs (KOs) significantly enriched or depleted in the hindgut microbiomes of hay dung fed larvae compared to grass dung fed larvae | | | |
| --- | --- | --- | --- |
| KO ID | Enriched in Hay Dung relative to Grass Dung Larvae | KO pathway Name | KO pathway description |
| K17498 | enriched | transcription factor SPN1 |  |
| K19479 | enriched | collagen type X alpha |  |
| K16695 | enriched | lipopolysaccharide exporter |  |
| K09487 | enriched | HSP90B, TRA1; heat shock protein 90kDa beta | stress response perhaps to adaptation to hay dung |
| K14683 | enriched | SLC34A, NPT, nptA; solute carrier family 34 (sodium-dependent phosphate cotransporter) | sodium-phosphate cotransporter-microbial roles in phosphate uptake and transport |
| K10750 | depleted | CHAF1A; chromatin assembly factor 1 subunit A | transcription/translation machinery |
| K15320 | depleted | ATX, chlB1, mdpB; 6-methylsalicylic acid synthase [EC:2.3.1.165] | polyketide and fatty acid metabolism |
| K16191 | depleted | arfA; peptidoglycan-binding protein ArfA |  |
| K14651 | depleted | TAF15, NPL3; transcription initiation factor TFIID subunit 15 | transcription/translation machinery |
| K03258 | depleted | translation initiation factor 4B | transcription/translation machinery |
| K00545 | depleted | catechol O-methyltransferase [EC:2.1.1.6] |  |
| K16045 | depleted | hsd; 3beta-hydroxy-Delta5-steroid dehydrogenase / steroid Delta-isomerase [EC:1.1.1.145 5.3.3.1] | steroid metabolism |
| K12423 | depleted | fadD21; fatty acid CoA ligase FadD21 | polyketide and fatty acid metabolism |
| K14215 | depleted | E2.5.1.86; trans,polycis-decaprenyl diphosphate synthase [EC:2.5.1.86] | |
| K01909 | depleted | mbtM; long-chain-fatty-acid--[acyl-carrier-protein] ligase [EC:6.2.1.20] | polyketide and fatty acid metabolism |
| K16920 | depleted | ytrE; acetoin utilization transport system ATP-binding protein |  |
| K12433 | depleted | pks5; polyketide synthase 5 | polyketide and fatty acid metabolism |
| K18673 | depleted | bglK; beta-glucoside kinase [EC:2.7.1.85] | carbohydrate processsing: involved in plant sugar metabolism |
| K18957 | depleted | whiB6; WhiB family transcriptional regulator, redox-sensing transcriptional regulator | |

| Table 4 Kegg Orthologs (KOs) significantly enriched or depleted in the hindgut microbiomes of silage dung fed larvae compared to grass dung fed larvae | | |
| --- | --- | --- |
| KO ID | Enriched | KO Name |
| K04805 | enriched | CHRNA3; nicotinic acetylcholine receptor alpha-3 |
| K14573 | enriched | NOP4, RBM28; nucleolar protein 4 |
| K09554 | enriched | CDC37; cell division cycle protein 37 |
| K12879 | enriched | THOC2; THO complex subunit 2 |
| K15062 | enriched | ligY; OH-DDVA meta-cleavage compound hydrolase |
| K18315 | enriched | cpmC, carC; (5R)-carbapenem-3-carboxylate synthase [EC:1.14.20.3] |
| K18583 | enriched | thnE; carboxymethylproline synthase [EC:2.3.1.226] |
| K15966 | enriched | mtmOIV; monooxygenase [EC:1.14.13.-] |
| K13580 | enriched | K13580; magnesium chelatase subunit ChlD-like protein |
| K16019 | enriched | rifH; 3,4-Dideoxy-4-amino-D-arabino-heptulosonate 7-phosphate synthase |
| K01514 | enriched | PRUNE, PPX1; exopolyphosphatase [EC:3.6.1.11] |
| K15058 | enriched | amnA; 2-aminophenol/2-amino-5-chlorophenol 1,6-dioxygenase subunit alpha |
| K15059 | enriched | amnB; 2-aminophenol/2-amino-5-chlorophenol 1,6-dioxygenase subunit beta [EC:1.13.11.74 1.13.11.76] |
| K02505 | depleted | hofC; protein transport protein HofC |
| K09890 | depleted | arfA; alternative ribosome-rescue factor |
| K00543 | depleted | ASMT; acetylserotonin O-methyltransferase [EC:2.1.1.4] |
| K14057 | depleted | abgR; LysR family transcriptional regulator, regulator of abg operon |
| K10015 | depleted | hisM; histidine transport system permease protein |
| K02565 | depleted | nagC; N-acetylglucosamine repressor |
| K18252 | depleted | phtAb; phthalate 3,4-dioxygenase subunit beta [EC:1.14.12.-] |
| K12963 | depleted | arnF; undecaprenyl phosphate-alpha-L-ara4N flippase subunit ArnF |
| K15643 | depleted | mxaB; myxalamid-type polyketide synthase MxaB |
| K09612 | depleted | iap; alkaline phosphatase isozyme conversion protein [EC:3.4.11.-] |
| K06149 | depleted | uspA; universal stress protein A |
| K05984 | depleted | cho; excinuclease Cho [EC:3.1.25.-] |
| K06879 | depleted | queF; 7-cyano-7-deazaguanine reductase [EC:1.7.1.13] |
| K10750 | depleted | CHAF1A; chromatin assembly factor 1 subunit A |
| K07311 | depleted | ynfG; Tat-targeted selenate reductase subunit YnfG |
| K00329 | depleted | NADH dehydrogenase |
| K02852 | depleted | wecG, rffM; UDP-N-acetyl-D-mannosaminouronate:lipid I N-acetyl-D-mannosaminouronosyltransferase [EC:2.4.1.180] |
| K16152 | depleted | hasR; heme acquisition protein HasR |
| K07310 | depleted | ynfF; Tat-targeted selenate reductase subunit YnfF [EC:1.97.1.9] |
| K09893 | depleted | rraB; regulator of ribonuclease activity B |
| K09997 | depleted | artI; arginine transport system substrate-binding protein |
| K00183 | depleted | adenosine 5'-monophosphate phosphohydrolase |
| K02705 | depleted | psbC; photosystem II CP43 chlorophyll apoprotein |
| K05952 | depleted | K05952; uncharacterized protein |
| K07687 | depleted | rcsB; two-component system, NarL family, captular synthesis response regulator RcsB |
| K07109 | depleted | K07109; uncharacterized protein |
| K07312 | depleted | ynfH; Tat-targeted selenate reductase subunit YnfH |
| K12537 | depleted | hasE, prtE, rsaE, prsE, eexE; membrane fusion protein, type I secretion system |
| K13009 | depleted | wzy; O-antigen polymerase [EC:2.4.99.27] |
| K07702 | depleted | dpiA, citB; two-component system, CitB family, response regulator CitB |
| K05594 | depleted | elaB; ElaB protein |
| K12974 | depleted | lpxP; KDO2-lipid IV(A) palmitoleoyltransferase [EC:2.3.1.242] |
| K02345 | depleted | holE; DNA polymerase III subunit theta [EC:2.7.7.7] |
| K07781 | depleted | rcsA; LuxR family transcriptional regulator, capsular biosynthesis positive transcription factor |
| K11929 | depleted | phoE; outer membrane pore protein E |
| K08308 | depleted | mltE, emtA; peptidoglycan lytic transglycosylase E [EC:4.2.2.29] |
| K07345 | depleted | fimA; major type 1 subunit fimbrin (pilin) |
| K08087 | depleted | fimW; fimbrial protein FimW |
| K19734 | depleted | expR; LuxR family transcriptional regulator, quorum-sensing system regulator ExpR |
| K05804 | depleted | rob; AraC family transcriptional regulator, mar-sox-rob regulon activator |
| K16127 | depleted | mcyG; microcystin synthetase protein McyG |
| K09470 | depleted | puuA; gamma-glutamylputrescine synthase [EC:6.3.1.11] |
| K10774 | depleted | E4.3.1.23; tyrosine ammonia-lyase [EC:4.3.1.23] |
| K18919 | depleted | hokC_D; protein HokC/D |

| Table S5 Predicted KEGG pathways related to fermentation in the larval hindgut | | | | | |  |
| --- | --- | --- | --- | --- | --- | --- |
| KO | Predicted KEGG abundance | | | Name | SCFA Production | |
|  | All Grass | All Hay | All Silage |  |  | |
| K00625 | 5933.81155 | 4334.6881 | 3563.45742 | phosphate acetyltransferase | Acetate Production | |
| K00925 | 9328.20264 | 7563.60188 | 7377.12992 | acetate kinase | Acetate Production | |
| K00156 | 2342.12845 | 3085.3374 | 2774.00594 | pyruvate dehydrogenase | Acetate Production | |
| K00158 | 106.650382 | 94.3230538 | 93.4323932 | pyruvate oxidase | Acetate Production | |
| K00929 | 2726.02358 | 997.01067 | 2239.61687 | butyrate kinase | Butyrate | |
| K00634 | 2521.97421 | 993.636445 | 2109.04108 | phosphate butyryltransferase | Butyrate | |
| K01847 | 3133.93952 | 3090.49635 | 3203.10224 | methylmalonyl-CoA mutase | Propionate | |
| K01848 | 1277.26082 | 1219.62865 | 1257.62799 | methylmalonyl-CoA mutase, N-terminal domain | Propionate | |
| K01849 | 1158.8978 | 1051.08315 | 1200.12198 | methylmalonyl-CoA mutase, C-terminal domain | Propionate | |
| K00016 | 7012.65877 | 6763.07292 | 6504.77 | L-lactate dehydrogenase | Propionate | |
